# Two Years into the COVID-19 Pandemic: Lessons Learned

**DOI:** 10.1021/acsinfecdis.2c00204

**Published:** 2022-08-08

**Authors:** Severino Jefferson Ribeiro da Silva, Jessica Catarine Frutuoso do Nascimento, Renata Pessôa Germano Mendes, Klarissa Miranda Guarines, Caroline Targino Alves da Silva, Poliana Gomes da Silva, Jurandy Júnior Ferraz de Magalhães, Justin R. J. Vigar, Abelardo Silva-Júnior, Alain Kohl, Keith Pardee, Lindomar Pena

**Affiliations:** † Laboratory of Virology and Experimental Therapy (LAVITE), Department of Virology, Aggeu Magalhães Institute (IAM), Oswaldo Cruz Foundation (Fiocruz), 50670-420 Recife, Pernambuco, Brazil; ‡ Department of Pharmaceutical Sciences, Leslie Dan Faculty of Pharmacy, University of Toronto, Toronto, ON M5S 3M2, Canada; § Department of Virology, Pernambuco State Central Laboratory (LACEN/PE), 52171-011 Recife, Pernambuco, Brazil; ∥ University of Pernambuco (UPE), Serra Talhada Campus, 56909-335 Serra Talhada, Pernambuco, Brazil; ⊥ Public Health Laboratory of the XI Regional Health, 56912-160 Serra Talhada, Pernambuco, Brazil; # Institute of Biological and Health Sciences, Federal University of Alagoas (UFAL), 57072-900 Maceió, Alagoas, Brazil; ∇ MRC-University of Glasgow Centre for Virus Research, Glasgow G61 1QH, United Kingdom; ○ Department of Mechanical and Industrial Engineering, University of Toronto, Toronto, ON M5S 3G8, Canada

**Keywords:** clinical features, diagnosis, SARS-CoV-2, variants, transmission, treatment, vaccines, reservoir hosts, pathophysiology, prevention

## Abstract

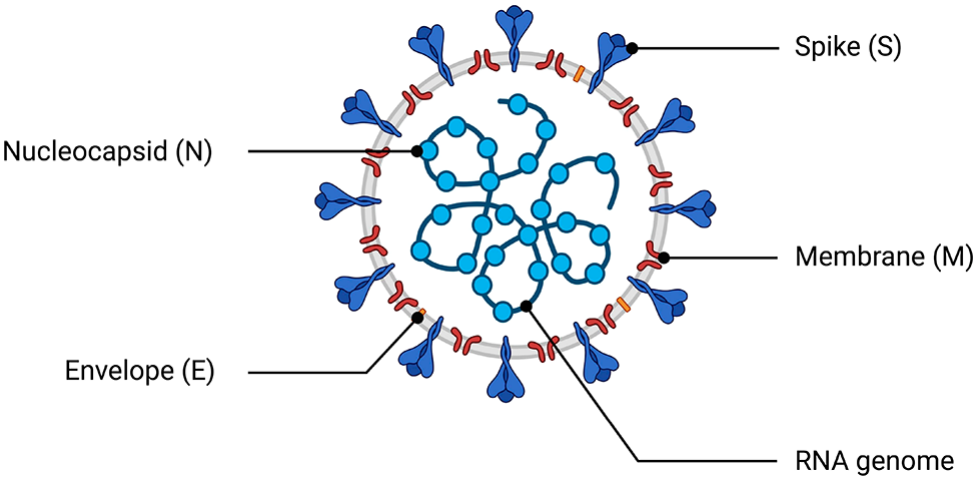

Severe acute respiratory syndrome coronavirus 2 (SARS-CoV-2) is a highly transmissible and virulent human-infecting coronavirus that emerged in late December 2019 in Wuhan, China, causing a respiratory disease called coronavirus disease 2019 (COVID-19), which has massively impacted global public health and caused widespread disruption to daily life. The crisis caused by COVID-19 has mobilized scientists and public health authorities across the world to rapidly improve our knowledge about this devastating disease, shedding light on its management and control, and spawned the development of new countermeasures. Here we provide an overview of the state of the art of knowledge gained in the last 2 years about the virus and COVID-19, including its origin and natural reservoir hosts, viral etiology, epidemiology, modes of transmission, clinical manifestations, pathophysiology, diagnosis, treatment, prevention, emerging variants, and vaccines, highlighting important differences from previously known highly pathogenic coronaviruses. We also discuss selected key discoveries from each topic and underline the gaps of knowledge for future investigations.

Coronaviruses (CoVs) are enveloped RNA viruses that belong to the *Coronaviridae* family within the order *Nidovirales*. They are a diverse group of enveloped positive-sense single-stranded RNA (+ssRNA) viruses that widely infect humans and animals and cause respiratory, hepatic, neurological, and enteric diseases.^1^ In humans, four coronaviruses (CoV-229E, CoV-OC43, CoV-NL63, and CoV-HKU1) are endemic and typically are associated with mild respiratory disease in healthy individuals.^2^ However, in the last two decades, three zoonotic coronaviruses originating from bats have emerged and caused severe respiratory disease in humans: severe acute respiratory syndrome coronavirus (SARS-CoV),^3,4^ Middle East respiratory syndrome coronavirus (MERS-CoV),^5^ and, most recently, the pandemic coronavirus named severe acute respiratory syndrome coronavirus 2 (SARS-CoV-2).^6−8^

SARS-CoV-2 was first reported in early December 2019 in Wuhan, Hubei Province, China, causing an outbreak of respiratory illness later named coronavirus disease 2019 (COVID-19).^8,9^ The rapid spread of the disease outside China led the World Health Organization (WHO) to declare a Public Health Emergency of International Concern (PHEIC) on January 30, 2020, and subsequently, a pandemic on March 11, 2020.^10^ As of July 14, 2022, more than 559.5 million cases of COVID-19 infection and 6.3 million deaths have been reported worldwide, most of which involved people living in the USA, followed by those living in India, Brazil, and France.^11^

SARS-CoV-2 shows sustained person-to-person transmission through direct contact and the air (as respiratory droplets and/or aerosols).^12,13^ The infection has a median incubation period of approximately 4–5 days, but it can be as long as 14 days.^14,15^ The most common symptoms reported in patients with COVID-19 are fever, fatigue, and dry cough.^14,16,17^ Other less common symptoms include headache, sore throat, myalgia, diarrhea, vomiting, chills, loss of smell, and loss of taste.^14,16−18^ Clinically, many COVID-19 patients present mild to moderate symptoms (81%). However, approximately 14% of infected patients progress to pneumonia and may require ventilation in an intensive care unit (ICU), and 5% eventually develop more critical manifestations such as acute respiratory distress syndrome (ARDS), septic shock, and multiple organ dysfunction or failure.^19−21^

Since the emergence of the virus and its subsequent spread across the world, rapid progress has been made toward understanding many features of COVID-19. Based on the current scientific knowledge, this comprehensive review outlines the latest information on many topics related COVID-19 generated over the last 2 years, including origin and natural reservoir hosts, viral etiology, epidemiology, routes of transmission, clinical manifestations, pathophysiology, diagnosis, treatment, prevention, emerging variants, and vaccines.

## Brief History

In the early 1930s, members of the *Coronaviridae* family were identified as responsible for infectious illness in several animal species, including mice, chickens, and pigs.^22^ In the 1960s, Tyrrell and other virologists visualized morphological features of mouse hepatitis virus, bronchitis virus, and swine gastroenteritis virus using electron microscopy.^23,24^ This novel group of viral agents were called coronaviruses (referring to the crownlike appearance similar to a solar corona), and later this designation was officially recognized.^24,25^ During the same decade, human coronaviruses, including OC43 and 229E, were first isolated from human patients displaying upper respiratory disease.^26,27^

For approximately 40 years, no additional coronaviruses capable of infecting humans had been described. Later, the coronaviruses NL63 and HKU1 were first identified in 2004 and 2005, respectively, and have been added to the spectrum of viruses that cause the common cold.^28,29^ These CoVs are considered to be of low pathogenicity, with infection typically resulting in mild or moderate symptoms in immunocompetent individuals.^30^ In addition to these four endemic CoVs, three highly pathogenic respiratory betacoronaviruses have emerged from bats and caused severe outbreaks in humans during the 21st century. In 2002, severe acute respiratory syndrome coronavirus (SARS-CoV) emerged in Guangdong Province, China, and caused a disease with the same name.^3,31^ The virus rapidly spread to more than 27 countries, resulting in more than 8000 human infections and 774 deaths between 2002 and 2004, with a lethality rate of approximately 10%.^4,32^ In 2012, a novel betacoronavirus called Middle East respiratory syndrome coronavirus (MERS-CoV) was first identified in a Saudi Arabian patient suffering from a severe respiratory disease that was later named Middle East respiratory syndrome (MERS).^5,33^ MERS-CoV has been reported in 27 countries to date, mainly in the Middle East region, resulting in more than 2500 laboratory-confirmed cases and 927 deaths, with a mortality rate of 35%.^34^ Whereas there have been no reported SARS cases anywhere in the world since late 2004,^35^ MERS-CoV is still actively circulating in the Middle East.^34^

Despite the concerns raised by SARS-CoV and MES-CoV, both viruses failed to establish efficient transmission in the human population. However, the third of the highly pathogenic emergent CoVs proved to be different. On December 31, 2019, health officials reported to the WHO China Country Office cases of pneumonia of unknown etiology detected in Wuhan City, Hubei Province, China. The cluster of cases was epidemiologically linked to the Wuhan Huanan Seafood Wholesale Market, a large public market that commercializes seafood and several species of domestic and wild animals. An epidemiological and etiological investigation was initiated, and highly pathogenic CoVs were suspected on the basis of the clinical presentation, the time of the year (winter), and the link of the patients with a wet market, which was similar to SARS infections. A series of patients were sampled and submitted to pan-CoV PCR testing. Five samples turned out be PCR-positive for CoVs, and metagenomics analysis of one sample using next-generation sequencing (NGS) identified a novel CoV, which was first called 2019-nCoV. Other researchers independently discovered SARS-CoV-2 at the same time from different patients, and most cases had been at the market in Wuhan, suggesting that the earliest documented COVID-19 cases were indeed linked to the market.^8,9^ The International Committee on Taxonomy of Viruses (ICTV) and the WHO officially named the virus as SARS-CoV-2 and the disease as COVID-19.^36^

## Origin and Natural Reservoir Hosts

Despite the passage of 2 years since the beginning of the pandemic, there still exists a great mystery about the origins of SARS-CoV-2. While there has been speculation that SARS-CoV-2 had been created in a laboratory, a comparative genomic study suggested that this was not the case and supported two scenarios for the origin of SARS-CoV-2: (i) natural selection in an animal reservoir before zoonotic spillover and (ii) natural selection in humans following zoonotic spillover.^37^ These results suggested that SARS-CoV-2 is not a pathogen purposely manipulated or constructed in the laboratory. Phylogenetic analysis showed that SARS-CoV-2 is clustered with SARS-related coronaviruses (SARSr-CoVs) and the SARS-CoV previously reported in bats, placing it in the subgenus *Sarbecovirus* and genus *Betacoronavirus*.^8,9^ SARS-CoV-2 shares 79% genome identity with SARS-CoV and 50% with MERS-CoV.^7,8^ Although the origin and direct ancestral virus of SARS-CoV-2 are yet to be discovered, RaTG13, a CoV detected in the horseshoe bat *Rhinolophus affinis* in Yunnan Province, China, has 96.2% genome similarity with SARS-CoV-2 and is the closest relative of SARS-CoV-2 identified to date.^7^ Notably, the high genetic similarity between SARS-CoV-2 and related bat CoVs likely represents more than two decades of evolution, suggesting that these bat CoVs are the most probable evolutionary progenitor of SARS-CoV-2, while other intermediate hosts might have played a crucial role in the process of transmission to humans.^38,39^ Recently, scientists have identified SARSr-CoVs in *Rhinolophus shameli* bats sampled in Cambodia in the year 2010. They showed that these viruses share 92.6% nucleotide identity with SARS-CoV-2 and are closely related to SARS-CoV-2 in most genomic regions, except for the spike (S) protein that binds to the ACE2 receptor in human cells.^40^

Three recent new studies have added evidence of the role of the Huanan Seafood Wholesale Market in Wuhan in the emergence of SARS-CoV-2. Gao and co-workers tested 1380 environmental and animal samples collected within the market in early 2020 and found 73 environmental samples positive for SARS-CoV-2, whereas none of the animal samples were positive. They were able to isolate live SARS-CoV-2 from three environmental samples. Worobey and co-workers used spatial analysis and genomic data to show that the earliest known COVID-19 cases were geographically and epidemiologically linked to the Huanan seafood market. Pekar and co-workers used Bayesian phylogenetic analysis in early SARS-CoV-2 sequences to show that the emergence of SARS-CoV-2 occurred *via* multiple zoonotic events in a similar way as SARS-CoV in 2002 and 2003. Together, these studies suggest that the market played a major role as the epicenter of SARS-CoV-2 emergence and further weakened the lab-leak hypothesis.^41−43^

**Figure 1:**
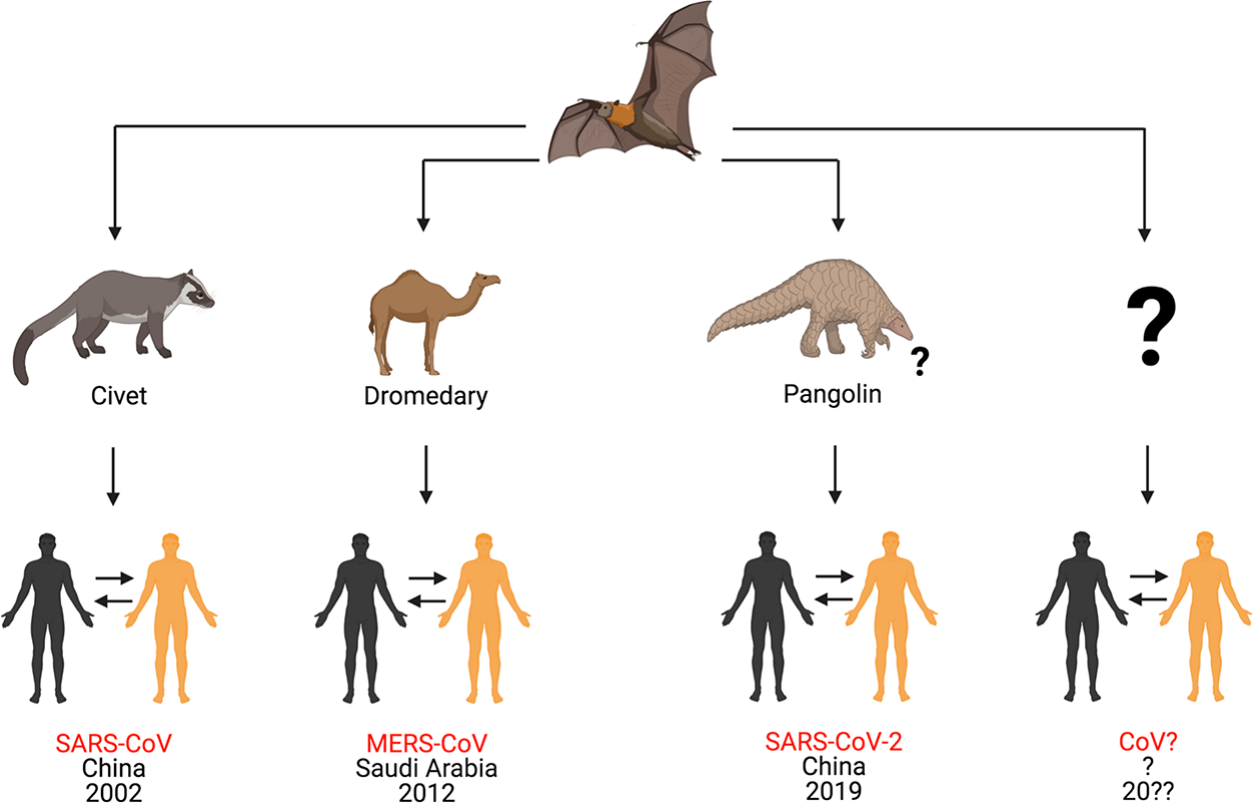
Origins of different coronaviruses. In the 21st century, three highly pathogenic betacoronaviruses have emerged from bats to cause respiratory disease in humans. In 2002, a betacoronavirus called severe acute respiratory syndrome coronavirus (SARS-CoV) emerged in Guangdong Province, China, and caused respiratory disease in humans. One decade later, another betacoronavirus called Middle East respiratory syndrome coronavirus (MERS-CoV) was reported in Saudi Arabia. Both SARS-CoV and MERS-CoV emerged from bats and were transmitted to humans *via* civets and dromedary camels, respectively. Later, in December 2019, a novel betacoronavirus called severe acute respiratory syndrome coronavirus 2 (SARS-CoV-2) emerged from bats and caused a pandemic disease called coronavirus disease 2019 (COVID-19). It was likely transmitted to humans by pangolins, although its origin is still being investigated. In summary, coronaviruses represent an example of emerging zoonotic viruses that have crossed the species barrier to cause disease in the human population. The possibility of a new, highly pathogenic coronavirus emerging from wild animals in the next few years cannot be ruled out. This figure was created with Biorender.com.

With regard to intermediate hosts, both SARS-CoV and MERS-CoV emerged from bats and were transmitted directly to humans from civets and dromedary camels, respectively.^2,6^ However, knowledge about the intermediate host(s) for SARS-CoV-2 remains incomplete and requires further studies (Figure 1).^7,44^ The identification of intermediate hosts is crucial for public health measures to prevent future outbreaks of SARS-CoV-2 or related viruses.^37,45,46^ Some studies have suggested that pangolins can host SARS-CoV-2.^47−50^ SARS-CoV-2-related viruses have been detected in and isolated from tissues of Malayan pangolins from China with clinical signs of disease and histological alterations.^50^ In that study, Xiao and colleagues revealed that a CoV isolated from the Malayan pangolin showed 100%, 98.6%, 97.8%, and 90.7% amino acid identity with SARS-CoV-2 envelope [E], membrane [M], nucleocapsid [N], and spike [S] proteins, respectively.^50^ Lam and co-workers used metagenomics and phylogenetic analysis to show that the viruses from pangolins were associated with two distinct sublineages of SARS-CoV-2-related coronaviruses, including one that exhibited strong similarity (97.4% amino acid similarity) in the receptor-binding domain (RBD) to SARS-CoV-2.^47^ Similarly, Liu and colleagues assembled the complete genome of a coronavirus identified in three sick Malayan pangolins and demonstrated that it was genetically related to SARS-CoV-2.^49^ Taken together, these results suggest that pangolins have the potential to act as an intermediate host of SARS-CoV-2, although more studies are needed to confirm this hypothesis.

Since the emergence of SARS-CoV-2, research groups from across the world have investigated the susceptibility of domestic animals to SARS-CoV-2 infection.^51−53^ In this context, Shi and co-workers provided and discussed important insights into the animal reservoirs of SARS-CoV-2.^53^ They investigated the susceptibility of ferrets and other animals to SARS-CoV-2 infection, most of them traditionally having close contact with humans, including cats, dogs, pigs, ducks, and chickens, and demonstrated that SARS-CoV-2 replicates effectively in cats and ferrets but poorly in dogs, pigs, ducks, and chickens.^53^ Additionally, it was demonstrated that SARS-CoV-2 can be transmitted easily among cats through respiratory droplets.^53^ Similarly, Halfmann and colleagues evaluated the transmission of SARS-CoV-2 in domestic cats and provided evidence of the potential human–cat–human transmission chain.^52^ In that study, none of the infected cats showed any clinical signs of disease, such as fever, substantial weight loss, or conjunctivitis, suggesting that cats might be a silent intermediate host for SARS-CoV-2. However, there is no clear evidence supporting the hypothesis that SARS-CoV-2 can be transmitted from infected animals to humans,^54^ and further studies are required to understand the role of cats and other domestic animals in the transmission of SARS-CoV-2 to humans.

In addition to domestic animals, many studies have been conducted to establish experimental animal models for SARS-CoV-2.^51,55^ The development and identification of animal models for studying SARS-CoV-2 are crucial for the study of virus biology, transmission, and COVID-19 pathogenesis and to evaluate potential therapeutic agents and vaccines.^55^ The susceptibility of many animal species, including hamsters, mice, ferrets, rabbits, bats, ducks, pigs, chickens, minks, and non-human primates, to SARS-CoV-2 infection has been investigated.^53,55−59^ In general, the results demonstrate that susceptibility varies according to animal species and that hamsters, human ACE2-transgenic mice, ferrets, and non-human primates seem to be more promising *in vivo* models. To date, our knowledge of the intermediate hosts of SARS-CoV-2 remains incomplete, and all reservoir hosts of the virus have not been clearly established. Therefore, experimental studies using animal models aiming to determine potential reservoir hosts should be addressed to elucidate other routes for the spread of SARS-CoV-2 within and among humans and animals.^51,60,61^

## Etiology and Replication Cycle

**Figure 2:**
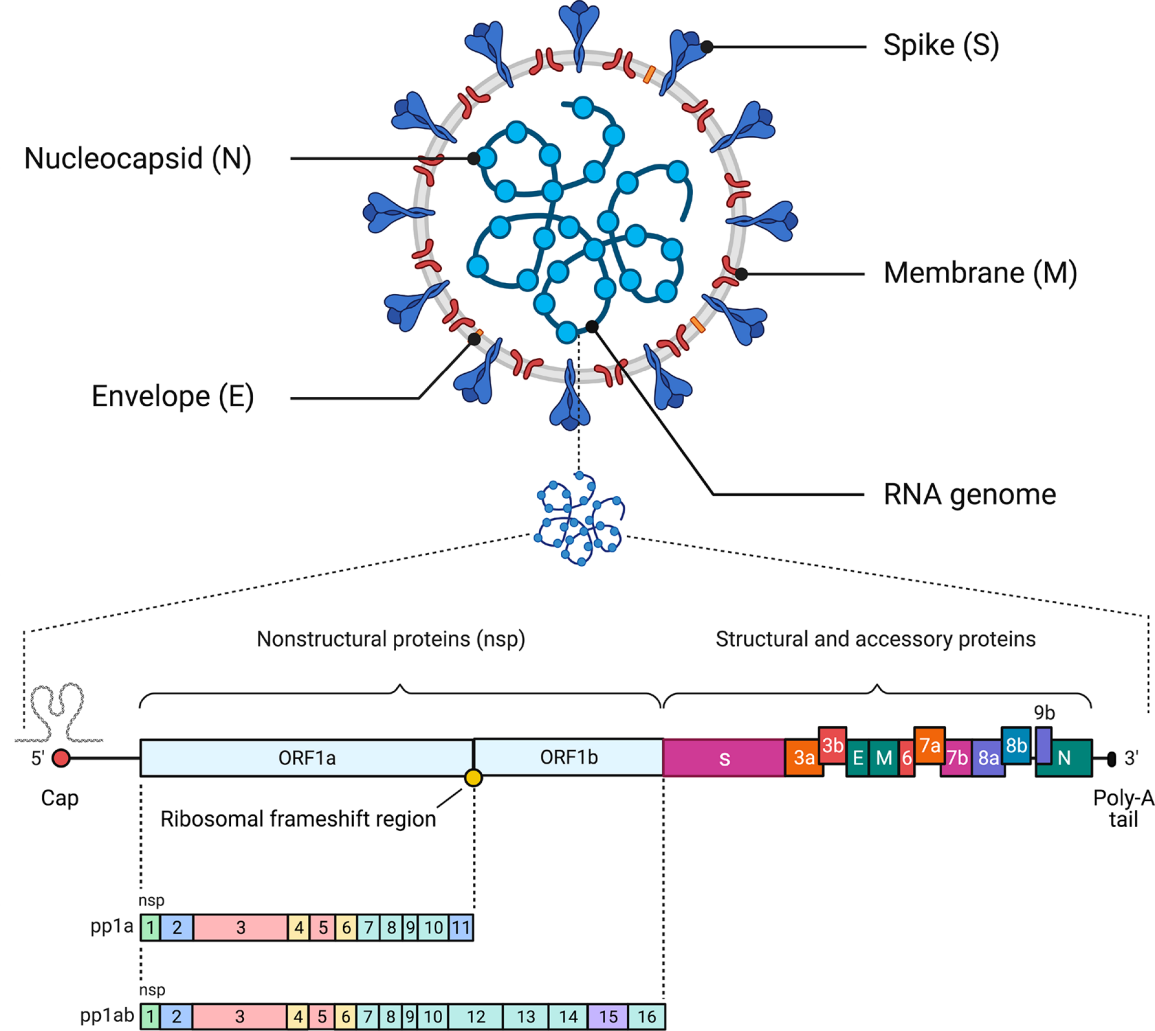
Schematic of the SARS-CoV-2 virus particle and genome architecture. The top panel illustrates the general structure of the SARS-CoV-2 viral particle, indicating its structural proteins and genome. The bottom panel illustrates the genome organization of SARS-CoV-2, including the 5′ cap, the region that encodes the nonstructural proteins required for viral replication (nsp1–nsp16), the region that encodes accessory and structural proteins (spike [S] protein, membrane [M] protein, envelope [E] protein, and nucleocapsid [N] proteins), and the poly-A tail. This figure was created with Biorender.com.

SARS-CoV-2 is a CoV member of order *Nidovirales*, family *Coronaviridae*, subfamily *Orthocoronavirinae*. This subfamily is subdivided into four genera on the basis of genetic characteristics: *Alphacoronavirus* (α-CoV), *Betacoronavirus* (β-CoV), *Gammacoronavirus* (γ-CoV), and *Deltacoronavirus* (δ-CoV).^62^ Similar to SARS-CoV and MERS-CoV, SARS-CoV-2 belongs to the β-CoV cluster and has a diameter of 80–160 nM and an RNA genome that is approximately 30 kilobases (kb) in length (Figure 2).^7,63−65^ All viruses in order *Nidovirales* are enveloped with a genome consisting of a single +ssRNA with a 5′-cap structure and a 3′-poly-A tail, allowing it to function as an mRNA for the replicase proteins.^62^ The ORF1a and ORF1b genes occupy two-thirds of the 5′ genome and are translated into polyprotein 1a (pp1a) and polyprotein 1ab (pp1ab), respectively.^66^ The resulting polyproteins 1a and 1ab are cleaved by the 3C-like protease (3CLpro) and the papain-like protease (PLpro). As a result of this process, pp1a is cleaved into 11 individual nonstructural proteins (nsps), and pp1ab is translated after a ribosomal frameshift takes place in the −1 position of the ORF1a stop codon and is then cleaved into 16 nsps (Table 1).^60,66^ The SARS-CoV-2 structural proteins (the spike [S], membrane [M], envelope [E], and nucleocapsid [N] proteins) are encoded by one-third of the viral genome, and these proteins are required for the assembly of new viral particles.^1,62,67^ The S protein encodes the signal peptide (SP), RBD, subdomain 1 (SD1), and subdomain 2 (SD2) in the S1 subunit and the fusion peptide (FP), heptad repeat 1 (HR1), heptad repeat 2 (HR2), and transmembrane (TM) in membrane-fusion subunit (S2).^68^ SARS-CoV-2 also encodes accessory proteins, including ORF3a, ORF3b, ORF6, ORF7a, ORF7b, ORF8a, ORF8b, and ORF9b, all of which are distributed among the structural genes.^38,69^ In a rapidly moving field of study, studies have suggested the presence of other accessory proteins (ORF3c, ORF3d, ORF9c, and ORF10).^70,71^ Additionally, the 5′ end contains an untranslated region (UTR), which forms multiple stem–loop structures needed for RNA transcription and replication.^62^

**Table 1:** Roles of Nonstructural Proteins (nsps) of SARS-CoV-2

nsp	functions^a^	refs
nsp1	disrupting the mRNA export machinery to inhibit host gene expression; inhibiting host protein translation; inhibiting IFN pathway	(633−636)
nsp2	linking viral transcription within the viral replication–transcription complex (RTC) to the initiation of translation	(637)
nsp3	essential component of the replication/transcription complex; also responsible for inhibiting host innate immune response, promoting cytokine expression, and cleaving viral polypeptides	(638−640)
nsp4	critical role in the organization and stability of DMVs	(641−643)
nsp5	3CL^pro^ and M^pro^ protease activity; blocking the IFN pathway	(644−646)
nsp6	organizing DMVs; restoring autophagosome expansion; involved in autophagy	(643), (647), (648)
nsp7	cofactor with nsp12; forming the hexadecameric complex with nsp8; exhibiting primer-independent RNA polymerase activity	(649−651)
nsp8	cofactor with nsp12; forming the hexadecameric complex with nsp7; primase	(649), (650), (652)
nsp9	RNA binding; enhances dimerization with diverse modes; interacts with nsp8; probably involved in viral RNA replication	(653−655)
nsp10	cofactor for the N7-guanine-methyltransferase/exoribonuclease activities (nsp14) and for the 2′-O-methyltransferase activity (nsp16)	(656−659)
nsp11	dispensable for viral replication in cultured cells; intrinsically disordered protein	(660), (661)
nsp12	replication enzyme (RNA-dependent RNA polymerase)	(649,662−664)
nsp13	RNA helicase activity; RNA 5′ triphosphatase; blocking the IFN pathway	(636), (665−667)
nsp14	exoribonuclease activity; N7-methyltransferase activity	(559), (668−671)
nsp15	viral endoribonuclease activity; evasion of dsRNA sensors	(672−674)
nsp16	2′-O-methyltransferase activity; regulating host immunity response; RNA cap formation	(657), (658), (675−678)

a Abbreviations: DMV, double-membrane vesicle; IFN, interferon; 3CL^pro^, 3C-like protease; M^pro^, main protease.

As previously mentioned, coronavirus particles are composed of four main structural proteins, among which the S protein has an essential role during the initial attachment, fusion, and entry of the viral particle into the host cell.^1,72−74^ Moreover, the S protein plays a critical role in determining transmission ability and host tropism, and it is also the major target for vaccines and therapeutic antibodies.^72,74−80^ Structural studies of the S protein have identified residues in the protein’s RBD that are essential for binding to the host cell receptor, the majority of which are highly conserved or share similar side-chain characteristics compared to the SARS-CoV RBD.^81,82^ Other motifs and domains of the S protein are key mediators of viral entry into host cells: the S1 subunit is used for binding to a host receptor and the S2 subunit for fusing the viral envelope and host cell membrane.^73^ Similar to other highly pathogenic viruses such as the avian influenza virus (AIV), the S protein of SARS-CoV-2 harbors a polybasic cleavage site (RRAR).^83^ This motif enables effective cleavage of S protein by furin and other proteases and is required for transmission of SARS-CoV-2.^84,85^ A growing body of data has demonstrated that furin cleavage of the SARS-CoV-2 spike protein promotes viral entry and allows cleavage during virus packaging.^84^ SARS-COV-2 interacts with the host protein angiotensin-converting enzyme II (ACE2), which is also a receptor for SARS-CoV, suggesting that these two CoVs share many steps in their replication cycles.^7,8,87−90^ SARS-CoV-2 and SARS-CoV have been shown to use the cell-surface transmembrane protease serine protease (TMPRSS2) for priming and entry, although other proteases such as cathepsin B (CatB) and CatL can also assist in this mechanism.^90^ The expression and tissue distribution of ACE2 consequently influence the tropism and pathogenicity of SARS-CoV-2.^91,92^ More recently, it was demonstrated that the SARS-CoV-2 S protein also binds to the surface receptor CD147 on the host cell, suggesting an alternative route to mediate the cell invasion of SARS-CoV-2.^93^ In contrast, MERS-CoV uses CD26 (also known as dipeptidyl peptidase 4, DPP4) as the cell receptor.^94^

**Figure 3:**
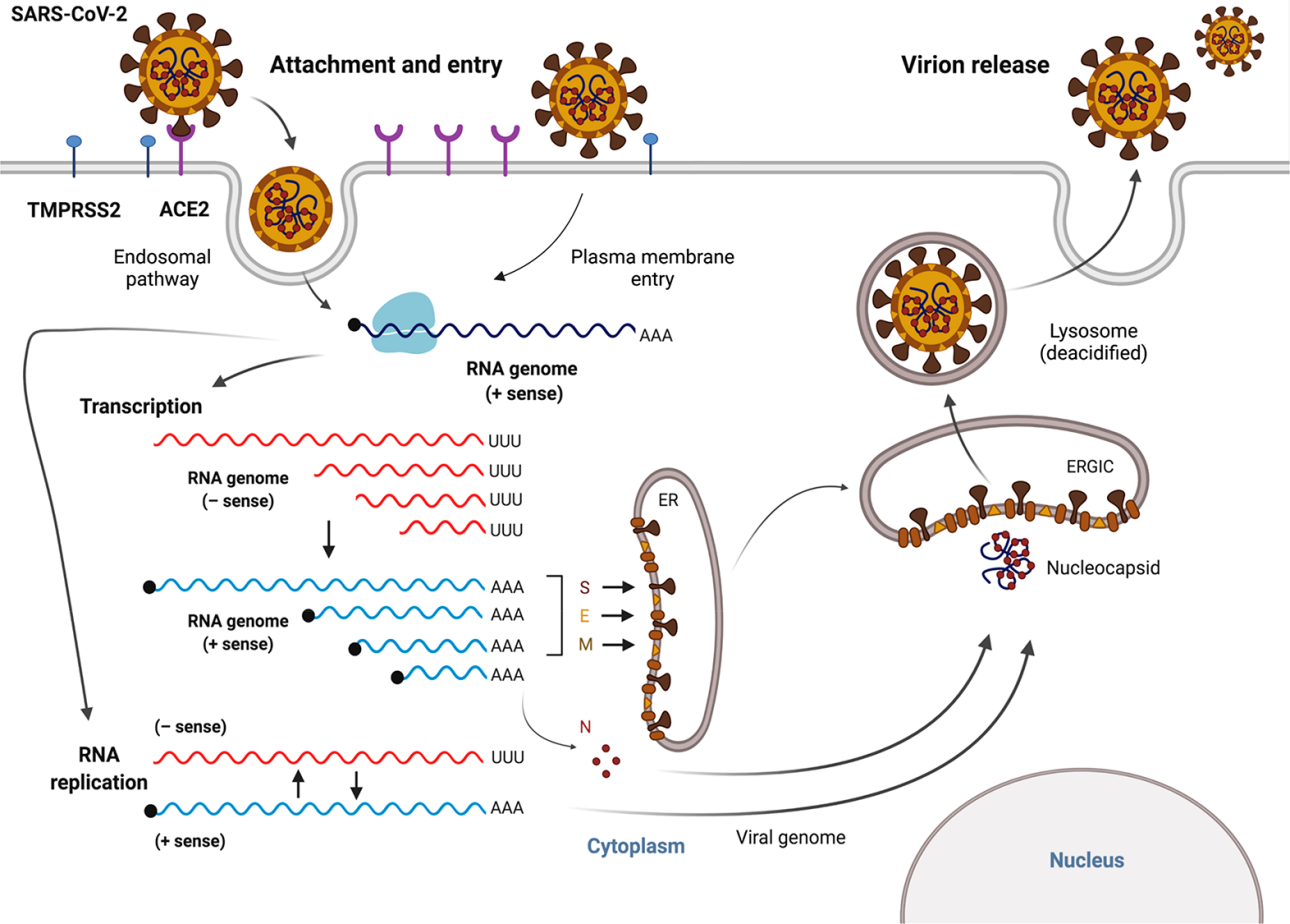
The SARS-CoV-2 replication cycle. SARS-CoV-2 enters the host cell *via* an endosomal pathway or through fusion of the viral envelope with the host cell membrane. Briefly, viral entry is initiated by binding of the RBD of the spike (S) protein to the human host cell receptor (ACE2). After the RBD–receptor interaction, the S protein undergoes proteolytic cleavage, which can be catalyzed by several host proteases, such as TMPRSS2, furin, and cathepsin B/L. Following viral entry, SARS-CoV-2 releases its genomic RNA into the cytoplasm and utilizes both the host’s and its own enzymatic machinery to replicate its genetic material and assemble new viral particles. The viral RNA genome is first translated into viral replicase polyproteins (pp1a and pp1ab), which are then cleaved into 16 nsps. In the process of genome replication and transcription mediated by the replication–transcription complex (RTC), the negative-sense (− sense) genomic RNA is synthesized and used as a template to generate a positive-sense (+ sense) genomic RNA and subgenomic RNAs. Viral assembly is aided by the interaction between viral genomic RNA and structural proteins located in the endoplasmic reticulum (ER) and ER–Golgi intermediate compartment (ERGIC). Finally, these virions are released to the plasma membrane *via* deacidified lysosomes and secreted from the infected cell *via* exocytosis. This figure was created with Biorender.com.

SARS-CoV-2 is highly transmissible and displays broad tissue tropism, which is determined by the susceptibility and permissiveness of specific host cells to the virus.^95^ During the infection process, the SARS-CoV-2 replication cycle starts with binding of the S protein to the host receptor ACE2, which together with host factors (*e.g.*, furin, TMPRSS2, and Cat B/L) results in conformational changes in the S protein followed by viral uptake.^85,91,96^ SARS-CoV-2 enters the host cell by direct fusion of the viral envelope protein with the host cell membrane or membrane fusion within the endosome after endocytosis.^97^ Next, viral replication takes place in the cytoplasm,^61^ where viral RNA utilizes the host and its own enzymatic machinery to replicate its genome, express viral proteins, and assemble new SARS-CoV-2 particles.^90,98^ More specifically, viral RNA is released into the host cytoplasm, and ORF1a and ORF1b are translated, after which the resulting products then go on to form the viral replication and transcription complex (RTC).^91^ In all coronaviruses, the translation of ORF1b requires a programmed −1 ribosomal frameshift, an alternative mechanism of translation to merge proteins encoded by two overlapping ORFs.^99^ After ribosomes reach the end of the ORF1a coding sequence, they encounter a frameshifting element that causes the ribosomes to backtrack by one nucleotide and reposition in the −1 reading frame before continuing the translation and producing a full-length ORF1ab polyprotein.^100^ As a result of the translation of nonstructural proteins, viral genomic RNA replication and transcription of subgenomic mRNAs (sg mRNAs) are initiated.^91^ These subgenomic RNAs are then transcribed and translated to produce accessory and structurally relevant proteins for the replication cycle and the production of new viral particles.^101^ Assembly of virions occurs *via* the interaction between viral genomic RNA and structural proteins located in the endoplasmic reticulum (ER) and the ER–Golgi intermediate compartment (ERGIC). Finally, these virions are released to the plasma membrane *via* deacidified lysosomes^102^ and secreted from the infected cell *via* exocytosis (Figure 3).^90,101^

## Epidemiology

Since the emergence of SARS-CoV-2 in China, the virus has rapidly spread worldwide and has shaken our health care and economic systems.^10^ Two years after the beginning of the COVID-19 pandemic, the virus remains a public health threat, although the number of cases and deaths has declined globally thanks mainly to the large scale deployment of effective vaccines.^11^ To date, the USA leads the number of laboratory-confirmed cases, followed by India, Brazil, and France. The global case fatality rate of COVID-19 is approximately 1.2%.^11^ A modeling study suggested that approximately 24% of fatal COVID-19 cases are underreported in the USA, representing more than 180 000 deaths, suggesting that almost a quarter of deaths attributable to COVID-19 are currently not reported as such on the death certificate.^103^ The Americas are currently responsible for almost half of all COVID-19 deaths throughout the world, followed by Europe, despite the fact that the latter reported higher total numbers of COVID-19 cases. A possible reason for this apparent disparity is test shortage in most Latin American countries, which may have underestimated the true incidence of COVID-19 (https://www.worldometers.info/coronavirus/). The observed differences may also be attributed to patient access to high-quality care. According to a study done in Brazil with the first 250 000 patients admitted to hospitals with COVID-19, 80% of the patients who needed invasive ventilation died, which is higher than the mortality reported for intubated patients in Europe (51.7% to 69%).^104^

**Figure 4:**
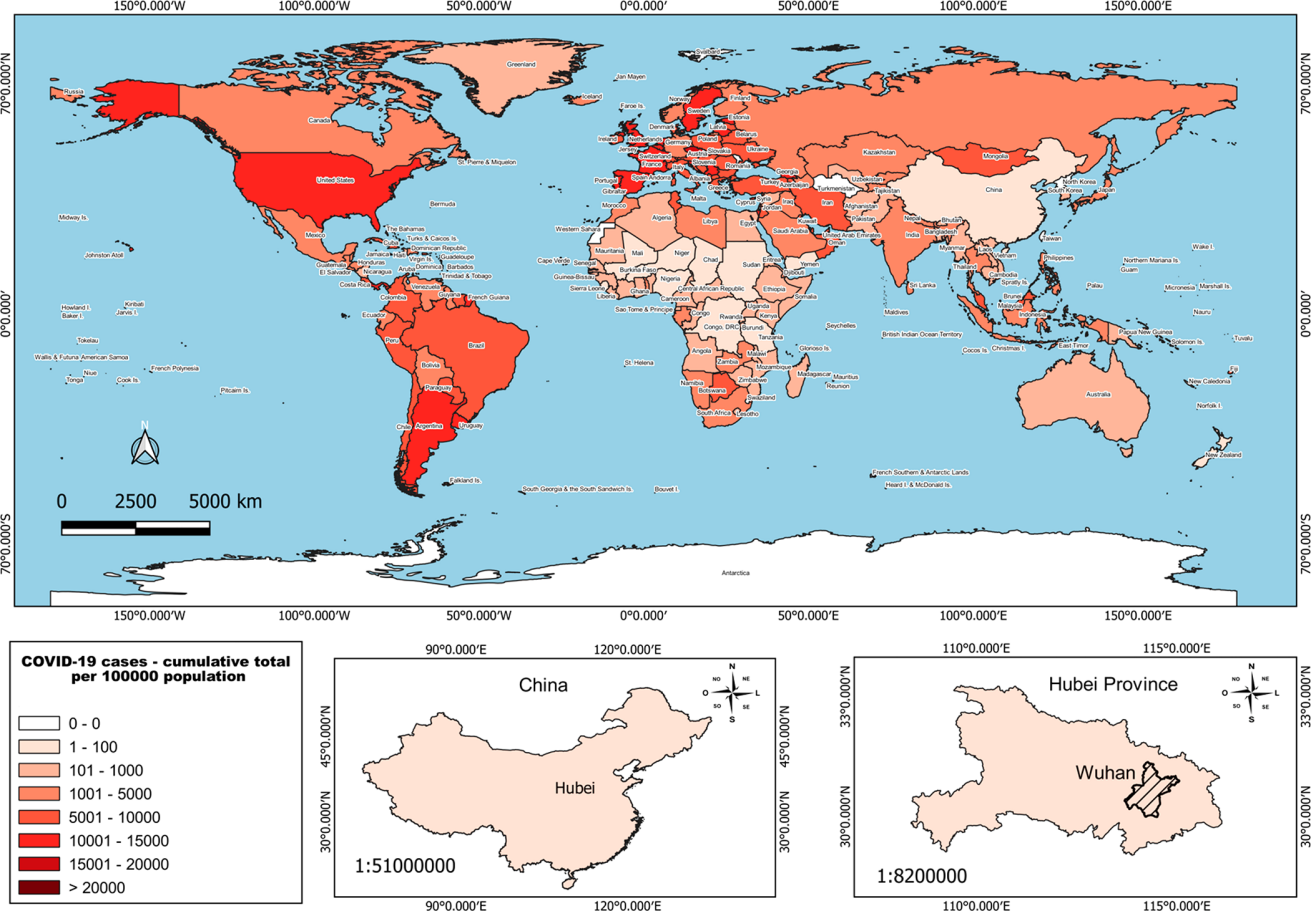
Epidemiology map of COVID-19. Cumulative cases of COVID-19 in all countries throughout the world. The bottom panels indicate the geographic location of Wuhan and Hubei Province in China, where the first COVID-19 cases were identified. The data were obtained from the World Health Organization (WHO).

Compared with the global trend, the currently least affected continent is Africa. The first case of COVID-19 in Africa was reported on February 14, 2020, and to date, the epidemic curve on the continent has remained stable compared with the Americas and Europe. The African continent reported 8 488 173 cases and 170 610 deaths from COVID-19 as of March 15, 2022 (https://covid19.who.int/table). Attempts have been made to explain the low rate of COVID-19 morbidity and mortality in Africa, and possible associated factors have emerged, such as population demography, climate, urbanization, and economic level.^105^ Compounding the challenge of such studies, the impact of the pandemic appears to be poorly characterized in low- and middle-income countries. Insufficient diagnostic capabilities and inadequate infrastructure have limited the availability of robust data, resulting in uncertainty about the status of the pandemic.^106^Figure 4 shows the cumulative cases of COVID-19 in all countries across the world according to data from the WHO.

The transmissibility of a virus is indicated by the reproduction number (*R*_0_), which represents the average number of new infections generated by an infected person throughout their infectious period in a totally naive population. Therefore, for *R*_0_ < 1 the number of infections declines or remains constant, whereas for *R*_0_ > 1 the number of infections is likely to increase. SARS-CoV-2 studies have suggested that an exponential increase of SARS-CoV-2 infection occurs when *R*_0_ ranges from 1.4 to 6.49 with an average of 3.28,^107,108^ which corresponds to each infected person transmitting the virus to over three individuals.^107^ However, further studies are required to better understand the epidemiology and ability of SARS-CoV-2 to spread in the human population, especially after the deployment of effective vaccines and the emergence of more transmissible variants of SARS-CoV-2.

## Modes of Transmission

**Figure 5:**
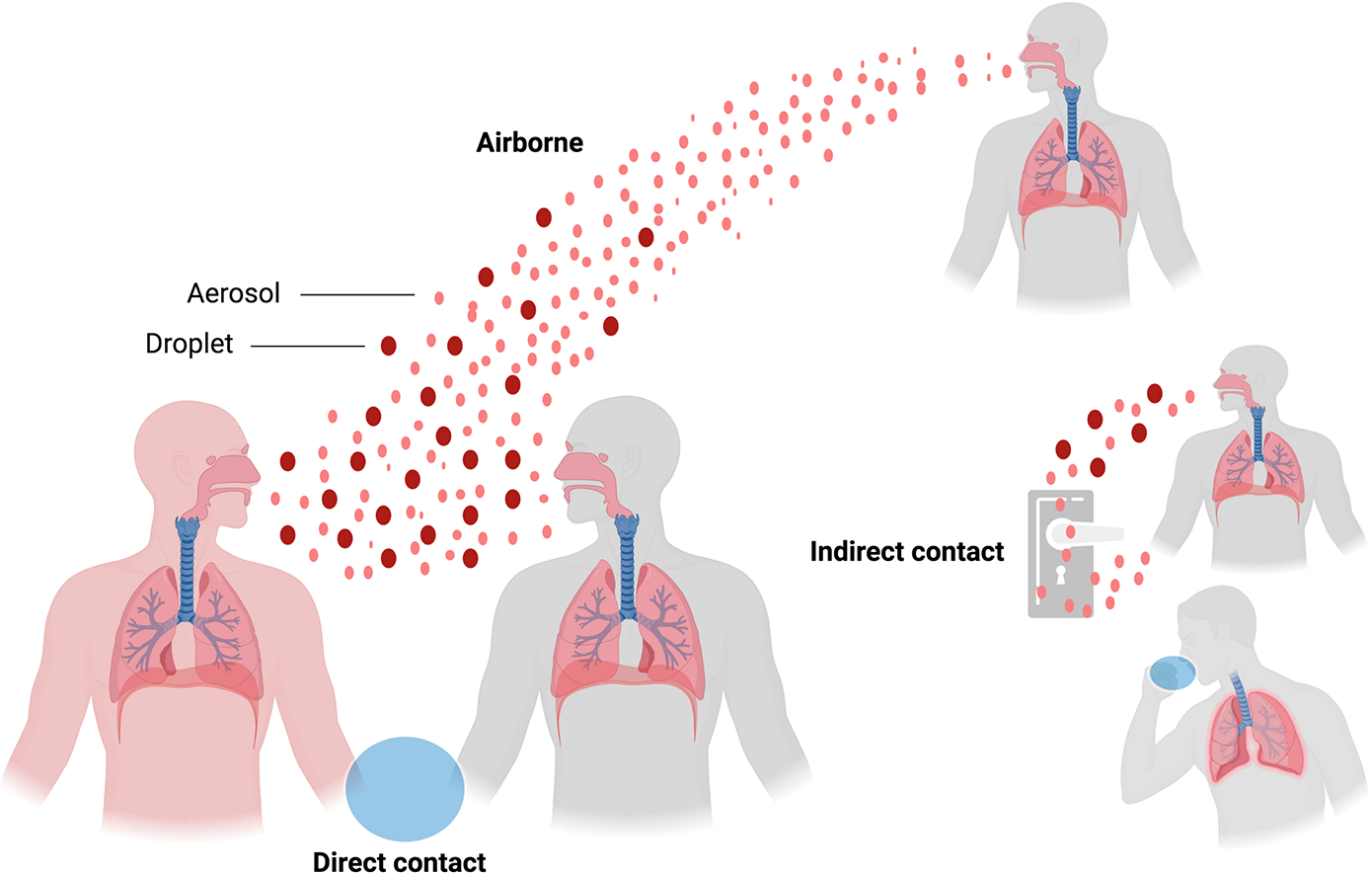
Major modes of SARS-CoV-2 human-to-human transmission. Transmission can be through direct contact of airborne infectious particles deposited in respiratory droplets and aerosols. Indirect contact by infectious particles deposited on fomites represents another potential route for viral transmission. This figure was created with Biorender.com.

According to current evidence, SARS-CoV-2 is transmitted from person to person when the infectious particles are released from the respiratory tract of an infected individual and reach the respiratory tract of a susceptible individual.^12,109^ Briefly, SARS-CoV-2 can be transmitted through three main routes that are not mutually exclusive: (i) airborne transmission (respiratory droplets and aerosols), (ii) direct contact (infectious virus deposited on persons), and (iii) indirect contact (infectious virus deposited on fomites) (Figure 5).^110^ Notably, SARS-CoV-2 has a high human-to-human transmission rate through close contact with infected persons,^111^ especially when the infectious virus is expelled during talking, breathing, coughing, or sneezing by an infected individual.^112−114^ SARS-CoV-2 enters the body through the mucous membranes of the eyes, mouth, or nose and spreads to the sinus cavity, throat, and nose lining until deposition along the human respiratory tract.^115^ After infection, the viral load in the upper respiratory tract appears to peak together with symptom onset, and viral shedding starts nearly 2 to 3 days before symptoms begin.^116^ Epidemiological and modeling studies have shown that transmission of SARS-CoV-2 may occur from symptomatic, asymptomatic, and presymptomatic persons,^117−121^ which suggests that the identification and isolation of individuals with symptomatic COVID-19 alone will not control the ongoing spread of SARS-CoV-2.^117^

Airborne and direct contact are considered the dominant routes for spreading of SARS-CoV-2 among persons.^12,13,122−124^ Prolonged exposure to any infected individual (6 foot proximity for at least 15 min) and short exposures to symptomatic (*e.g.*, coughing) infected persons are associated with successful virus transmission.^125^

SARS-CoV-2 transmission may also occur *via* contact with contaminated surfaces.^109,126^ Notably, the risk of infection through this route is probably multifactorial and is influenced by the distance from the viral source, the amount of virus to which an individual is exposed, and the length of time since the virus has been deposited on the surface. The viability of SARS-CoV-2 on surfaces over time is affected by several environmental stressors, including humidity, temperature, and level of ultraviolet radiation.^12,127,128^ Previous studies conducted under laboratory conditions have shown the ability of SARS-CoV-2 to remain infectious on various surfaces (*e.g.*, paper, glass, and stainless steel) for up to 28 days at 20° C depending on the type of material^129^ and in aerosols for up to 3 h.^128^ To address this question in real-life settings, many recent studies have investigated the presence of SARS-CoV-2 contamination in the air and on environmental surfaces, including health care units^123,124,130−132^ and urban settings.^126,133−135^ The results demonstrate different levels of viral contamination varying from high^130,136^ to low^132^ or even no contamination by SARS-CoV-2 RNA.^134^ Additionally, most of the reported positive environmental samples were found to have high reverse transcription quantitative polymerase chain reaction (RT-qPCR) cycle threshold (Ct) values (>30) for most of the positive samples,^130,136^ indicating low viral load and the labile nature of SARS-CoV-2 in the environment. Importantly, the majority of these studies did not investigate the capacity of SARS-CoV-2 to be cultured from an environmental surface sample, which is crucial for understanding the role of SARS-CoV-2 RNA-positive samples in terms of infectious potential toward the human population.^130,132,136^ Taken together, recent aggregated studies reinforce the potential of environmental samples for SARS-CoV-2 transmission (*i.e.*, indirect contact), although virus spread *via* close contact remains the primary route for SARS-CoV-2 transmission.

Other possible routes of transmission are being evaluated by research groups around the world, including the fecal–oral and blood-borne routes and vertical transmission from mothers to neonates. SARS-CoV-2 RNA has been detected in stool samples of COVID-19 patients, suggesting that viral shedding in the stool could be a potential route of fecal–oral transmission.^137,138^ In addition, SARS-CoV-2 has also been reported in blood samples, but the risk of blood-borne transmission was shown to be negligible.^138,139^ With regard to vertical transmission, several meta-analysis studies based on the current scientific evidence have suggested a low risk of such transmission for the spread of SARS-CoV-2.^140−142^ There is no evidence that the infection may lead to vertical transmission of SARS-CoV-2 or serious adverse outcomes in newborns.^143−145^

## Clinical Manifestations

**Figure 6:**
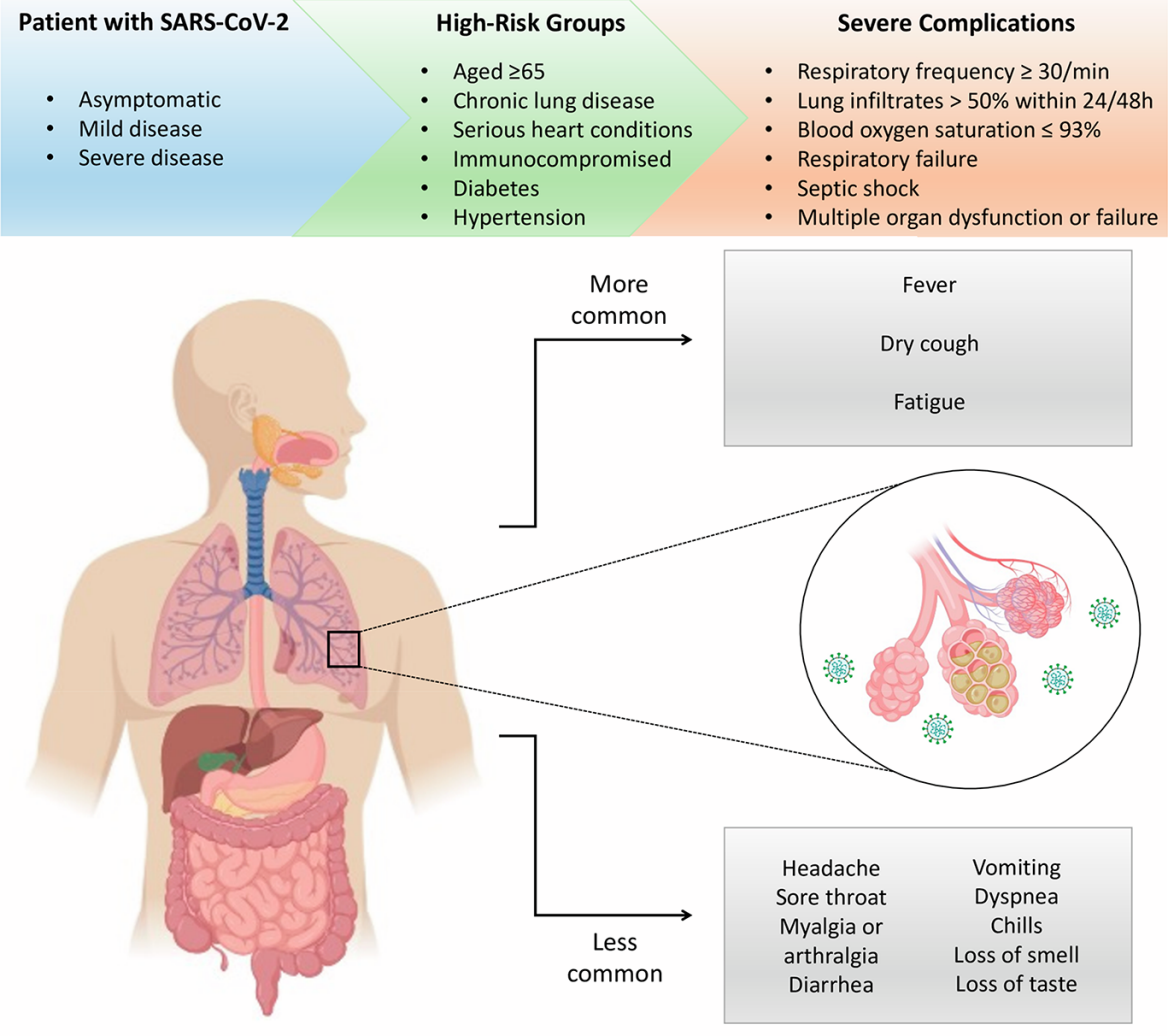
Clinical manifestations of COVID-19. Patients infected with SARS-CoV-2 can be asymptomatic, develop mild disease with diverse symptoms, or progress to severe illness. COVID-19 cases with severe complications are more frequently presented by patients from the high-risk group. This figure was created with Biorender.com.

The mean incubation period (the time of exposure to symptom onset) of COVID-19 is approximately 5 days (95% confidence interval [CI], 4.1–7.0 days) and, when it occurs, pneumonia within a median time of 8 days from disease onset.^15,20^ Approximately 97% of infected persons who develop clinical manifestations will do so within 11 days of infection.^146^ The median interval from symptom onset to hospital admission is 7 days (3–9 days).^147^ Recent studies have demonstrated that people of all ages are susceptible to SARS-CoV-2 infection, although the median age of infection is around 50 years.^14,16−18,20^ Overall, men ≥65 years old with comorbidities are more likely to be susceptible to develop a severe respiratory illness that requires hospital admission, while most young people and children experience asymptomatic infection or mild disease (Figure 6).^14,18^

The initial clinical presentations of SARS-CoV-2 infection are varied and often similar to symptoms caused by other respiratory viruses such as influenza and parainfluenza viruses, therefore representing a challenge for clinical diagnosis.^148^ The most common symptoms of SARS-CoV-2 infection are fever, dry cough, and fatigue.^14,16−18,149^ Less common symptoms include headache, sore throat, myalgia or arthralgia, shortness of breath, diarrhea, vomiting, dyspnea, chills, and alterations in smell (anosmia, hyposmia) and taste (ageusia, dysgeusia).^14,16−18,150^ In a recent study of 417 mild-to-moderate European COVID-19 patients, 85.6% and 88.0% reported olfactory and gustatory disorders, respectively.^151^ It was demonstrated that these dysfunctions persisted after the resolution of other symptoms and that women were significantly more affected by olfactory and gustatory dysfunctions than men,^151^ although the prevalence of these disorders may occur with varying intensities and should be considered as part of the clinical presentations of COVID-19.^152^ A meta-analysis showed that anosmia or hyposmia is significantly associated with positive COVID-19 infections.^153^ Other atypical presentations of COVID-19 include cutaneous manifestations, where individuals can present different types of lesions such as urticarial, livedoid, purpuric, maculopapular, thromboticischemic, and papulovesicular.^154−157^

According to current evidence, COVID-19 is recognized as a multiorgan disease with a broad spectrum of clinical presentations.^158,159^ In a large study including 72 314 individuals with COVID-19 in China, 81% of the cases presented mild or moderate symptoms, 14% of infected patients eventually developed severe pneumonia that required ventilation in an ICU, and approximately 5% of the cases had critical manifestations, which included patients with respiratory failure, septic shock, and/or multiple organ dysfunction or failure.^20,21^ Like post-acute viral syndromes reported in survivors of other pathogenic coronaviruses, there are increasing reports of persistent and prolonged effects after acute COVID-19, which are characterized by persistent symptoms and/or delayed or long-term complications beyond 3–4 weeks from the onset of symptoms.^159−161^ Moreover, studies have suggested that COVID-19 patients can develop a chronic disease or post-COVID-19 syndrome, which includes symptoms and abnormalities persisting beyond 12 weeks of the disease onset.^161−163^ COVID-19 complications in patients with severe disease may include impaired function of the lung, liver, heart, brain, coagulation system, and kidney.^158,159^ Risk factors for the development of severe COVID-19 include age ≥65 years and comorbidities such as hypertension, diabetes, chronic pulmonary disease, chronic kidney disease, immunodeficiencies, chronic liver disease, cancer, cardiovascular disease, and obesity.^164−169^ Typical characteristics of patients with severe COVID-19 include respiratory frequency ≥ 30/min, lung infiltrates > 50% within 20/48 h, blood oxygen saturation < 93%, and an altered Pa_O_2__/Fi_O_2__ ratio, which is associated with high mortality and morbidity.^19,20^

COVID-19 is usually a mild disease in children, including infants. When infected, most children remain asymptomatic.^170^ However, a small proportion (4%) of children with COVID-19 develop severe disease requiring ICU admission and prolonged ventilation, although fatal outcomes are overall rare.^171^ Approximately 2–5% of infected patients with COVID-19 are younger than 18 years, with a median age of 11 years.^171^ The underlying mechanisms of the severity of COVID-19 in children are being rapidly unraveled.^172^ The presence of comorbidities, immunological response, and genetic factors have been investigated to understand the spectrum of disease in children and adults.^172^ As the pandemic progressed, a cumulative body of data reported a multisystem inflammatory syndrome in children (MIS-C) due to SARS-CoV-2 infection.^173−176^ The pathogenesis of this rare MIS-C is still unclear but shares some characteristics with Kawasaki disease, suggesting a vascular and likely autoimmune etiology.^174^ A recent meta-analysis study evaluated 783 cases of MIS-C between March and June 2020.^177^ The results revealed that patients with MIS-C have a high frequency of gastrointestinal symptoms (71%), including abdominal pain (34%) and diarrhea (27%). Other common symptoms include cough and respiratory distress, which were found in 4.5% and 9.6% of the cases, respectively.^177^ While exhibiting a low lethality rate (1.5%), MIS-C appears to be a condition of higher severity for infected patients.^177^

## Pathophysiology

**Figure 7:**
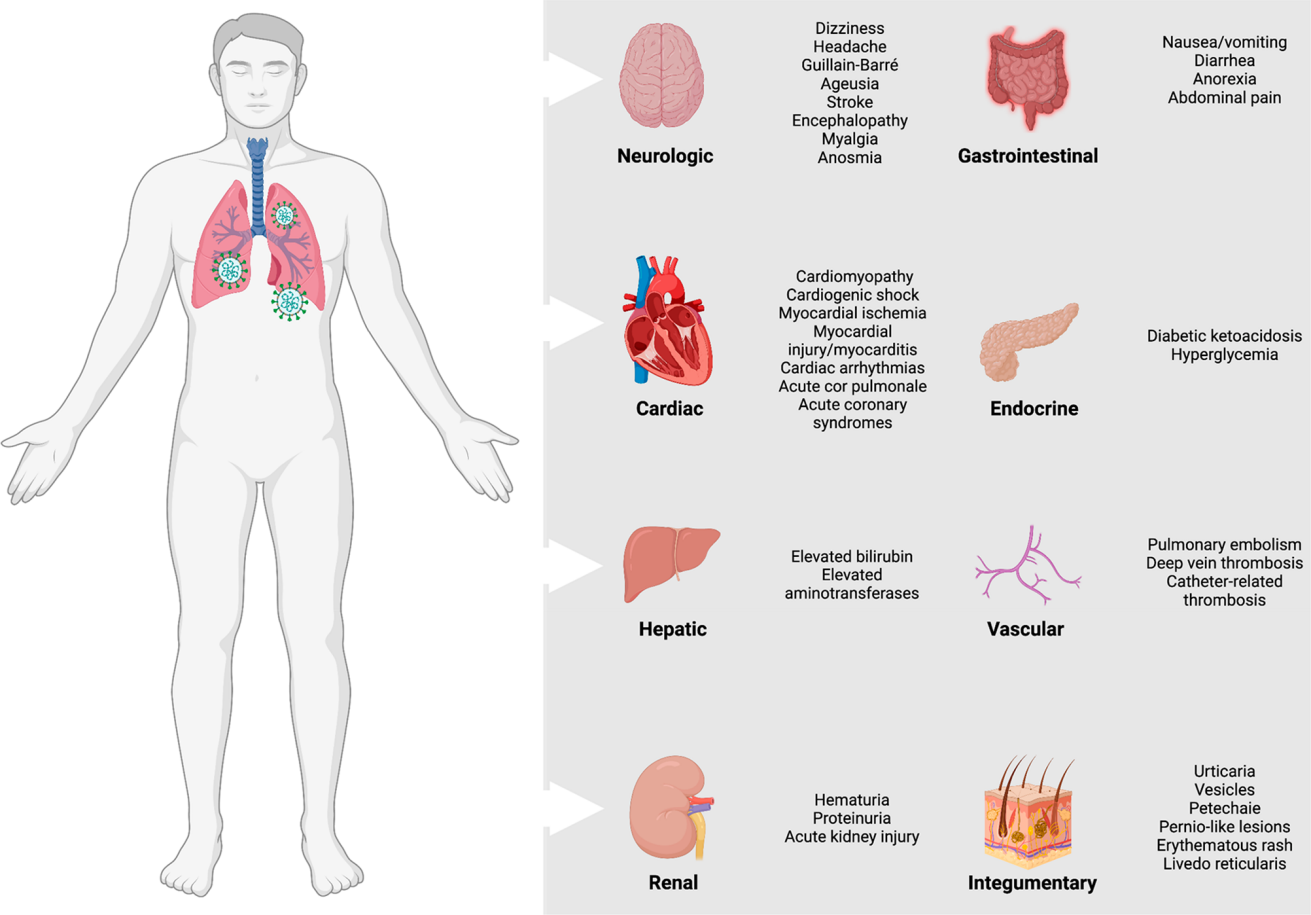
Extrapulmonary complications from COVID-19. The extrapulmonary complications include a wide spectrum of disorders in several systems, including the neurological, cardiac, hepatic, renal, gastrointestinal, endocrine, vascular, and integumentary systems, which may occur in severe and critically ill COVID-19 patients and are linked to prolonged hospitalization and increased mortality risk.^178,679^ This figure was created with Biorender.com.

While SARS-CoV-2 infection is known to cause substantial pulmonary disease, including pneumonia and ARDS in most patients, extrapulmonary manifestations of the disease are also a quite common feature, especially in severe cases.^178^ These include associated complications in several systems, including the neurological, cardiac, hepatic, renal, gastrointestinal, endocrine, vascular, and integumentary systems (Figure 7).^178,179^ In short, there are key factors that may have critical roles in the pathophysiology of multiorgan injury secondary to infection with SARS-CoV-2. These mechanisms include endothelial cell damage, thromboinflammation, dysregulation of the renin–angiotensin–aldosterone system (RAAS), dysregulation of the immune response, and direct viral toxicity.^178^

**Figure 8:**
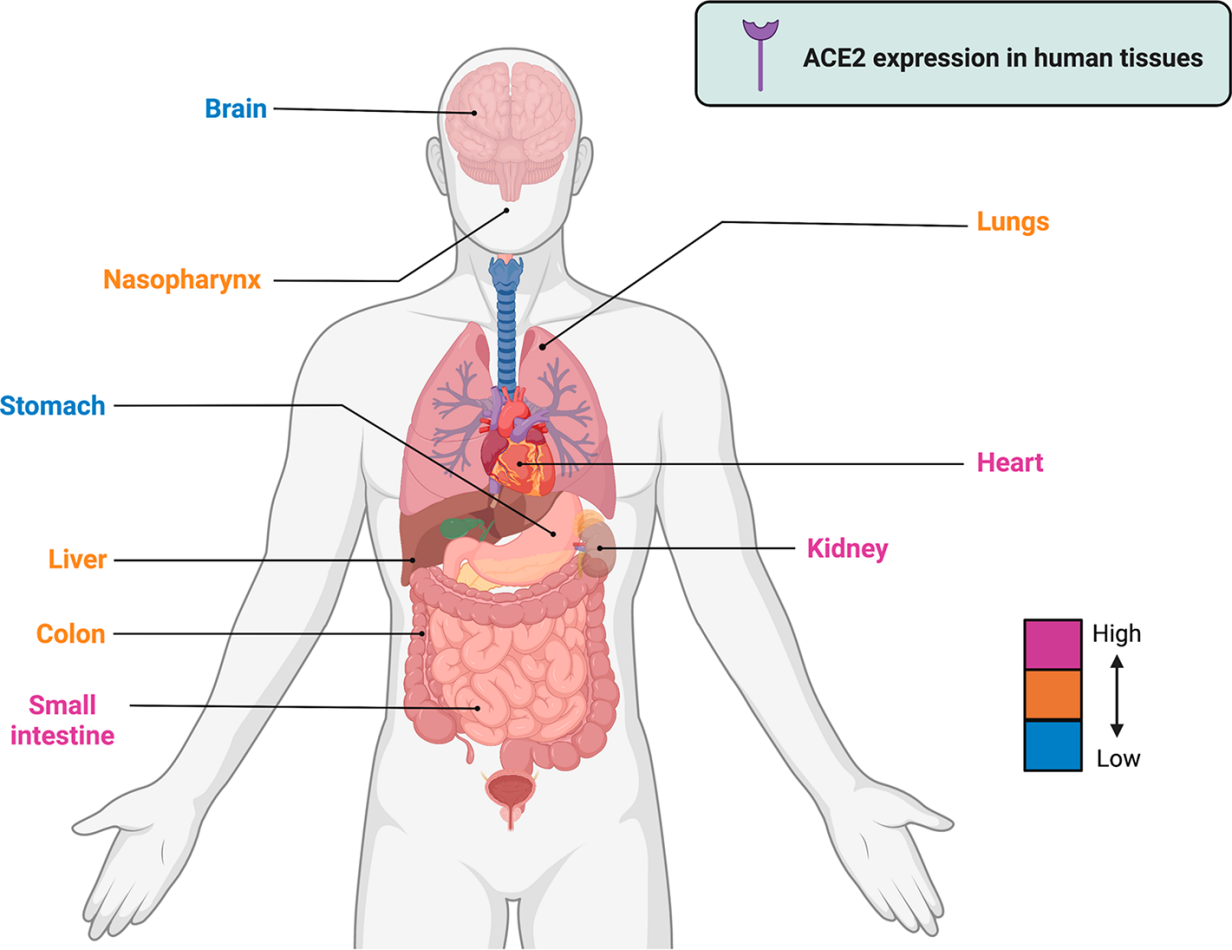
Gene expression of the ACE2 receptor in human tissues. The level of expression in each organ is categorized from high to low using different colors. Sources: https://www.proteinatlas.org/ENSG00000130234-ACE2/tissue and Li *et al.* (2020).^183^ This figure was created with Biorender.com.

SARS-CoV-2 attachment and entry into alveolar epithelial cells are dependent on the presence of ACE2, a strategy that is shared with SARS-CoV.^7,180^ However, the affinity of SARS-CoV-2 S protein to human ACE2 is around 10- to 20-fold higher than that of the SARS-CoV S protein,^181^ which explains the higher transmissibility of the novel coronavirus.^182^ ACE2 is highly expressed in several human tissues, such as the small intestine, heart, kidneys, testis, thyroid, and adipose tissue, and is expressed at low levels in the brain, blood, bone marrow, spleen, muscle, and blood vessels. ACE2 is expressed at moderate levels in the lungs, liver, bladder, colon, and adrenal glands (https://www.proteinatlas.org/ENSG00000130234-ACE2/tissue) (Figure 8),^183^ which could explain the tropism of SARS-CoV-2 and the wide spectrum of clinical pulmonary and extrapulmonary manifestations associated with COVID-19 disease.

SARS-CoV-2 requires proteolytic processing of the S protein to promote viral entry. Recent studies have demonstrated that proteases, including TMPRSS2, furin, and CatB/L, participate in cleavage of the S protein, resulting in entry of SARS-CoV-2 and fusion of the viral envelope and endosome.^86,90,184^ More recently, it has been suggested that SARS-CoV-2 can bind to another surface receptor, CD147, which would provide an additional route for host cell invasion (CD147–S protein).^93^ It is presumed that primary viral replication takes place in the mucosal epithelium of the upper respiratory tract, followed by multiplication in the lower respiratory tract.^185^ During viral entry into cells, pattern recognition receptors (PRRs), such as the endosomal toll-like receptors (TLR), can detect viral genomic RNA, which triggers an inflammatory response.^186^ Activation of TLR-3 or TLR-7 triggers a signaling pathway that releases the main transcriptional regulator of inflammation, NF-κB, from its inhibitor.^187^ Recognition of the S protein by TLR-2 placed on the cell surface can also contribute to signaling that activates the host defense response.^187^ Despite being a part of the host defense system, overactivation of TLR and its adaptor protein MyD88 has been hypothesized as a predisposition factor for exacerbated inflammation observed in COVID-19 patients with obesity.^188^ Once released, NF-κB migrates to the nucleus and activates genes that codify proteins with immunological properties, such as cytokines, chemokines, and other immunological mediators.^189^

Thereafter, fusion between the viral envelope and endosomal membrane induces release of the viral genome into the cell, which can be identified by other PRRs in the cytosol like MDA-5 or RIG-1.^190,191^ These PRRs will also trigger the activation of NF-κB, although through a different signaling pathway,^191^ and activate transcriptional regulators including IRF-3 and IRF-7 that induce the expression of the class I interferon (IFN) genes.^191^ An efficient antiviral response relies on the production of the class I IFNs IFN-α and IFN-β.^192,193^ The IFN family of proteins play key roles in host immunological response against viruses and other pathogens.^194^ Briefly, canonical type I IFN signaling activates the Janus kinase (JAK)–signal transducer and activator of transcription (STAT) pathway, which leads to the transcription of IFN-stimulated genes (ISGs) that confer antiviral activities to host cells.^194−198^ In the context of SARS-CoV-2 infections, a cumulative body of data has demonstrated that severe COVID-19 cases are associated with the presence of auto-antibodies that block the action of specific IFNs,^199,200^ a reduction of IFN signaling,^201,202^ and genetic variants that impair IFN signaling.^203,204^*In vivo* studies suggested type I IFN signaling as a driver of pathology in mouse models for SARS-CoV and SARS-CoV-2 infections.^205^ In a rapidly moving field of study, recent reports suggested that type I and III IFNs are linked to a disruption of lung barrier function and increased susceptibility to secondary bacterial infections in mice.^206,207^ Additionally, type II IFN, also known as IFN-γ, is transmitted through a different receptor, has effects that are independent of type I IFNs, and also plays a relevant role in combating viral infection and modulating the antiviral immune response.^208^ In COVID-19 patients with moderate and severe infection, IFN-γ was documented as an independent risk factor associated with mortality.^209^ Taken together, these findings and results reported by others have revealed multiple roles of IFN signaling during the clinical course of COVID-19, meaning that it is possible to observe context-dependent variations and that IFN signaling may attenuate or exacerbate COVID-19 pathology.^210^

In the absence of a proper antiviral response mediated by IFNs, the host will rely on other innate immune mechanisms for defense, like the immune cells.^211^ Activation of NF-κB leads to the production of cytokines and chemokines that will recruit and activate immune cells from the bloodstream.^198,212^ The first immune cells to reach the infection site from the circulation are neutrophils and monocytes.^213^ Several chemokines, such as CCL2, CCL3, and CXCL10, together with CCL7, are potent chemokines for monocytes and have been found at high concentrations in COVID-19 patients with severe disease.^211^ Abnormal levels of monocyte population subsets have been demonstrated in COVID-19 patients, suggesting the migration of intermediate (CD14^++^ CD16^+^) and nonclassical monocytes (CD14^+^ CD16^+++^) to inflamed tissue.^214−216^ Nonclassical monocytes have previously been associated with an immune response against viral infection.^216^ Normal or nearly normal levels of those monocyte subsets have been associated with moderate illness, and this has been suggested as a favorable prognostic indicator.^214,216^

More recently, Chevrier and colleagues described important insights about the immune signatures involved during the progression from mild to severe COVID-19 disease.^217^ The authors used mass cytometry and targeted proteomics to profile the innate immune responses of patients with mild or severe COVID-19 and healthy individuals. The results showed that the production of CD169+ monocytes, combined with IFN-γ+ MCP-2+ monocytes, rapidly follows symptom onset. At later stages, they found a persistent inflammatory phenotype in patients with severe COVID-19, dominated by high CCL3 and CCL4 cytokine abundance correlated with the reappearance of CD16+ monocytes, while the response of mild COVID-19 patients was normalized.^217^

Macrophages and monocytes are also cell types that are susceptible to SARS-CoV-2 infection, which triggers the production and secretion of inflammatory cytokines.^218−220^ Increased levels of several cytokines have been reported in patients with severe COVID-19, including interleukin (IL)-1β, IL-2, IL-6, IL-7, IL-8, IL-10, IL-17, IFN-γ, IFN-γ-inducible protein 10, MCP-1, G-CSF, MIP-1α, and TNF-α.^213^ The term cytokine storm is used to define this pathological overproduction of cytokines that leads to systemic inflammatory response affecting several organs, such as the heart, liver, and kidney, and is the leading cause of death in COVID-19 patients.^17,221,222^ High levels of proinflammatory cytokines in the circulation trigger several symptoms, including fever, headache, rash, diarrhea, arthralgia, and myalgia.^221^ A high level of cytokines in the circulation can lead to hypotension, vascular leakage, disseminated intravascular coagulation, and multiorgan failure.^223^ Many of these symptoms were reported in critical patients with COVID-19, and cytokine storm has been associated as part of the pathology in severe cases,^224^ although the mechanism that triggers this exacerbated immunological response has not been completely characterized. Although antibody-dependent enhancement (ADE) of infection plays a critical role in the pathogenesis of many viral infections, antibodies induced by SARS-CoV-2 infection do not contribute to inducing aberrant cytokine production by macrophages.^225^ Recent findings have shown that SARS-CoV-2 infection in lung-resident human macrophages is a critical driver of the immunological response during COVID-19 disease.^226^ In response to SARS-CoV-2 infection, human macrophages activate inflammasomes, release IL-1 and IL-18, and undergo pyroptosis, thereby contributing to the hyperinflammatory state of the lungs.^226^ Together, these results suggest that the inflammasomes oppose host infection by production of inflammatory cytokines and suicide by pyroptosis to prevent a productive viral cycle.^226^

Neutrophils are also recruited to the sites of viral infection.^227,228^ They are effector cells that produce reactive oxygen species that damage infected tissues.^229^ The damage caused by the neutrophils contributes to the virus clearance and the elimination of invasive pathogens.^230^ The virus infection and excessive oxidative stress can induce the release of damage-associated molecular pattern (DAMP), which can act as a proinflammatory stimulus.^230^ Neutrophilia in the lungs has been associated with enhanced tissue injury and pneumonia in COVID-19 patients, and the increase in the neutrophil/lymphocyte ratio (NLR) has been suggested as a risk factor for disease severity.^231^ Activation of neutrophils can also promote the formation of neutrophil extracellular traps (NETs).^213,230^ NET release was also induced by infective SARS-CoV-2 in neutrophil culture cells *in vitro* but not by an inactive virus, suggesting that the process of viral infection may be a trigger for NET induction.^232^ As part of NETs, neutrophils die, releasing their DNA and other bioactive molecules, which further reinforce the inflammatory response and enhance the prothrombotic disbalance.^233^ The formation of NETs can constrain the spread of a pathogen through circulation as well as lead to the release of antimicrobial compounds.^230,233^ Post-mortem histological data from patients with severe COVID-19 described increased numbers of degenerated neutrophils, indicating NET formation.^234^ Additionally, the procoagulation stimulus of the NET has been associated with the systemic manifestation of vascular disbalance, thrombi formation in the microvasculature of several organs, and ARDS.^233^

The respiratory system is the primary site of virus-induced immunopathology.^235^ In some cases the virus reaches the lower respiratory tract and infects the alveolar cell lining and pneumocytes I and II,^236^ leading to the secretion of immune mediators that activate the endothelial cells.^237^ Activation of the endothelium weakens epithelial barrier function, which increases the influx of fluid from circulation to the surrounding interstitial area, leading to edema.^237^ The fluid is accompanied by the recruitment of immune cells such as monocytes and neutrophils, which cross the endothelial barrier driven by the secretion of chemokines such as CCL2, CCL3, CCL7, and CXCL10.^211^ In the surrounding tissue, these cells enhance the damage associated with exaggerating inflammation and contribute further to thrombi formation.^215^ The hyperinflammatory response leads to disorder of the pneumocyte’s alveolar lining and ARDS, disrupting the gas exchange function.^238^

Once the virus reaches the bloodstream, it can spread and infect other cells.^235^ It has been shown that vascular manifestations are caused not by SARS-CoV-2 infection of blood vessel cells but rather by viral-inflammation-induced endothelial activation and barrier disruption.^239,240^ This has been associated with the formation of thrombi in the microvasculature and ischemia in the limbs and extremities of COVID-19 patients.^241^ The resulting intravascular coagulation has stimulated the use of anticoagulant therapy in these patients.^242^

Abnormal blood parameters, such as D-dimer and fibrinogen levels, are also reported in critical COVID-19 patients.^243^ These abnormalities could reflect the excessive inflammation caused by elevated levels of the proinflammatory cytokine IL-6.^178^ Other abnormal parameters found in patients with severe illness are elevated C-reactive protein (CRP) and lactate.^244−246^ Once the endothelium becomes infected, the virus induces an immune response, mainly mediated by neutrophils and lymphocytes, against the related endothelial tissues, triggering endotheliitis.^239^ However, other reports have shown the opposite results, suggesting that endothelial cells are not efficiently infected by SARS-CoV-2.^240^ A cytokine storm can also drive vessel leakage syndrome, which is characterized by uncontrolled efflux of fluid from circulation to surrounding tissues.^223,247^

SARS-CoV-2 can spread to other organs such as the heart, kidney, and liver.^178^ Cardiac injury is a common condition among hospitalized COVID-19 patients, and it is associated with a higher risk of mortality.^17,248^ A cumulative body of data has demonstrated that SARS-CoV-2 infection can cause both direct and indirect cardiovascular sequels, including arrhythmias, cardiogenic shock, acute coronary syndrome (ACS), myocardial injury, cardiomyopathy, acute cor pulmonale, and thrombotic complications.^249,250^ Similarly, cardiac injuries have also been associated with infection by other highly pathogenic CoVs, including SARS-CoV and MERS-CoV.^251^ Autopsy results have shown interstitial myocardium infiltration of mononuclear cells,^19^ and myocarditis has been reported in more than 20% of the patients hospitalized in the ICU.^252^ Despite not being a specific marker, the increased level of troponin can suggest myocardial damage, and abnormal high levels are observed in patients with COVID-19.^253^ Other heart abnormalities in COVID-19 patients have also been reported, including cell necrosis, dysfunctionality of myocytes, arrhythmia, and even cardiac arrest.^254^

Acute kidney injury is a complication that is frequently reported in critical-condition patients and is associated with mortality, resulting in proteinuria, hematuria, and leukocyturia.^255−257^ The formation of thrombi in the microvasculature of the kidney in association with NET formation has also been reported.^258^ Unlike the lungs, SARS-CoV-2 infection of the kidneys occurs later in the course of the disease when other more severe symptoms have already been exhibited. As a result of this process, kidney damage can be a consequence of a hyperinflammation response, cytokine storm, and hypoxia.^221,223,259^ Hyponatremia, hypochloremia, hypocalcemia, and acidosis are common electrolyte abnormalities associated with the high cell turnover seen in COVID-19 patients with acute kidney injury.^260,261^ Direct SARS-CoV-2 infection of kidney cells has been reported using *in vitro* and post-mortem studies and may also contribute to local inflammation and kidney damage.^262−264^

The gastrointestinal (GI) tract is also a system affected by coronaviruses in animals and has been associated with life-threatening infections.^265^ GI symptoms in COVID-19 are usually self-limited and include diarrhea, vomiting, nausea, abdominal pain, and discomfort.^266,267^ Similarly, other coronaviruses, such as SARS-CoV and MERS-CoV, have been associated with GI symptoms in some patients.^266^ Enterocytes can be productively infected by SARS-CoV-2,^268,269^ and virus particles have been observed in stool samples.^270^ This suggests that direct viral damage could be the cause of enteric manifestations. In some patients, the development of enteric symptoms can precede respiratory symptoms.^265,271^ Although fecal–oral transmission is a possible route for SARS-CoV-2 infection of the GI tract,^272^ the virus spreads from person to person mainly through direct contact or airborne transmission.^12,13^

Liver damage can also result from systemic hyperinflammatory responses such as cytokine storms, NET-mediated coagulation, and hypoxia,^178^ and altered liver function has been identified in more than 50% of hospitalized patients.^273^ Elevated levels of aminotransferases, such as alanine aminotransferase and aspartate aminotransferase, together with a slight increase in bilirubin levels have been associated with SARS-CoV-2-related liver injuries.^274^ Müller and colleagues demonstrated that SARS-CoV-2 can infect cells of the human exocrine and endocrine pancreas in both *in vivo* and *ex vivo* models.^275,276^ However, recent evidence suggests that despite the susceptibility of all pancreatic cell types to SARS-CoV-2, viral infection leads to only modest cellular alterations and inflammatory responses. Interestingly, infection by SARS-CoV-2 could lead to new-onset diabetes,^277^ and further studies to explore this possibility are certainly warranted.

Infection by SARS-CoV-2 has been associated with a range of neurological complications, and viral RNA and virus particles have been found in post-mortem analysis of brain tissue, suggesting that SARS-CoV-2 is neuroinvasive and neurovirulent.^278,279^ During SARS-CoV-2 infection, the most common neurological symptoms reported range widely in severity; these include mild symptoms, such as sensorial disturbance, headache, hyposmia, hypogeusia, confusion, and dizziness,^278,280^ and severe symptoms, such as consciousness disorders, seizures, and paralysis.^278,280−282^ Neurological disorders such as acute inflammatory demyelinating polyneuropathy (Guillain-Barré syndrome) have also been documented in some COVID-19 patients.^283,284^ The virus’s main route to the nervous system is through the bloodstream, although alternative pathways through the cribriform plate or ethmoid bone have been suggested.^285^ Direct viral damage associated with the hyperinflammatory response, hypoxia, and metabolic disorders are the mechanisms thought to be involved in neurological COVID-19.^285,286^ Additionally, other severe neurological complications documented in COVID-19 patients include hemorrhagic posterior reversible encephalopathy syndrome, meningoencephalitis, and acute necrotizing encephalopathy.^281,287−289^

The mechanisms underlying the taste and olfactory dysfunctions have been the focus of many scientific studies. It has been shown that SARS-CoV-2 can result in downregulation of olfactory receptors and their signaling pathways,^290^ although the virus does not seem to directly infect the sensory neurons of the olfactory epithelium in COVID-19 patients.^291^ SARS-CoV-2 can infect a myriad of cells in the oral cavity, including human type II taste cells,^292,293^ which may explain the taste dysfunction during and after acute COVID-19.

Despite ethical concerns of studies that challenge humans with SARS-CoV-2, scientists recently infected 36 healthy naïve volunteers (male and female) in the U.K. aged 18–29 years with a low dose of SARS-CoV-2 (10 TCID_50_ of a wild-type virus) intranasally under controlled conditions.^294^ That study provided detailed insights into SARS-CoV-2 infection. Symptoms started to develop very quickly, on average about 2 days after contact with the virus.^294^ Interestingly, the infection first appeared in the throat, and the infectious virus peaked at about 5 days during the clinical course of the infection, and at that stage it was significantly higher in the nose (peaking at ∼8.87 log_10_ copies/mL) than in the throat.^294^ In addition, the results demonstrated that mild to moderate symptoms were reported in 89% of the infected participants (*n* = 16), beginning 2–4 days after inoculation, whereas 11% of the participants (*n* = 2) remained asymptomatic.^294^ Together, these results provide relevant insights into viral kinetics throughout primary infection with SARS-CoV-2 and represent the first SARS-CoV-2 human challenge study in young adults.

## Diagnosis

**Figure 9:**
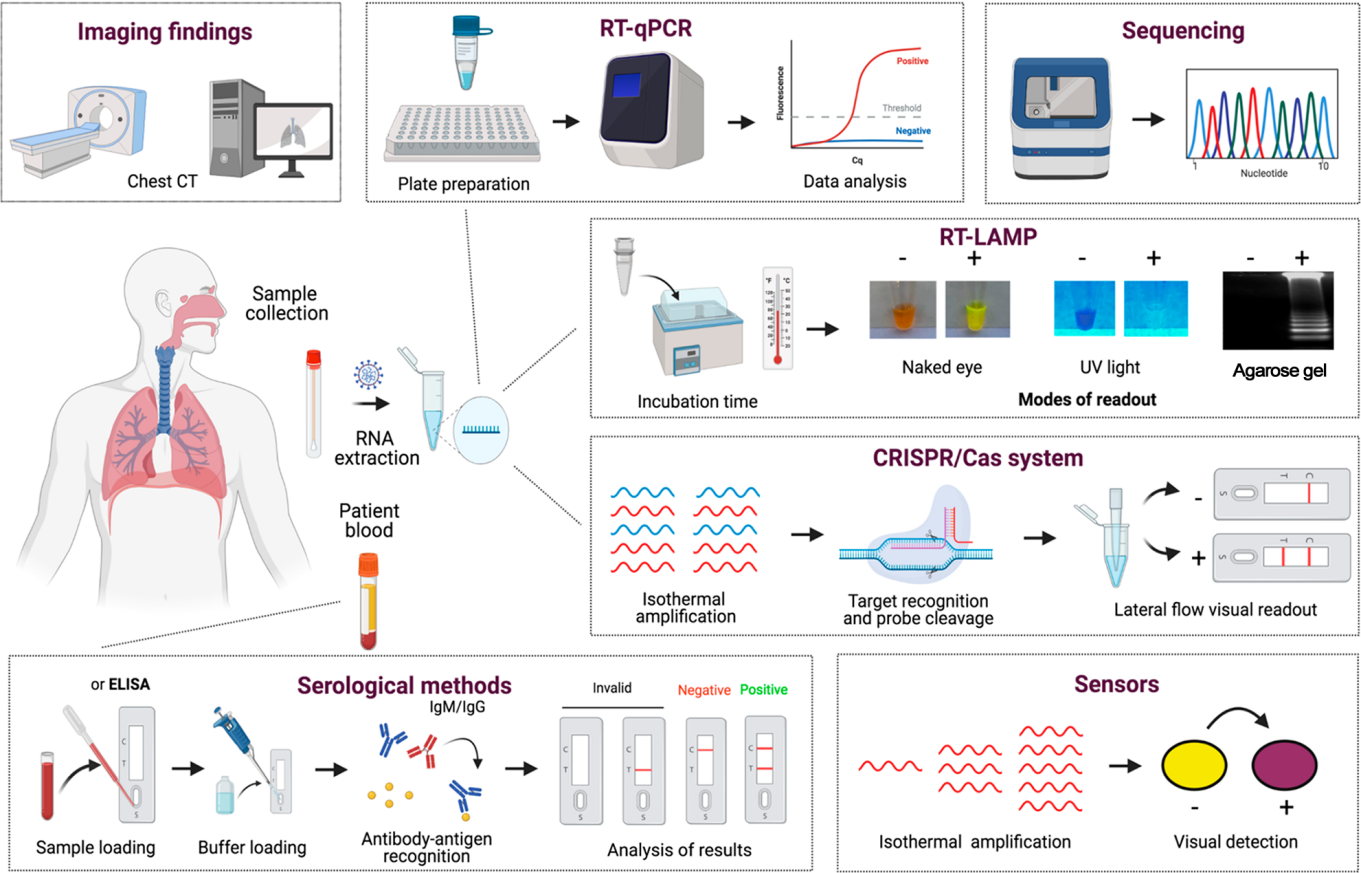
Overview of different methods for COVID-19 diagnosis. SARS-CoV-2 can be directly detected in humans using molecular approaches, such as RT-qPCR, DNA sequencing, RT-LAMP, CRISPR/Cas systems, and sensors. Imaging tests, including chest computed tomography (CT), have been widely used as a complementary approach to diagnose COVID-19 patients. Additionally, human antibodies produced against SARS-CoV-2 antigens can be detected in blood samples *via* serological methods, including enzyme-linked immunosorbent assay (ELISA), chemiluminescence immunoassay (CLIA), immunofluorescence assay (IFA), and lateral flow assay (LFA). This figure was created with Biorender.com.

Early diagnosis is essential for contact tracing, identification of hot-spot areas with active community transmission, and control of the spread of SARS-CoV-2.^150,295,296^ Current confirmation of COVID-19 disease can be achieved through clinical symptoms, imaging findings, biomarker evaluation, nucleic acid tests, and serological methods. Briefly, direct tests are used to detect the presence of viral particles, virus antigens, or viral RNA, while indirect tests are used to detect the immunological response against SARS-CoV-2 in infected patients, particularly to detect immunoglobulin M (IgM) and IgG antibodies.^150^ In this section, we provide an overview of the clinical course of COVID-19 reported in infected patients. Moreover, we explore the different detection methods being developed or used for SARS-CoV-2 diagnosis, discussing their advances, principles, advantages, and limitations (Figure 9).

### Clinical Course

Understanding the temporal dynamics of viral shedding and immune response in patients with COVID-19 is critical to correctly diagnose the SARS-CoV-2 infection. Since the beginning of the pandemic, viral shedding profiles of COVID-19 patients have been investigated.^116,297−299^ A recent meta-analysis study analyzed the viral load dynamics, duration of viral RNA shedding, and viable virus shedding of SARS-CoV-2 in several body fluids.^300^ Using 79 studies (5340 individuals), the report demonstrated that the mean durations of SARS-CoV-2 RNA shedding were 14.6 days (95% CI 9.3–20.0 days; seven studies, 260 individuals) in the lower respiratory tract, 17.0 days (95% CI 15.5–18.6 days; 43 studies, 3229 individuals) in the upper respiratory tract, 16.6 days (95% CI 3.6–29.7 days; two studies, 108 individuals) in serum samples, and 17.2 days (95% CI 14.4–20.1 days; 13 studies, 586 individuals) in stool.^300^ The longest durations of SARS-CoV-2 RNA shedding were 59 days in the lower respiratory tract, 83 days in the upper respiratory tract, 60 days in serum, and 126 days in stool.^300^ It was found that the peak occurs in the first week of the disease, while for SARS-CoV and MERS-CoV RNA the peaks occur in the ranges of 10–14 and 7–10 days, respectively.^300^ In patients with severe disease, the viral load appears to reach its highest level in the third and fourth weeks, while in patients with comorbidities, viral persistence is continuous.^301,302^ However, recent findings showed that infectious particles could not be detected beyond day 9 of disease.^300^ Therefore, SARS-CoV-2 isolation from respiratory samples should use specimens collected during the initial stages of COVID-19 that present a low cycle threshold (Ct < 24) on RT-qPCR.^303^ Typically, nucleic acid tests are commonly used to detect and amplify the SARS-CoV-2 genome from several types of specimens, including nasopharyngeal swabs, oropharyngeal swabs, or other upper respiratory tract samples.^304^ By the use of RT-qPCR, SARS-CoV-2 RNA is detected as early as day 1 of symptoms and peaks within the first week of symptoms onset.^304^ This positivity starts to decline by week 3, and subsequently SARS-CoV-2 RNA becomes undetectable.^304^ However, the viral load of severe COVID-19 cases was estimated to be 60 times higher than that of mild cases,^299^ and subsequently SARS-CoV-2-positive RT-qPCR may persist beyond 3 weeks after disease onset, while most mild cases will present a negative result.^304,305^

**Figure 10:**
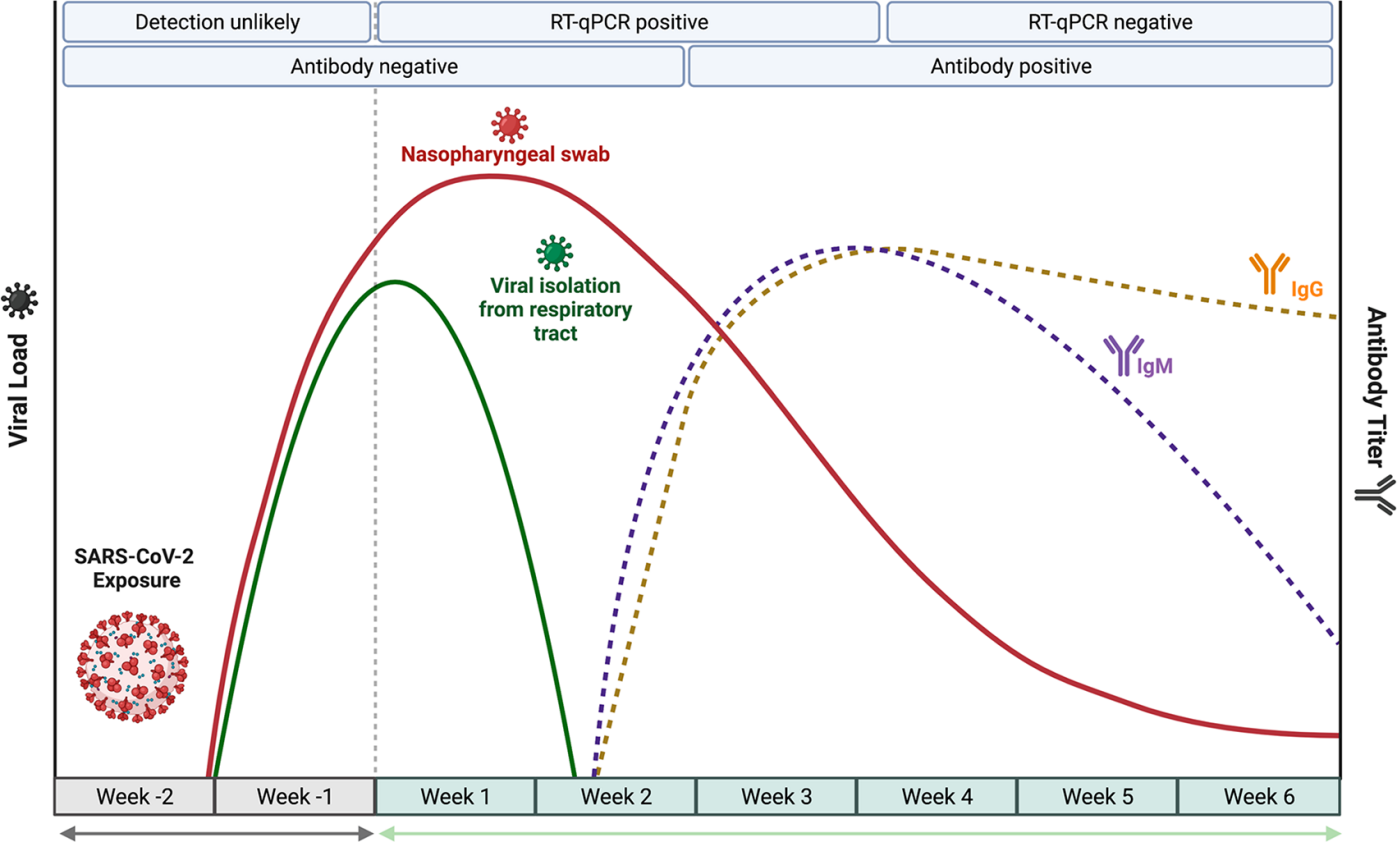
Kinetics of viral load and immune response during SARS-CoV-2 infection. During the first week after SARS-CoV-2 exposure, a period when patients are typically presymptomatic, the viral load increases and reaches its peak during the initial days after symptom onset. Seroconversion in infected patients begins in the second week after symptom onset. Three to four weeks after symptom onset, the IgM and IgG levels both reach their peaks and then begin to drop—more rapidly for IgM than for IgG. To avoid false-negative results when COVID-19 diagnostic tests are performed, the kinetics of viral load and immune response should be taken into consideration. The figure was adapted from the template in Biorender.com.

The host immune response to SARS-CoV-2 infection has also been investigated.^306−310^ In most COVID-19 patients, IgM levels increase during the first week after SARS-CoV-2 infection, reach their peak after 2 weeks, and subsequently fall back to near-background levels.^311^ Similarly, IgG is detectable 1 week after disease onset and is maintained at a high level for a long period, even more than 48 days.^312^ Recent studies found that seroconversion for IgG and IgM occurred simultaneously or sequentially, and both IgG and IgM titers plateaued within 6 days after seroconversion.^307^ In a large study with 285 patients with COVID-19, 100% of patients tested positive for IgG within 19 days after symptom onset.^307^ During the host immune response against SARS-CoV-2 infection, COVID-19 can be detected indirectly using serological methods, particularly detection of IgM and IgG. Briefly, Figure 10 describes how to interpret two types of diagnostic approaches commonly used for SARS-CoV-2 diagnosis (RT-qPCR and serological methods) and how the results may vary over time during COVID-19 clinical progression.

### Imaging Findings in COVID-19 Patients

In the early stage of SARS-CoV-2 infection, symptoms are usually nonspecific. This makes clinical diagnosis difficult, especially in areas with the circulation of other respiratory viruses, such as influenza virus and human rhinovirus (HRV), and even nonrespiratory pathogens such as dengue virus (DENV).^148,295,313^ Because of the nonspecific clinical manifestation of the disease, chest computed tomography (CT) has been widely used as a complementary tool in the investigation of COVID-19 patients. It has been used to evaluate the disease progression and assess the impairment of the lower respiratory tract and other anatomical areas by the disease.^314,315^ Chest CT scan images have been used to check for possible abnormalities suggestive of lower respiratory tract disease, such as viral pneumonia, eventually caused by SARS-CoV-2.^316^ Typically, the most important imaging changes observed in COVID-19 patients are multilobe lesions in both lungs and bilateral and peripheral ground-glass opacity (GGO) with or without consolidated changes.^314^ Other findings include rounded opacities, a crazy-paving pattern, an air bronchogram, and septal thickening mainly distributed in peripheral and posterior areas.^317,318^ It has been suggested that during patient clinical management, CT scanning combined with RT-qPCR should be used in the routine for the diagnosis of patients with a high clinical suspicion of COVID-19 who had a negative result on the RT-qPCR assay.^319^

### Clinical Biomarkers in COVID-19 Patients

Besides the laboratory methods for detecting SARS-CoV-2 discussed throughout this review, many studies have demonstrated that hematological, biochemical, and blood chemical alterations in COVID-19 patients are possible markers of disease progression and patient health.^244,320−323^ The levels of these markers fluctuate depending on the clinical course of the disease, and since they can be assessed by routine blood tests, additional testing can be ordered by physicians on the basis of patients’ clinical evolution. Patients with increasing SARS-CoV-2 severity often have leukocytosis, leukopenia, decreased albumin levels, increased levels of lactate dehydrogenase (LDH), CRP, bilirubin, and creatinine kinase, and a high erythrocyte sedimentation rate (ESR).^324^ In general, no individual biomarker can be used to confirm or discard COVID-19 diagnosis, and diagnostic testing should be conducted for all suspected cases.

It should be noted that some clinical biomarkers have an important value for patient management since they can be used to assess the progression of the disease and its severity and even act as risk factors for death. Compared with healthy individuals, clinical biomarkers associated with increased disease severity in patients with COVID-19 include lymphopenia, thrombocytopenia, and high levels of liver alanine aminotransferase (ALT), aspartate aminotransferase (AST), LDH, CRP, and ferritin.^321,325^ A meta-analysis study demonstrated that patients with fatal COVID-19 disease progression had significantly increased white blood cell (WBC) count and decreased lymphocyte and platelet counts compared with nonsevere illnesses and survivors.^326^ Additionally, it was found that biomarkers of inflammation, cardiac and muscle injury, liver and kidney dysfunction, and coagulation measures were also significantly elevated in patients with both severe and fatal COVID-19.^326^ Elevated levels of serum biomarkers IL-6, IL-10, ferritin, CRP, and cardiac troponin acted as strong discriminators for disease severity and were associated with an increased risk of death.^246,326,327^

### RT-qPCR

RT-qPCR is currently considered the gold-standard lab method for the diagnosis of SARS-CoV-2.^150^ Because of its high sensitivity and specificity, this technique allows detection of viral RNA in the first days after symptom onset during the initial stages of the disease or even during presymptomatic or postsymptomatic phases.^311,328^ Choosing the correct specimen for testing is a critical step to produce a reliable diagnosis.^150^ SARS-CoV-2 RT-qPCR is most often performed on upper respiratory specimens, which include nasopharyngeal or oropharyngeal swabs, aspirates or washes, sputum, and bronchoalveolar fluids.^138,328−330^ In addition to respiratory tract samples, SARS-CoV-2 detection by RT-qPCR has been documented in other specimens such as blood, urine, anal swabs, ocular secretions, breast milk, semen, and feces.^138,331−335^ Because of the discomfort associated with respiratory tract sampling, the need for trained healthcare personnel, and the risk of aerosol or droplet production, there is a great interest in alternative methods to collect samples from COVID-19 patients. Less invasive samples such as saliva and gargle lavages (“mouthwashes”) are promising alternatives for use in the routine, especially in patients with a high viral load.^336−339^

Since the emergence of SARS-CoV-2, many molecular assays based on RT-qPCR have been developed or are being used by clinical, research, and public health laboratories for the diagnosis of COVID-19.^340−343^ In addition to assays recommended by the WHO, many molecular RT-qPCR kits have been approved by the U.S. Food and Drug Administration (FDA) and are widely available for the detection and amplification of SARS-CoV-2 RNA (https://www.fda.gov/medical-devices/emergency-situations-medical-devices/emergency-use-authorizations). A variety of molecular targets within the SARS-CoV-2 genome have been used, with most assays targeting one or more genes, such as the spike (S), envelope (Env), nucleocapsid (N), RNA-dependent RNA polymerase (RdRp), and open reading frame (ORF) genes.^304^ In the initial stages of the COVID-19 pandemic, dual- or multigene detection strategies were adopted for RT-qPCR assays to ensure assay specificity.^344^ As the pandemic evolved and the disease prevalence increased, many laboratories around the world implemented a workflow using single-target detection of SARS-COV-2.^315^ To minimize false negatives associated with technical errors, internal control (IC) targeting human “housekeeping” transcripts like RNase P mRNA should be included during the testing of patient’s samples by RT-qPCR.^345^

Four of the most commonly used SARS-CoV-2 RT-qPCR assays have been developed by the China National Institute for Viral Disease Control and Prevention,^342^ the U.S. Centers for Disease Control and Prevention (CDC),^341^ Charité Institute of Virology, Universitätsmedizin Berlin (Charité),^340^ and Hong Kong University (HKU).^343^ In this context, Vogels and colleagues evaluated the analytical efficiencies and sensitivities of these four primer–probe sets to detect SARS-CoV-2.^346^ The results demonstrated that all of the primer–probe sets can be used to detect SARS-CoV-2 at 500 viral RNA copies per reaction, except for the RdRp-SARSr (Charité), which presented low sensitivity.^346^ In another related study, Nalla and co-workers evaluated the performance of seven RT-qPCR protocols recommended by the WHO for detecting SARS-CoV-2 RNA from patient samples.^347^ It was found that the most sensitive assays were those that used the E-gene primer–probe set described by Corman *et al.*(340) and the N2 set developed by the CDC.^341^ In addition, the results demonstrated that all of the RT-qPCR assays evaluated were highly specific for SARS-CoV-2 detection, and no cross-reactivity against other respiratory viruses was reported.^347^

Throughout the COVID-19 pandemic, reference laboratories have faced global shortages of diagnostic supplies, especially for the RNA extraction step.^348−351^ To meet this need, simplification of nucleic acid tests by eliminating the RNA extraction step is being explored.^150,349^ Studies showed that skipping RNA extraction by simple direct heating of samples for 5 min at 95–98 °C resulted in sensitivity and specificity comparable to the standard RT-qPCR method,^349,351^ suggesting that direct RT-qPCR without an RNA extraction step is a viable option to perform the diagnosis of COVID-19 patients. Although this strategy is promising and has great potential for the application of diagnostic workflows in low-resource settings, particular attention should be given to the increase of false-negative results.^352^

More recently, with the emergence of SARS-CoV-2 variants that may increase transmissibility and/or cause escape from immune responses, there is an urgent need for the targeted surveillance of these circulating variants in laboratories around the world.^353^ Vogels and colleagues designed and validated a multiplex RT-qPCR assay to detect SARS-CoV-2 variants of concern (VOCs).^353^ Using detection of the deletion Δ3675–3677 in the ORF1a gene, they were able to indicate the presence of the emerging variants B.1.1.7 [alpha], B.1.351 [beta], and P.1 [gamma] since this mutation had not already been detected in other SARS-CoV-2 variants. Detection of the deletion Δ69–70 in the S gene was applied to differentiate these three lineages.^353^ It will be crucial that optimization and validation of diagnostic tests continue as the COVID-19 pandemic evolves, since new variants of SARS-CoV-2 may emerge and existing assays must be constantly evaluated to ensure that the diagnosis is performed with high efficiency and accuracy.

### Genome Sequencing

Although genome sequencing does not play a critical role in routine laboratory diagnostics for SARS-CoV-2, it has gained relevance with the emergence of new viral variants because it is essential for tracking changes in the viral genome over time and tracing transmission patterns.^150,354,355^ To date, only a limited number of reports have explored the use of NGS for SARS-CoV-2 detection with diagnostic purposes.^356−358^ For instance, Bloom and colleagues developed a protocol using NGS, called SwabSeq, to detect SARS-CoV-2 RNA in patient samples, including nasal or saliva samples, in a single run without the need for RNA extraction.^357^ The authors incorporated a synthetic RNA standard that facilitates end-point quantification and the calling of true negatives and reduces the requirement for automation, purification, and sample normalization. After 80 000 tests performed within 2 months, the results revealed an analytical sensitivity and specificity comparable to or better than the RT-qPCR reference method. Recent findings support the potential of NGS as a diagnostic tool for SARS-CoV-2 detection, although practical difficulties like high cost, shortage of globally available supplies, the need for a specialized laboratory infrastructure, sophisticated instrumentation, bioinformatics expertise, and well-trained staff may pose significant bottlenecks to its use for confirming COVID-19 infection, especially in low-resource countries.^359^ Another limitation of NGS technologies is their low analytical sensitivity, which may negatively affect the analysis of results.^315^ For NGS sequencing, the use of specimens with high viral loads (low Ct values) is recommended to generate high-quality results. Samples with low viral loads (high Ct values) can generate insufficient data or poor-quality results for subsequent analyses.^356,360^

Notably, sequencing protocols based on NGS (*e.g.*, Illumina, BGI MGISEQ2000, and Nanopore [MinION]) and Sanger methods are being used to rapidly generate SARS-CoV-2 genome sequences from patient samples.^7−9^ As of June 15, 2022, 11 416 875 genome sequences have been deposited on the Global Initiative on Sharing All Influenza Data (GISAID) (https://www.gisaid.org/), including whole-genome sequences from COVID-19 patients from different countries around the world. To shed light on the ongoing evolution of the SARS-CoV-2 genome during the pandemic, sequencing is essential in order to identify mutations that may be associated with escape from vaccine-induced immunity or natural-induced immunities, diminishment of the performance of current diagnostics, and increased transmissibility and/or lethality.^361,362,353,363−365^ Thus, the development of rapid and low-cost sequencing protocols is crucial for discriminating all emerging SARS-CoV-2 variants as the pandemic evolves. To address this question, Bezerra and co-workers proposed a rapid and accessible protocol based on Sanger sequencing of a single PCR fragment that can identify and discriminate SARS-CoV-2 VOCs.^359^ They evaluated 12 samples from Brazilian patients using both NGS and Sanger sequencing approaches. Taken together, the findings from the Sanger sequencing matched the NGS results 100%.^359^ Moreover, this protocol allows a much broader network of laboratories to perform molecular surveillance of SARS-CoV-2 VOCs and report results within a shorter timeline, especially to increase sequencing capacity in low- and middle-income countries. In view of the evolving nature of the SARS-CoV-2 genome, genomic surveillance should be conducted and implemented on a large scale to allow early identification of new variants and to help establish policies for controlling the viral spread.

Even though it is not routinely used for SARS-CoV-2 detection in reference laboratories, genome sequencing of SARS-CoV-2-positive samples combined with computational tools has paved the way for many applications, including investigations of disease pathogenesis, diagnostics, vaccines, antiviral drugs, molecular epidemiology, viral evolution, cell receptor binding, possible viral hosts, and host antiviral immune response.^8,60,366−372^

### RT-LAMP

Although RT-qPCR is currently the reference method for the diagnosis of SARS-CoV-2, it has several limitations, including long processing time, the requirement of highly specialized manpower, and the involvement of costly and specialized equipment for amplification and detection of the viral genome.^150,373^ These barriers make the technique unsuitable for large-scale applications and negatively impact the establishment of effective disease control programs in low- and middle-income countries. A recent modeling study concluded that effective screening is more impacted by the frequency of testing and sample-to-answer time than the analytical sensitivity of the test, highlighting the role of rapid tests for COVID-19 control.^374^ With this in mind, a variety of techniques have been developed to detect SARS-CoV-2 at the point of need.^315^ Isothermal techniques like reverse transcription loop-mediated isothermal amplification (RT-LAMP) are perhaps among the most promising methods for rapid detection of SARS-CoV-2. RT-LAMP has several advantages compared with RT-qPCR, since reactions are conducted at a constant temperature, eliminating the need for expensive equipment to perform the assay and allowing the test to be carried out in the field.^373,375−377^ Following the isothermal incubation in as little as 20 to 60 min, usually the results can be easily interpreted by naked-eye analysis through a color change of the reaction tube,^373,377^ although other different approaches can be used to visualize the results of the RT-LAMP reaction.^376^

Considering its advantages of high specificity and sensitivity, rapid amplification, simple operation, and low cost, RT-LAMP has potential applications for the diagnosis of many infectious diseases.^376,378^ Since the emergence of SARS-CoV-2, many RT-LAMP assays have been developed for the diagnosis of this virus in several types of clinical samples, including saliva, serum, nasopharyngeal swabs, oropharyngeal swabs, and urine.^370,379−386^ In general, RT-LAMP assays have been designed for different targets in the SARS-COV-2 genome (ORF1ab, N protein, E protein, RdRP, and M protein), and the clinical performances of many RT-LAMP assays have been compared with that of RT-qPCR.^315,385,387^ Efforts to decrease the cost and simplify the RT-LAMP workflow for testing patient samples are in progress using protocols without RNA extraction from patient specimens and in-house-produced enzymes,^388−390^ with the possibility to scale up COVID-19 diagnostics. With regard to the limit of detection (LoD), the majority of RT-LAMP assays should have LoDs ranging from 200 to 100 copies per reaction,^382,383^ while a few have demonstrated LoDs as low as 10 copies per reaction or even 1 copy per reaction.^391−393^ Currently, there are 14 diagnostic devices based on RT-LAMP assays that have been granted Emergency Use Authorization (EUA) by the FDA (https://www.fda.gov/medical-devices/coronavirus-disease-2019-covid-19-emergency-use-authorizations-medical-devices/in-vitro-diagnostics-euas-molecular-diagnostic-tests-sars-cov-2). Taken together, these developments highlight the potential of RT-LAMP-based methods to improve SARS-CoV-2 diagnosis on site in nearly real time, mainly for hot spots (*e.g.*, care homes, small cities) and remote areas (*e.g.*, rural communities) with limited laboratory infrastructure.

### CRISPR/Cas-Based Systems

Another category of nucleic acid tests that could be used to detect SARS-CoV-2 RNA is the clustered regularly interspaced short palindromic repeats (CRISPR)/Cas machinery.^394^ CRISPR systems are a fundamental part of a microbial adaptive immune system against foreign nucleic acids, and when activated, the machinery guides Cas proteins to recognize and cleave specific nucleic acid sequences.^395−398^ Thus, understanding of the mechanism and features of the CRISPR/Cas system over the past few years has led to many technological advances in genome editing and highlighted the use of the CRISPR/Cas system for diagnostic applications such as the detection of RNA viruses.^394,399−404^ Briefly, the CRISPR/Cas system is programmed to cleave specific nucleic acid sequences in the RNA/DNA target, and their cleavage can be detected by fluorescence or lateral-flow readouts.^405^ One of the first CRISPR/Cas-based detection methods, specific high-sensitivity enzymatic reporter unlocking (SHERLOCK), was described in 2017. In combination with isothermal preamplification, SHERLOCK was applied to the detection of specific strains of Zika and dengue viruses, distinguishing pathogenic bacteria, genotyping human DNA, and identifying mutations in cell-free tumor DNA from patients’ liquid biopsy samples.^402,403^ The same system was rapidly adapted to detect SARS-CoV-2 RNA.^405−407^ The suitability of SHERLOCK technology for the detection of SARS-CoV-2 was evaluated using 154 nasopharyngeal and throat swab samples. With a LoD of 42 RNA copies per reaction, the SHERLOCK platform was 100% specific and 96% sensitive in agreement with RT-qPCR.^405^ More recently, De Puig and colleagues developed a low-cost test based on the SHERLOCK system to perform diagnosis of SARS-CoV-2 and emerging variants.^401^ The authors achieved high sensitivity within 1 h and demonstrated multiplex detection of SARS-CoV-2 and mutations associated with emerging variants, including B.1.1.7 (alpha), B.1.351 (beta), and P.1 (gamma).^401^

Another technology based on the CRISPR/Cas system called DETECTR (for endonuclease-targeted CRISPR *trans* reporter) was developed by Mammoth Biosciences Company to detect any DNA or RNA target. More recently, this technology was combined with an RT-LAMP assay for the detection of SARS-CoV-2 from patient samples including nasopharyngeal or oropharyngeal swabs within 40 min.^408^ A clinical performance study using 78 samples from COVID-19 patients demonstrated that the DETECTR technology had 95% positive predictive agreement and 100% negative predictive agreement with RT-qPCR.^408^ In a larger patient cohort, the authors compared DETECTR with RT-qPCR to diagnose SARS-CoV-2 using samples from 378 patients.^409^ The results demonstrated a value of 95% reproducibility among methods and showed that when combined with RT-LAMP to detect SARS-CoV-2 RNA, DETECTR reached equal sensitivity in comparison to RT-qPCR.^409^ These findings highlight the promising potential of CRISPR/Cas-based diagnostic systems for the diagnosis of COVID-19 patients. However, most CRISPR-based workflows still require multiple liquid-handling steps including RNA extraction, reliable access to electricity, technical skills, and laboratory equipment like centrifuges, pipettes, and heating blocks,^401^ limiting its applicability in remote areas.

### Sensors

Sensors are devices that detect chemical or biological components by generating signals,^410^ and they represent cost-effective alternatives for use in clinical practice. The use of sensors eliminates the limitations faced by RT-qPCR and provides a decentralized, high-capacity, and low-cost diagnostic tool for use in low-resource settings.^411,412^ Given their advantages, many studies have focused on alternative sensor-based modalities for the diagnosis of COVID-19 patients. In summary, most sensors developed for SARS-CoV-2 are based on platforms previously used for the diagnosis of other viral pathogens and are currently being adapted for SARS-CoV-2 diagnostics.^150^ Detection of SARS-CoV-2 has been made with various types of sensors, including genosensors, immunosensors, electrochemical sensors, and electrical immunosensors.^411^

Paper-based sensors offer another promising alternative for COVID-19 diagnosis since they provide high sensitivity and specificity, simple operation, easy adaptability, low cost, and absence of cold-chain distribution requirements.^400,413^ Our previous studies on synthetic biology-based diagnostics have paved the road and opened doors for the use of cell-free (CF) reactions for the detection of emerging and re-emerging pathogens such as Zika virus, chikungunya virus, norovirus, and Ebola virus (EV).^400,414−417^ Importantly, our toehold switch sensors are programmable synthetic riboregulators that control the translation of a gene *via* the binding of a trigger RNA. Briefly, the switch sensors contain a hairpin structure that blocks gene translation by sequestration of the ribosome binding site (RBS) and start codon. If the target sequence is present, it activates the translation of a reporter (*e.g.*, LacZ) to create an optical signal through an enzymatic reaction that mediates a color change by converting a yellow substrate (chlorophenol red-*b*-d-galactopyranoside) to a purple product (chlorophenol red).^400^ In a effort to provide accessible diagnostics for the COVID-19 pandemic, we adapted this system for SARS-CoV-2 diagnostics and achieved high sensitivity (as few as 100 RNA copies) and high specificity against other respiratory pathogens, including H1N1, H7N9, and MERS-CoV.^418^ These results illustrate the potential of sensor-based tools to respond to global crises.

### Serological Methods

Since the emergence of SARS-CoV-2, a variety of serological methods have been developed and commercialized, and a list of authorized COVID-19 serological assays in the U.S. is updated daily.^419^ They play an adjunct role to molecular assays to allow the identification of suspected patients that have tested negative by nucleic acid tests.^315^ Moreover, serological measurements could also be used in seroprevalence studies to determine past exposures to SARS-CoV-2 in the human population, assess attack rates in defined populations or geographical areas, and evaluate vaccine efficacy.^150,315^

Different serology-based assay platforms have been developed to date, including lateral flow assay (LFA), enzyme-linked immunosorbent assay (ELISA), chemiluminescence enzyme assay (CLIA), and immunofluorescence assay (IFA).^420^ Of these assays, LFA, ELISA, and CLIA are used as first-line methods in the routine to confirm COVID-19 infection.^150^ Serological methods often use recombinant antigens to detect one or more immunoglobulin isotypes (*i.e.*, IgA, IgM, or IgG), with the S protein, S RBD, and N protein being the most commonly used antigens because of their high antigenicity.^80,421,422^ IgM and IgG are widely used in serological methods for SARS-CoV-2, while IgA detection is less common.^423^ Recent reports have shown that serological methods using the S antigen are more sensitive than the N-antigen-based methods.^420^ Moreover, it was suggested that an N-based assay is more cross-reactive with other anti-human coronavirus antibodies than an S-based assay.^307,424^ Within the S protein, the S1 subunit is a major immunodominant epitope produced in the response against SARS-CoV-2 infection, suggesting that this subunit is a promising candidate for use during the development of serological assays.^425,426^ The typical time required to detect immune responses to SARS-CoV-2 infection is around 1 to 2 weeks. Therefore, serological methods have limited utility for SARS-CoV-2 diagnostics in the acute stages of illness but have the best performance when used during the late phase of SARS-CoV-2 infection.^315^ High sensitivity, specificity, and overall accuracy are desired for serological assays, but concerns about the presence of high rates of false-positive and false-negative results have been raised, and the overall accuracy of many tests has not been well-defined.^427,428^ As a result, the understanding of the key parameters in terms of development and validation of diagnostic assays is critical for the correct interpretation of results.^352,427^ To overcome these drawbacks, serological methods must be developed following a standard guide as a reference, and after its development, the assay should be validated with adequate patient specimens representative of a real-world scenario.^427,428^ Assays that measure neutralizing antibody titers such as microneutralization, plaque reduction neutralization test (PRNT), and their variations are largely used to assess immunity against SARS-CoV-2 since neutralizing antibodies induced by natural infection or vaccination are highly preditive of protection.^429,430^

### Antigen-Based Tests

Antigen detection tests are based on the identification of SARS-CoV-2 proteins by immunological reactions. Despite their lower sensitivity compared with molecular methods, they provide short turnaround times and are very useful for virus detection in samples with moderate to high viral loads.^431,432^ Since the emergence of SARS-CoV-2, extraordinary efforts by research groups and companies around the world have resulted in the development of many antigen tests to detect SARS-CoV-2.^433−437^ The suitability of antigen-based rapid tests (Ag-RDTs) for SARS-CoV-2 detection has been evaluated in a recent meta-analysis study using 214 clinical datasets including 112 323 patient specimens.^432^ For comparison, patient samples were also tested using RT-qPCR as a standard method. The results demonstrated a clinical sensitivity of 71.2% (95% CI 68.2% to 74.0%) and clinical specificity of 98.9% (95% CI 98.6% to 99.1%).^432^ The clinical sensitivity was considerably better in samples with lower RT-qPCR Ct values, *i.e.*, <20 (96.5%, 95% CI 92.6% to 98.4%) and <25 (95.8%, 95% CI 92.3% to 97.8%) in relative to those with Ct ≥ 25 (50.7%, 95% CI 35.6% to 65.8%) and ≥30 (20.9%, 95% CI 12.5% to 32.8%).^432^ In addition, it was found that when the test was performed in the first week of symptoms onset, the sensitivity was substantially higher (83.8%, 95% CI 76.3% to 89.2%) compared with testing after 1 week (61.5%, 95% CI 52.2% to 70.0%).^432^ These findings suggest that Ag-RDTs can detect SARS-CoV-2-infected persons within the first week of symptoms onset but have low utility for diagnostic purposes in the late phase of COVID-19 and are therefore of limited value.

## Treatment

Despite active research, few antivirals have shown potential benefit for COVID-19 patients. As of July 14, 2022, there were more than 700 drugs under development for COVID-19, and approximately 460 were in human clinical trials being reviewed by the FDA.^438^ Repurposed antiviral agents originally developed against influenza virus, human immunodeficiency virus (HIV), EV, and SARS-CoV/MERS-CoV viruses as well as antibiotics, antiprotozoals, and anthelmintic drugs are being investigated as potential therapeutic options for treating SARS-CoV-2 infection. These antivirals are proposed to inhibit the SARS-CoV-2 infectious cycle by targeting human cell receptors or viral proteins. The leading viral proteins targeted by anti-SARS-CoV-2 drugs include the RdRp, the helicase complex, and the 3-chymotrypsin-like (3CL^pro^) and papain-like (PL^pro^) viral proteases. Inhibitors for the human cellular receptor ACE2 and human proteases such as TMPRSS4, TMPRSS2, furin, and CatL have also been developed to block SARS-CoV-2 infection. Drugs and monoclonal/polyclonal antibodies designed for modulating the host response to the infection are an important additional part of the therapeutic arsenal that has been tested for treating COVID-19 patients. Despite the multitude of treatments that have been investigated throughout the past 2 years of the SARS-CoV-2 pandemic, which have been reviewed in detail elsewhere,^439^ a limited number of drugs have been investigated clinically, and an even smaller number have been approved for treating COVID-19.^438^

The U.S. National Institutes of Health (NIH) gathered a panel of experts to provide clinicians with evidence-based recommendations on the management of COVID-19. Immunomodulatory drugs have been tested for the treatment of COVID-19 with the goal of targeting the effects associated with the cytokine storm syndrome. According to the NIH COVID-19 treatment guidelines panel, the following immunomodulators are recommended for hospitalized patients according to their disease severity: corticosteroids (dexamethasone), IL-6 inhibitors (tocilizumab or sarilumab), and JAK inhibitors (baricitinib or tofacitinib). The panel does not recommend the use of colchicine for hospitalized patients and states that there is insufficient evidence for it to recommend either for or against the use of the following immunomodulatory drugs, except if done in a clinical trial: baricitinib plus tocilizumab, canakinumab, colchicine, intravenous immunoglobulin (except for MIS-C or when it is otherwise indicated), Bruton’s tyrosine kinase inhibitors (acalabrutinib, ibrutinib, zanubrutinib), JAK inhibitors other than baricitinib and tofacitinib (*e.g.*, ruxolitinib), and siltuximab (https://www.covid19treatmentguidelines.nih.gov/therapies/immunomodulators/summary-recommendations/). The early use of IFN has been shown to have a highly protective effect, but its use during the inflammatory phase or in the severe stages of the disease results in immunopathology and long-lasting harm for patients. Thus, the NIH panel recommends against the clinical use of systemic IFN-α, -β, or -λ for the treatment of COVID-19 (https://www.covid19treatmentguidelines.nih.gov/therapies/antiviral-therapy/interferons/).

On the basis of the most updated scientific evidence, the panel recommends five available treatment options as preferred or alternative therapies (listed in order of preference): nirmatrelvir combined with ritonavir (Paxlovid); sotrovimab; remdesivir; bebtelovimab; or molnupiravir (https://www.covid19treatmentguidelines.nih.gov/about-the-guidelines/whats-new/). In the section below, we describe the major approved and promising drugs for COVID-19 treatment. Importantly, as the pandemic evolves, we can still anticipate the discovery of new therapeutic options for use in clinical practice.

### Remdesivir

Remdesivir (GS-5734, Veklury) (Gilead Sciences) was the first drug to be approved by the main regulatory agencies around the world to treat COVID-19 patients (Table 2). Remdesivir is a bis(*S*-acyl-2-thioethyl) monophosphate prodrug that after chemical modification showed antiviral activity against hepatitis C virus (HCV), yellow fever virus (YFV), dengue virus 2 (DENV-2), influenza A virus (IAV), parainfluenza 3 virus (HPIV-3), and SARS-CoV *in vitro*, probably due to inhibition of viral RdRp by its triphosphate derivative.^440^ Remdesivir is a broad spectrum *in vitro* inhibitor for viruses from different families: EV and Marburg virus (MARV) (*Filoviridae* family); Nipah virus (NV), Hendra virus (HeV), measles virus (MV), and mumps virus (MuV) (*Paramyxoviridae* family); respiratory syncytial virus (RSV) and human metapneumovirus (hMPV) (*Pneumoviridae* family); Junín virus (JUNV) and Lassa virus (LASV) (*Arenaviridae* family); and SARS-CoV, MERS-CoV, and other human and bat CoVs (*Coronaviridae* family).^441−446^ Its triphosphate derivative inhibited RSV and HCV RdRp without inhibiting human RNA and DNA polymerases, and this activity is predicted to occur for RdRp from other viruses that present sequence similarity in motifs A and B of the nucleotide-binding regions of this enzyme.^441−443^ RdRp inhibition by a remdesivir triphosphate derivative is achieved by its incorporation into the nascent viral RNA, leading to delayed chain termination.^442,447^ For CoVs, the presence of exoribonuclease (ExoN)-mediated proofreading could diminish the sensitivity of the virus to remdesivir triphosphate derivative *in vitro*.^445^*In vivo* infection models showed that remdesivir is effective for treating EV infection in non-human primates,^441,442,448^ NV in non-human primates,^449^ and SARS-CoV and MERS-CoV infections in mice and non-human primates.^444,450,451^ In February 2020, Wang and colleagues published the first *in vitro* evidence of the efficacy of remdesivir in reducing SARS-CoV-2 infection in Vero E6 cells.^452^ Later, this activity was confirmed by additional studies using the same cell line^453,454^ and in human lung cells.^455^ Preclinical tests confirmed the anti-SARS-CoV-2 activity of remdesivir in mice and non-human primate models.^455,456^ Since the first *in vitro*/*in vivo* evidence, remdesivir has been clinically tested in patients with COVID-19. Four major randomized controlled clinical trials have investigated the benefits of remdesivir *versus* placebo/standard care for treating hospitalized COVID-19 patients.^457−460^ These studies showed that treatment of hospitalized COVID-19 patients with an intravenous infusion of 200 mg of remdesivir on the first day followed by 100 mg once daily for 5–10 days diminished the time to clinical improvement, enhanced the rates of recovered and discharged patients, and reduced the development of serious adverse events with no impact on mortality.^461^ These data supported the conditional or definitive approval of remdesivir as a therapeutic option for hospitalized COVID-19 patients in numerous countries (Table 2). Recently, efforts have been made to develop remdesivir derivatives for oral administration and with improved efficacy, but current data are preliminary.^462−464^

**Table 2:** Therapies Approved by the Main Regulatory Agencies Worldwide for Treatment of COVID-19 Patients^a^

		approval by regulatory agencies						
drug	category	FDA	EMA	PMDA	ANVISA	indication	route of administration/therapeutic scheme	benefits
Antiviral Therapies								
remdesivir (Veklury)	viral RdRp inhibitor	approved (2020/10/22)	conditional marketing authorization (2020/07/03)	special approval for emergency (2020/05/07)	approved (2021/03/12)	12-year-old or older COVID-19 patients that are hospitalized	intravenous infusion/attack dose of 200 mg on day 1 followed by 100 mg once daily	improves rates of recovery and discharge and reduces the development of serious adverse events^461^
casirivimab and imdevimab (REGEN-COV or Ronapreve)	recombinant human IgG1 monoclonal antibodies against SARS-CoV-2 spike protein	EUA (2020/11/21)	marketing authorization (2021/11/12)	special approval for emergency (2021/07/19)	approved for emergency use (2021/04/20)	12-year-old or older COVID-19 patients with mild to moderate disease who are at high risk for progression to severe COVID-19 or as postexposure prophylaxis	intravenous infusion or subcutaneous injection/single dose of 600 mg of casirivimab and 600 mg of imdevimab	reduces disease-related hospitalizations or deaths when administrated postinfection^474^
								reduces the risk of developing symptomatic or asymptomatic COVID-19 when administred as postexposure prophylaxis^477^
bamlanivimab and etesevimab	human IgG1 monoclonal antibodies against SARS-CoV-2 spike protein	EUA (2021/02/09)	none (recommended)	none	approved for emergency use (2021/05/13)	12-year-old or older COVID-19 patients with mild to moderate disease who are at high risk for progression to severe COVID-19 or as postexposure prophylaxis	intravenous infusion/single dose of 700 mg of bamlanivimab and 1400 mg of etesevimab	reduces the number of related hospitalizations or deaths and improves viral load reduction from baseline^484^
sotrovimab	recombinant human IgG1κ monoclonal antibody against SARS-CoV-2 spike protein	EUA (2021/05/26)	none (recommended)	special approval for emergency (2021/09/27)	approved for emergency use (2021/09/08)	12-year-old or older COVID-19 patients with mild to moderate disease who are at high risk for progression to severe COVID-19	intravenous infusion/single dose of 500 mg	reduces the risk of hospitalizations or deaths of any cause^494^
regdanvimab (Regkirona)	human IgG monoclonal antibody anti- SARS-CoV-2 spike protein	none	marketing authorization (2021/11/12)	none	approved for emergency use (2021/08/11)	adult COVID-19 patients with mild to moderate disease who are at high risk for progression to severe COVID-19	intravenous infusion/single dose of 40 mg/kg	accelerates viral clearance and clinical recovery^498^
baricitinib (Olumiant)	inhibitor of JAK1/JAK2 (anti-inflammatory effect) and AAK1 and GAK (endocytosis inhibitor)	EUA (2020/02/04)	none	special approval for emergency (2021/04/23)	approved for emergency use (2021/09/17)	hospitalized COVID-19 patients in need of supplemental oxygen	oral/4 mg per day for 14 days or until discharge	reduces mortality rates, intensive care unit admissions, requirement for invasive mechanical ventilation, and risk of serious adverse events^508^
Immunomodulatory Therapies								
tocilizumab (Actemra)	recombinant humanized monoclonal antibody against IL-6 receptor	EUA (2021/06/24)	none	none	none	hospitalized 2-year-old or older COVID-19 patients who are receiving systemic corticosteroids and require supplemental oxygen, noninvasive or invasive mechanical ventilation, or extracorporeal membrane oxygenation	intravenous infusion/single dose of 8 mg/kg (patients with weight ≥ 30 kg) or 12 mg/kg (patients with weight < 30 kg)	reduces risk of needing mechanical ventilation and poor outcome^520^

a Legend: FDA, U.S. Food and Drug Administration; EMA, European Medicines Agency (European Union); PMDA, Pharmaceuticals and Medical Devices Agency (Japan); ANVISA, Agência Nacional de Vigilância Sanitária (Brazil); EUA, Emergency Use Authorization; RdRp, RNA-dependent RNA polymerase; IgG, immunoglobulin G; JAK, Janus kinase; AAK1, AP2-associated kinase 1; GAK, cyclin G-associated kinase; IL-6, interleukin 6.

### Casirivimab and Imdevimab

The casirivimab and imdevimab cocktail (REGEN-COV or Ronapreve) (Regeneron Pharmaceuticals/Roche) was the first antibody-based therapy approved for COVID-19 by the FDA. Both are recombinant human IgG1 monoclonal antibodies that bind to the RBD of the SARS-CoV-2 S protein with high affinity, blocking S–ACE2 binding and thus preventing viral entry. They were discovered during a large antibody screen of genetically humanized mice that were challenged with SARS-CoV-2 S protein and by identifying antibodies from human COVID-19 survivors.^465^ Casirivimab and imdevimab bind to distinct and nonoverlapping regions of the RBD, and their combination (REGEN-COV) minimizes the escape of viral mutants that adapt to single-antibody-based treatments.^466−468^ REGEN-COV blocks the entry of important SARS-CoV-2 variants, such as B.1.1.7 (alpha), B.1.351 (beta), P.1 (gamma), and B.1.617.2 (delta), but the individual antibodies lose effectiveness over time, which supports the advantage of the dual-antibody treatment.^469−472^ COVID-19 animal models of mild (rhesus macaque) and severe (golden hamster) disease showed that REGEN-COV is effective in reducing viral loads in the upper and lower respiratory tracts, diminishing virus-induced pathology in rhesus macaque, and preventing weight loss in hamsters.^473^ A phase 3 randomized, double-blind, placebo-controlled trial was conducted with nonhospitalized patients above 18 years of age presenting at least one risk factor for developing severe COVID-19. After a single intravenous infusion of 2400 mg (1200 mg of each antibody) or 1200 mg (600 mg of each antibody) of the cocktail, REGEN-COV was shown to be safe and effective in reducing disease-related hospitalizations or deaths, time to symptoms resolution, time of hospital stay, and incidence of admission to an intensive care unit.^474^ Retrospective studies also showed that REGEN-COV reduces hospitalization rates in patients with mild to moderate disease in clinical practice.^475,476^ Another randomized, double-blind, placebo-controlled trial was conducted with asymptomatic subjects 12 years of age or older who were household contacts of a person infected with SARS-CoV-2. The study aimed to evaluate the capacity of a single subcutaneous injection of 1200 mg of REGEN-COV (600 mg of each antibody) to prevent SARS-CoV-2 infection. In exposed individuals, treatment reduced the risk of developing symptomatic or asymptomatic COVID-19, the average duration of symptoms, the average duration of RT-qPCR-detectable SARS-CoV-2 infection, and the viral load in exposed individuals.^477^ The clinical efficacy of REGEN-COV was maintained even in the presence of the SARS-CoV-2 delta variant.^478^ Different regulatory agencies approved REGEN-COV for treatment of people 12 years of age or older with mild to moderate COVID-19 who are at high risk for progression to severe disease and as prophylactic therapy for people exposed to SARS-CoV-2-infected individuals (Table 2).

### Bamlanivimab and Etesevimab

The bamlanivimab and etesevimab cocktail (Eli Lilly and Company) is composed of two human monoclonal antibodies identified from convalescent sera of different COVID-19 patients that bind to different but partially overlapping RBD regions of SARS-CoV-2 S protein and consequently block S-ACE2 binding and virus entry.^479,480^ Bamlanivimab (LY-CoV555) was identified from more than 400 antibodies and had the best neutralization capacity of different SARS-CoV-2 isolates. Preinfection treatment of rhesus macaques with bamlanivimab was able to reduce viral replication and viral loads in the upper and lower respiratory tracts.^479^ Etesevimab (CB6 or LY-CoV016) was also the best neutralizer of SARS-CoV-2 pseudoviruses and infectious viruses among the antibodies discovered concomitantly. Pre- and postinfection treatments of rhesus macaques with etesevimab reduced viral titers in the upper respiratory tract and diminished infection-related lung damage.^480^ The two antibodies have been tested alone or in association in clinical trials. Healthy adults tolerated a single intravenous infusion of etesevimab at 2.5 to 50 mg/kg.^481^ The blocking viral attachment and cell entry with SARS-CoV-2 neutralizing antibodies study (BLAZE) is a randomized, double-blind, placebo-controlled trial currently in progress to evaluate the efficacy of bamlanivimab monotherapy and bamlanivimab + etesevimab combined therapy for treating COVID-19 patients. Preliminary data evaluating bamlanivimab treatment alone in nonhospitalized patients with mild to moderate COVID-19 showed that after 11 days, patients who received a single intravenous infusion of 2800 mg presented a larger decrease in viral loads compared with patients who received a placebo. After 29 days, treatment with bamlanivimab was considered safe and was associated with reduced hospitalization and symptom severity.^482^ Results from phase 2/3 of the same trial that included the analysis of the combined therapy showed that a single intravenous infusion of 2800 mg of bamlanivimab plus 2800 mg of etesevimab generated a larger decrease in viral load after 11 days and lowered hospitalization rates after 29 days compared with a placebo.^483^ Among patients with mild to moderate COVID-19 and a high risk to develop severe disease, the combined therapy was able to reduce the number of COVID-19-related hospitalizations or deaths and reduced the viral load compared with the baseline at two different dosages: 2800 mg each^484^ and 700 mg of bamlanivimab plus 1400 mg of etesevimab.^485^ A retrospective study reported a reduction in the rate of hospital admissions or the need for supplementary oxygen in patients who received treatment with 700 mg of bamlanivimab and 1400 mg of etesevimab within 5 days of symptoms onset compared with patients who received the treatment after 5 days.^486^ Currently, the FDA authorizes bamlanivimab + etesevimab for emergency use to treat patients that 12 years of age or older with mild to moderate COVID-19 who are at high risk of progression to severe disease and as a prophylactic therapy (Table 2). However, different mutants of SARS-CoV-2 S protein have been reported to confer viral resistance to bamlanivimab, etesevimab and bamlanivimab + etesevimab therapies. Some of these mutations are found in important circulating SARS-CoV-2 variants, including B.1.351 (beta), P.1 (gamma), and B.1.617.2 (delta plus).^467,487−491^ For this reason, the combined therapy is no longer authorized for use in the U.S. in jurisdictions where the combined frequency of variants resistant to bamlanivimab and etesevimab exceeds 5% (https://www.phe.gov/emergency/events/COVID19/investigation-MCM/Bamlanivimab-etesevimab/Pages/resumption-in-distribution-bamlanivimabetesevimab.aspx).

### Sotrovimab

Sotrovimab (Vir Biotechnology/GlaxoSmithKline) is an engineered human monoclonal antibody derived from the S309 antibody identified from sera of a SARS-CoV-infected individual that is able to neutralize SARS-CoV, SARS-CoV-2 pseudoviruses and infectious viruses. S309 recognizes the S^B^ domain of the S protein, leading to noncompetitive inhibition of S–ACE2 binding and consequently virus entry. Fc-dependent effector mechanisms also play a role in S309 neutralization activity.^492^ Sotrovimab (VIR-7831) was obtained after modification of the S309 variable region for enhanced developability and the addition of an “LS” mutation in its Fc portion to confer extended half-life and enhanced distribution to the respiratory mucosa. Sotrovimab maintained S309 binding characteristics, effector mechanisms, and efficacy in neutralizing SARS-CoV-2. It was also capable of neutralizing important SARS-CoV-2 variants, including alpha, beta, gamma, delta, and kappa, with a significant shift in IC_50_ and IC_90_ values for only the alpha variant. A hamster model was used to test sotrovimab preinfection treatment *in vivo.* Sotrovimab reduced weight loss and viral loads when the dosage was ≥5 mg/kg and reduced viral titers in the lungs when the dosage was ≥0.5 mg/kg.^493^ A randomized, double-blind, controlled phase 3 trial has been evaluating the efficacy of a single intravenous infusion of 500 mg of sotrovimab in reducing hospitalizations and deaths in nonhospitalized and symptomatic adult COVID-19 patients with at least one risk factor for disease progression. Sotrovimab reduced 85% of the risk of hospitalization or death in the evaluated population.^494^ Sotrovimab is approved for emergency use by the FDA for treating patients 12 years of age or older with mild to moderate COVID-19 who are at high risk for progression to severe disease (Table 2).

### Regdanvimab

Celltrion Healthcare announced acceptance and priority review by Health Canada for its monoclonal antibody treatment for COVID-19, regdanvimab (originally CT-P59), a human monoclonal antibody obtained from sera of a convalescent COVID-19 patient. It binds to the RBD of SARS-CoV-2 S protein and competitively blocks S–ACE2 binding and consequently virus entry.^495^ Regdanvimab was able to inhibit SARS-CoV-2 infection *in vitro*.^496^ The antibody was tested in three different animal models: ferrets, golden Syrian hamsters, and rhesus monkeys.^495^ Postinfection treatment of ferrets with regdanvimab reduced viral titers in nasal washes and lungs, especially after administration of a high dosage (30 mg/kg). This reduction was followed by an improvement in clinical symptoms and lung pathology. In golden Syrian hamsters, postinfection treatment reduced viral titers in the lungs at all dosages tested, but with 30 mg/kg complete inhibition of SARS-CoV-2 replication was achieved. In rhesus monkeys, treatment with two doses of regdanvimab (45 and 90 mg/kg) reduced viral titers in nasal and throat swabs.^495^ Regdanvimab is effective against SARS-CoV-2 B.1.351 (beta), P.1 (gamma), and B.1.617. 2 (delta) variants *in vivo*, although *in vitro* assays have suggested different results.^496,497^ Two randomized, double-blind, placebo-controlled phase 1 studies have investigated the safety and efficacy of regdanvimab in a small cohort of healthy (study 1.1) or mildly symptomatic (study 1.2) adult COVID-19 patients that received a 10, 20, 40, or 80 mg/kg dose of the antibody in a single intravenous infusion. After a 90 day follow-up, no drug-related serious adverse event was detected in any patients who received therapy. Patients with high viral loads at baseline (>10^5^ copies/mL) who received 20, 40, or 80 mg/mL regdanvimab exhibited faster viral clearance than patients who received a placebo. Patients receiving regdanvimab also exhibited faster clinical recovery in a dose-dependent fashion. To date, phase 2 and 3 studies of regdanvimab efficacy are ongoing in nonhospitalized patients with SARS-CoV-2 infection.^498^ The European Medicines Agency (EMA) currently authorizes regdanvimab for COVID-19 treatment in adults with mild to moderate disease who are at high risk of developing severe outcomes (Table 2).

### Baricitinib

Baricitinib (Eli Lilly) is an anti-inflammatory drug inhibitor of Janus kinases 1 and 2 (JAK1/2) and is approved for treatment of rheumatoid arthritis.^499^ Baricitinib has been suggested as a possible antiviral drug for treatment of COVID-19 because of its capacity to inhibit the important clathrin-mediated endocytosis regulators AP2-associated protein kinase 1 (AAK1) and cyclin G-associated kinase (GAK), which would block viral entry through this route.^500^ In addition, the anti-inflammatory activity of this drug would be beneficial to COVID-19 patients because inflammation causes lung damage and subsequent mortality.^501^ In fact, exogenous treatment of blood cells from COVID-19 patients with baricitinib reduced SARS-CoV-2-specific immune response *in vitro*.^502^ Baricitinib was also able to inhibit IFN-α2-mediated transcriptome switch and ACE2 induction as well as SARS-CoV-2 replication in liver organoids but not in Vero and A549 cells, indicating a tissue-specific response.^503^ Although baricitinib was not effective in reducing SARS-CoV-2 loads in nasal/throat swabs and bronchoalveolar lavages of rhesus monkeys, daily oral administration of 4 mg for 8–9 days was able to reduce lung pathology and inflammation in infected animals.^504^ In a pilot study with adults presenting moderate COVID-19, baricitinib treatment was considered safe and did not lead to any serious adverse events in patients receiving 4 mg/day together with lopinavir–ritonavir therapy for 2 weeks. Clinical parameters and respiratory functions were improved, and discharges were higher in baricitinib-treated patients compared with patients receiving standard care (lopinavir–ritonavir plus hydroxychloroquine).^505^ Two randomized, double-blind, placebo-controlled phase 3 trials have evaluated the efficacy of oral baricitinib treatment at 4 mg/day for 14 days (or until discharge) in COVID-19 patients. The COV-BARRIER trial was conducted in hospitalized adult COVID-19 patients with at least one elevated inflammatory marker. In this clinical trial, baricitinib treatment resulted in lower mortality rates after 28 or 60 days and a similar frequency of serious adverse events compared with placebo.^506^ The ACTT-2 trial evaluated baricitinib treatment in combination with remdesivir in hospitalized moderate to severe COVID-19 patients. Baricitinib + remdesivir treatment resulted in a shorter recovery time, especially for patients receiving noninvasive ventilation or high-flow oxygen, and fewer serious adverse events than remdesivir alone.^507^ A recent systematic review and meta-analysis evaluating the clinical efficacy of baricitinib included the previously mentioned randomized clinical trials in addition to nonrandomized trials and observational studies. The authors concluded that baricitinib reduced mortality rates, intensive care unit admissions, and the requirement for invasive mechanical ventilation, improved the oxygenation index, and reduced the risk of serious adverse events.^508^ Baricitinib received an EUA from the FDA for treatment of COVID-19-hospitalized patients in need of supplemental oxygen (Table 2).

### Tocilizumab

Tocilizumab (Genentech/Roche) is a recombinant humanized monoclonal antibody directed against the IL-6 receptor (IL-6R) that was initially reported to inhibit IL-6-dependent multiple myeloma cell growth.^509^ It can bind both soluble and membrane-bound IL-6R, leading to the blockade of IL-6 signaling.^510^ The FDA approved the use of tocilizumab to treat adult patients with moderately to severely active rheumatoid arthritis or giant cell arteritis and to treat people 2 years of age or older with active polyarticular or systemic juvenile idiopathic arthritis or chimeric antigen receptor (CAR) T cell-induced cytokine release syndrome (https://www.accessdata.fda.gov/drugsatfda_docs/label/2017/125276s114lbl.pdf).^511−514^ Tocilizumab was proposed as a treatment option for severe COVID-19 cases at the beginning of the pandemic on the basis of evidence of its involvement during cytokine storms in disease progression.^515^ Gu and co-workers observed that in a mouse model the IL-6 pathway is important to initiate the cytokine storm syndrome in SARS-CoV-2-infected animals. They also showed that treatment with an anti-IL-6R antibody resulted in a reduction of lung neutrophil infiltration, supporting a benefit from anti-IL-6R therapy to COVID-19 patients.^516^ To date, three randomized, double-blind, placebo-controlled trials have been published evaluating the effect of tocilizumab treatment (8 mg/kg, single intravenous infusion) on hospitalized COVID-19 patients. The BACC Bay trial evaluating moderately ill patients did not find any benefit of tocilizumab treatment in preventing intubation, death, or other secondary/tertiary outcomes.^517^ The EMPACTA trial was conducted in hospitalized patients with COVID-19 pneumonia who were not receiving mechanical ventilation. In that trial, treatment with tocilizumab reduced the risk of receiving mechanical ventilation or death on day 28.^518^ In the COVACTA trial, treatment with tocilizumab did not improve the clinical status or mortality rates in patients with severe COVID-19 pneumonia.^519^ By analyzing data from open-label and double-blind randomized trials and cohort studies, Tleyjeh and co-workers concluded that tocilizumab treatment reduced the risk of mechanical ventilation and poor outcomes (randomized trials) and decreased the short-term mortality in COVID-19 patients.^520^ Tocilizumab received an EUA from the FDA for treatmento of hospitalized COVID-19 patients 2 years of age or older who are receiving systemic corticosteroids and require supplemental oxygen, noninvasive or invasive mechanical ventilation, or extracorporeal membrane oxygenation (Table 2).

## Promising COVID-19 Therapies

### Systemic Corticosteroids

Systemic corticosteroids have been widely used in COVID-19 patients and are recommended by WHO for treating patients with severe and critical illness.^521^ Corticosteroids are nonexpensive and broadly available molecules that diminish inflammation because of their capacity to stimulate the synthesis/release of anti-inflammatory mediators and to inhibit the synthesis/release of proinflammatory proteins through genomic and nongenomic pathways.^522^ Systemic inflammation is one of the main players in the development of severe outcomes in COVID-19, which suggested that anti-inflammatory drugs, such as corticosteroids, could be an effective treatment option for patients.^523^ Several observational studies and randomized controlled trials have evaluated the efficacy of systemic corticosteroids in COVID-19 patients.^521^ The CoDEX trial (randomized, open-label) evaluated dexamethasone treatment (10 or 20 mg daily for 5 days) of hospitalized moderate-to-severe COVID-19 patients receiving mechanical ventilation. In that trial, patients receiving dexamethasone showed longer ventilator-free periods than patients receiving standard care.^524^ The RECOVERY trial (randomized, open-label) evaluated dexamethasone treatment (6 mg daily for 10 days) of hospitalized moderate-to-severe COVID-19 patients. Patients receiving invasive mechanical ventilation or oxygen without invasive mechanical ventilation that were treated with dexamethasone showed lower mortality rates than those treated with standard care.^525^ Methylprednisolone is another corticosteroid used in COVID-19 treatment. The GLUCOCOVID trial (randomized, open-label) analyzed the efficacy of methylprednisolone in treating COVID-19 patients receiving oxygen without mechanical ventilation and showing evidence of systemic inflammatory response. Treatment with methylprednisolone (40 mg for 3 days followed by 20 mg for 3 days) reduced the risk of death, admission to the intensive care unit, or requirement for noninvasive ventilation.^526^ The Metcovid trial (randomized, double-blind, placebo-controlled phase 2b) evaluated the treatment of hospitalized COVID-19 patients with 0.5 mg/kg methylprednisolone *versus* placebo. Patients 60 years of age or older receiving methylprednisolone had a lower mortality rate compared with patients receiving placebo.^527^ A recent meta-analysis study including observational studies and randomized trials involving different drugs suggested that treatment with corticosteroids reduces mortality and risk of progression to invasive mechanical ventilation in severe COVID-19 patients.^528^ Treatment with methylprednisolone has resulted in better clinical outcomes than treatment with dexamethasone, but larger randomized controlled studies are needed to confirm this finding.^529,530^

### AZD7442

AZD7442 (AstraZeneca) is a cocktail of two human monoclonal antibodies previously designated COV2-2196 and COV2-2130 that after engineering were called AZD8895 and AZD1061, respectively. Both antibodies were obtained from sera of convalescent COVID-19 patients and are directed against SARS-CoV-2 S protein.^531^ They recognize nonoverlapping regions of the S RBD and can inhibit S–ACE2 binding and consequently neutralize SARS-CoV-2 in a synergistic manner. Prophylactic treatment of ACE2-mice with both antibodies (isolated or in combination) prevented SARS-CoV-2-induced weight loss, reduced viral loads in the lungs, heart, and spleen, diminished the expression of inflammation mediators in the lung, and reduced lung pathology.^532^ The combination of the two antibodies prevents mutational escape of SARS-CoV-2 variants, and the AZD7442 cocktail maintains its neutralization capacity even against different VOCs.^533^ Phase 3 clinical trials are currently being conducted to test AZD7442 for COVID-19 prevention (ClinicalTrials.gov ID: NCT04625725), postexposure prophylaxis (NCT04625972), outpatient treatment (NCT04723394 and NCT04518410), and inpatient treatment (NCT04315948 and NCT04501978). The company claims that pre-exposure prophylactic treatment with intramuscular AZD7442 (300 mg) reduced the risk of developing symptomatic COVID-19 by 77% (https://www.astrazeneca.com/media-centre/press-releases/2021/azd7442-prophylaxis-trial-met-primary-endpoint.html). Treatment of nonhospitalized patients who had been symptomatic for ≤7 days and ≤5 days with intramuscular AZD7442 (600 mg) reduced the risk of severe illness or death by 50% and 67%, respectively (https://www.astrazeneca.com/media-centre/press-releases/2021/azd7442-phiii-trial-positive-in-covid-outpatients.html). However, these clinical data have not been published to date.

### Molnupiravir

Molnupiravir (MK-4482, EIDD-2801) (Ridgeback Biotherapeutics/MSD) is a 5′-isopropyl ester of the nucleoside analogue *N*^4^-hydroxycytidine (NHC), a prodrug originally developed to treat influenza A and B viruses by the oral route. This drug has better oral bioavailability in non-human primates and ferrets than its precursor NHC and is efficiently hydrolyzed *in vivo* after absorption, releasing the active molecule in the plasma.^534^ NHC is capable of inhibiting SARS-CoV-2 and other related coronaviruses using *in vitro* models.^535,536^ This nucleoside analogue is incorporated by the viral RdRp into the nascent viral RNA, leading to error catastrophe and RNA synthesis inhibition.^537^ When administered to Syrian hamsters and human lung-only mice before or after SARS-CoV-2 infection, molnupiravir was able to decrease viral loads in the lungs and lung pathology.^535,538^ In ferrets, molnupiravir treatment after SARS-CoV-2 infection reduced viral loads in nasal lavages and inhibited the spread to untreated contact animals.^539^ Molnupiravir was also able to inhibit SARS-CoV-2 infection in hamsters infected with the B.1.1.7 (alpha) and B.1.351 (beta) variants of the Wuhan virus.^540^ A randomized, double-blind, placebo-controlled phase 1 trial with healthy volunteers attested that oral administration of 50–1600 mg of molnupiravir was well-tolerated and that only a few mild adverse events were observed in the treated population.^541^ Phase 3 clinical trials are currently being conducted to test molnupiravir for COVID-19 postexposure prophylaxis (NCT04939428), outpatient treatment (NCT04575584), and inpatient treatment (NCT04575597). MSD and Ridgeback Biotherapeutics announced that molnupiravir reduced the risk of hospitalization or death by approximately 50% in nonhospitalized adult patients with mild-to-moderate COVID-19 disease (https://www.merck.com/news/merck-and-ridgebacks-investigational-oral-antiviral-molnupiravir-reduced-the-risk-of-hospitalization-or-death-by-approximately-50-percent-compared-to-placebo-for-patients-with-mild-or-moderat/). Recently, the U.K.’s Medicines and Healthcare Products Regulatory Agency was the first agency to temporary authorize molnupiravir for the treatment of mild to moderate COVID-19 in adults with at least one risk factor for severe illness (https://www.gov.uk/government/publications/regulatory-approval-of-lagevrio-molnupiravir).

### PF-07321332

PF-07321332 (Paxlovid) (Pfizer Inc.) is a reversible covalent inhibitor of the 3CL^pro^ protease of SARS-CoV-2.^542^ It is a second-generation orally available 3CL^pro^ inhibitor developed by Pfizer during the pandemic. To date, there are three clinical trials evaluating the association of PF-07321332 and ritonavir for COVID-19 treatment of patients with low risk (NCT05011513) or high risk (NCT04960202) to develop severe illness and as postexposure prophylaxis (NCT05047601). Pfizer recently announced that PF-07321332 combined with ritonavir significantly reduced by 89% hospitalization and death of nonhospitalized adult patients with COVID-19 who are at high risk of progressing to severe illness (https://www.pfizer.com/news/press-release/press-release-detail/pfizers-novel-covid-19-oral-antiviral-treatment-candidate). Ritonavir is coadministered in low doses to reduce metabolism of PF-07321332.

## Prevention

At present, vaccination represents the most effective long-term strategy for controlling and preventing COVID-19.^111^ Besides effective vaccines, the prophylaxis of SARS-CoV-2 infection is based on a series of countermeasures. Reducing the spread of COVID-19 requires two factors: reducing the transmission probability per contact and limiting contact between persons *via* physical distancing.^543,544^ Thus, prevention practices together with the implementation of effective measures represent a crucial strategy to contain the rapid spread of SARS-CoV-2 among humans.

Recommendations to prevent SARS-CoV-2 infection established by the CDC include the following: (i) Masks should be worn, as they reduce transmissibility through exhaled air from infected respiratory particles in both clinical and laboratory scenarios and subsequently could result in a large reduction in the risk of SARS-CoV-2 infection at the population level.^125,543,545^ (ii) The 2 meter social distancing rule should be applied. Inside the home, close contact with people who are sick should be avoided. When outside, a distance of 2 meters from other people should be maintained. (iii) Crowds and poorly ventilated areas should be avoided. (iv) Hands should be washed often with soap and water for at least 20 seconds, especially after staying in a public place or after blowing the nose, coughing, or sneezing. If soap and water are not readily available, a hand sanitizer that contains at least 60% alcohol should be used. Eyes, nose, and mouth should not be touched with unwashed hands. (v) Coughs and sneezes should be covered. Those wearing a mask can cough or sneeze into the mask. If a mask is not being worn, the mouth and nose should be covered with a tissue during coughing or sneezing, or the inside of the elbow should be used without spitting. (vi) Highly touched surfaces should be cleaned and disinfected daily, including handles, desks, phones, keyboards, toilets, faucets, tables, doorknobs, light switches, countertops, and sinks. (vii) Health should be monitored daily.^546^ Notably, these measures are most effective at reducing the spread of the virus when adherence is high among the human population.^545^ For healthcare and frontline professionals, additional precautions are required, including airborne precautions (use of N95 respirators), contact precautions (use of gown and gloves), and eye protection (use of goggles or a face shield).^547^ Additionally, early diagnosis, quarantine, and supportive treatments are essential for the clinical management of infected patients.

SARS-CoV-2 can remain for a long time on various types of surfaces, including aerosols, plastic, stainless steel, copper, and cardboard,^128,129^ so several agents can be used to inactivate the virus.^547^ In this context, Chin and colleagues reported the stability of SARS-CoV-2 when exposed to several disinfectants agents such as household bleach (1:49–1:99), hand soap solution (1:49), ethanol (70%), povidone iodine (7.5%), chloroxylenol (0.05%), chlorhexidine (0.05%), and benzalkonium chloride (0.1%).^127^ These agents were evaluated at different times of exposition (5, 15, and 30 min) followed by a 50% tissue culture infective dose (TCID_50_) assay for viral titration to confirm the presence of infectious viruses. The results revealed that viral inactivation was observed after only 5 min of exposure using all of the disinfectant agents except for hand soap.^127^ In addition, it was shown that SARS-CoV-2 is stable at a wide range of pH values (3–10) at room temperature.^127^

### Vaccines

**Figure 11:**
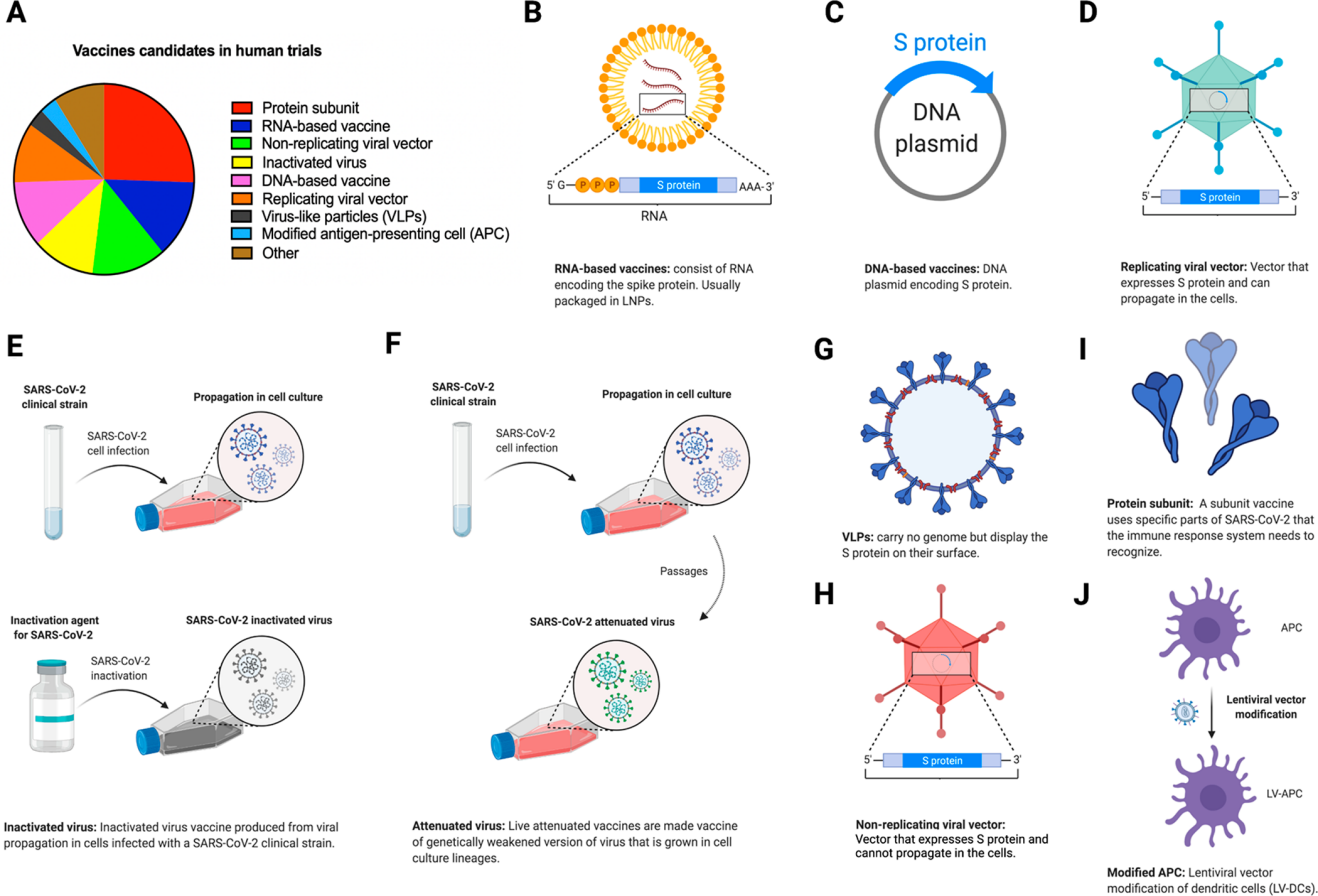
Vaccine platforms currently being developed against SARS-CoV-2 infection. Since the emergence of SARS-CoV-2, great efforts have been made by research groups and companies around the world toward the development of effective vaccines. Briefly, the graph in (A) shows current vaccine candidates in the clinical phase. Vaccine platforms currently being developed against SARS-CoV-2 infection include those based on (B) RNA, (C) DNA, (D) replicating viral vectors, (E) inactivated viruses, (F) attenuated viruses, (G) viruslike particles (VLPs), (H) nonreplicating viral vectors, (I) protein subunits, and (J) modified antigen-presenting cells (APCs). Abbreviations: S, spike protein; LNP, lipid nanoparticle; LV, lentiviral vector; DC, dendritric cell. This figure was created with Biorender.com.

As for other infectious diseases, vaccination is the main approach to preventing COVID-19. Since the emergency of SARS-CoV-2, several vaccine platforms have been developed, and as of July 14, 2022, 40 vaccines have been approved by at least one country in the world (Figure 11). There are 153 vaccines in clinical development and 196 in preclinical development. Currently approved vaccines are based on protein subunits (*n* = 16), inactivated virus (*n* = 11), nonreplicated viral vectors (*n* = 7), RNA (*n* = 4), DNA (*n* = 1), or viruslike particles (VLPs) (*n* = 1) (https://covid19.trackvaccines.org/vaccines/approved/). Among these, 10 vaccines were granted Emergency Use Listing (EUL) by the WHO, and they are discussed below.^548^

Vaccines based on protein subunits consist of antigenic pathogen fragments and have the potential to exhibit efficacy in protecting humans from viral infection.^549^ However, because only a few viral fragments are included, they do not display the full antigenic complexity of the virus and are therefore of limited value because the protective efficacy can be reduced.^550^ Protein subunit vaccines, such as Nuvaxovid (Novavax) and COVOVAX (manufactured by Serum Institute of India, Novavax formulation), are based on the recombinant nanoparticle S protein associated with Matrix-M adjuvant. Stabilizing mutations have been introduced into the S protein that are intended to prevent the intrinsic problem of its conformational instability.^551^

Inactivated vaccines such as Covaxin (Bharat Biotech), Covilo (Sinopharm), and CoronaVac (Sinovac) are based on whole-virus preparations made in cells followed by chemical inactivation, purification, and mixing with specific compounds that act as stimulants of immune cells and amplifiers of immune responses, such as aluminum hydroxide adjuvant.^552^ Notably, chemically inactivated, irradiated, or heat-inactivated pathogens sometimes lose their immunogenicity, rendering this platform less efficient than live attenuated pathogen platforms.^552^

Nonreplicated viral vector vaccines approved for human use are based on either animal or human replication-defective adenovirus vectors. Vaxzevria (Oxford/AstraZeneca) and Covishield (manufactured with Oxford and AstraZeneca formulation by Serum Institute of India and Fiocruz-Brazil) are based on chimpanzee adenovirus encoding the SARS-CoV-2 S glycoprotein. Ad26.COV2.S (Janssen/Johnson & Johnson) is based on a replication-incompetent recombinant human adenovirus type 26 vector expressing the S protein in a stabilized conformation.^553^

RNA-based vaccines have been approved for the first time for human use, and they have displayed excellent safety and efficacy profiles, placing this platform at the forefront of the fast development of vaccines against emerging diseases.^554−556^ Although there are some differences in how they were engineered, Comirnaty (Pfizer/BioNTech) and Spikevax (Moderna) are both lipid-nanoparticle-formulated, nucleoside-modified RNA vaccines that encode full-length SARS-CoV-2 S protein modified by two proline mutations to ensure that it remains in the prefusion conformation.

A recent systematic review and meta-regression on COVID-19 vaccine efficacy and/or effectiveness concluded that most vaccines (81%) had efficacy or effectiveness against severe disease that remained greater than 70% after full vaccination with a minimal decrease (∼10%) 6 months after immunization.^557^ Most of these vaccines have been manufactured on the basis of the prototype Wuhan-Hu-1 strain, and their efficacy and effectiveness are lower toward the VOCs that have emerged since the beginning of the pandemic. Thus, updates of vaccine composition to reflect the current most prevalent variant(s) of SARS-CoV-2 must be considered to provide optimum protection against these circulating SARS-CoV-2 variants. With the ephemeral nature of COVID-19 vaccine-induced immunity, novel prophylactic approaches that elicit long-term protection are warranted.

## Emerging Variants of SARS-CoV-2

Since the discovery of SARS-CoV-2 in December 2019, the viral genomes from clinical samples have been sequenced daily and worldwide, and thousands of complete genomes have been deposited in databanks to date. RNA viruses, such as SARS-CoV-2, are known to present high mutation rates due to the low capacity of the RdRp to correct errors during genome replication. However, the *Coronaviridae* family is an exception since the replication machinery of these viruses contains a 3′-5′ exoribonuclease domain that is able to proofread.^558^ This domain has also been detected in SARS-CoV-2 as a component of the nonstructural protein nsp14.^559^ In fact, following analysis of different genomes published through November 2020, SARS-CoV-2 showed low global nucleotide diversity. However, nucleotide diversity tends to increase as virus incidence enhances.^560^ Virus mutation over time has culminated in the emergence of SARS-CoV-2 variants, *i.e.*, virus specimens that are genetically different from the main or initial SARS-CoV-2 lineage. These mutations could be phenotypically neutral, with no major impacts on viral biology, or confer advantages to the variants that possess them, improving viral adaptation and fitness.^561^ The first important variation of the Wuhan reference strain observed possessed the D614G mutation on the S protein. This mutation was first detected in March 2020 and rapidly became globally dominant, being present in most of the current circulating viral lineages. D614G confers replicative advantages to the virus that could explain its rapid dissemination worldwide.^562,563^

**Figure 12:**
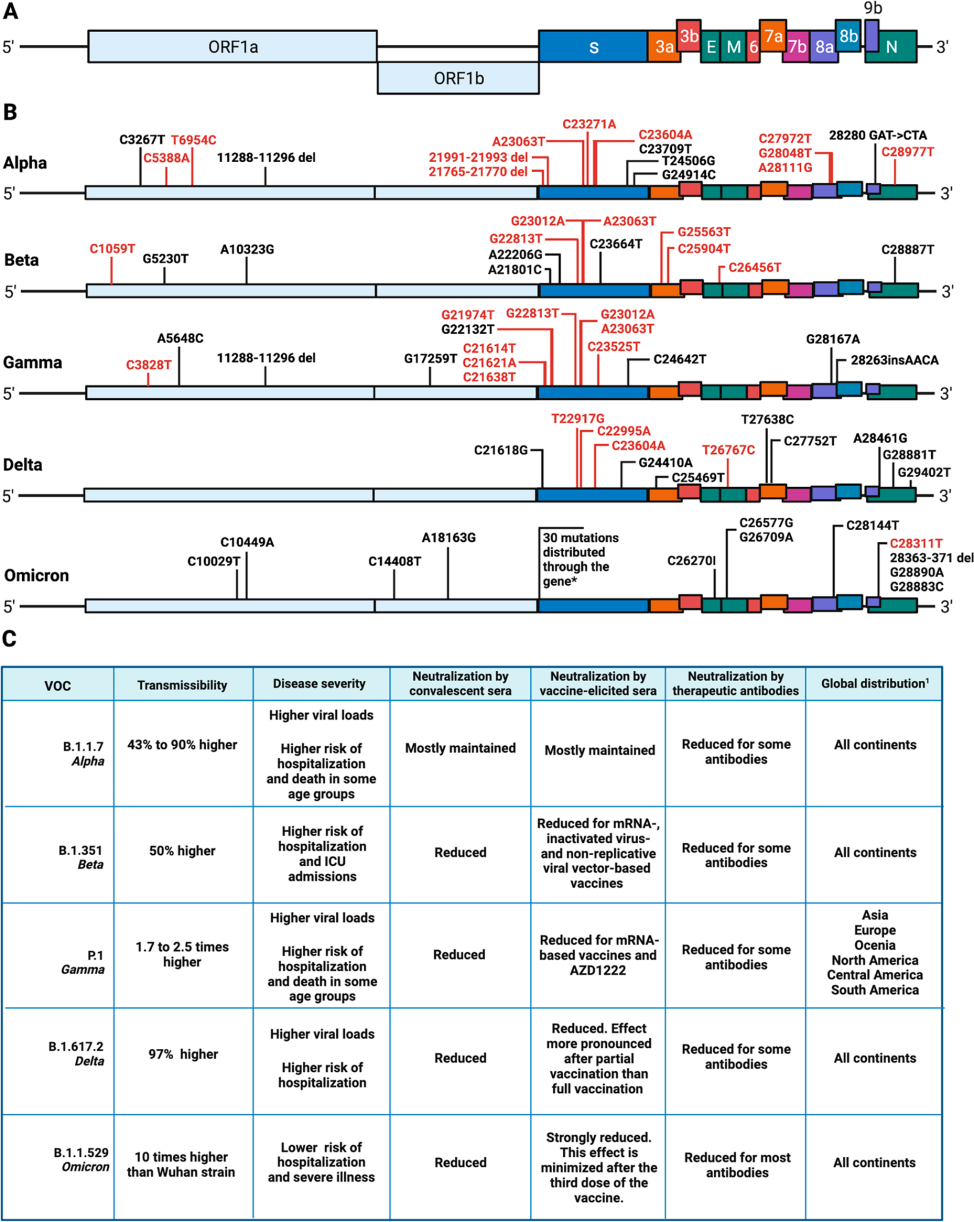
SARS-CoV-2 variants of concern (VOCs). (A) Schematic of the SARS-CoV-2 genome architecture. (B) Definition of nonsynonymous mutations and deletions of each VOC. Nucleotide alterations without predicted or confirmed impact on protein structure and/or function are shown in black. Nucleotide alterations with predicted or confirmed impact on protein structure and/or function are shown in red. (C) Phenotypic characteristics of VOCs. Global distributions are according to the PANGO lineages website (https://cov-lineages.org/index.html). This figure was created with Biorender.com.

The CDC has classified SARS-CoV-2 variants into three groups: variants of interest (VOIs); VOCs, and variants of high consequence (VOHCs). VOIs are variants with limited prevalence or dissemination that possess mutations predicted to affect the transmission, diagnostics, therapeutics, or sensibility to antibodies produced after previous exposition or vaccination. VOCs are defined as those who have evidence of increased incidence/transmission, diagnostic or therapeutic failure, or reduced neutralization by antibodies produced after previous exposition or vaccination. VOHCs are those for which prevention measures or medical countermeasures have reduced effectiveness. As the COVID-19 pandemic evolved, SARS-CoV-2 has been characterized by the repeated identification of different variants over time.^564^ Since its emergence, five SARS-CoV-2 variants have been classified as VOCs (alpha, beta, gamma, delta, and omicron) at different times during the course of the pandemic.^354,361,564^ To date, CDC has not classified any variant as a VOHC. All of these variants share the D614G mutation, which is involved in increased virus replication in the upper respiratory tract and higher transmissibility.^361,563^ There are different schemes for variant naming,^565,566^ but here we will use the nomenclature based on the phylogenetic framework proposed by Rambaut and colleagues, known as the Pango lineage,^36^ and the label defined by the World Health Organization (https://www.who.int/en/activities/tracking-SARS-CoV-2-variants/). The VOCs will be discussed more deeply in the sections below and are summarized in Figure 12.

### Alpha

In December 2020, after epidemiological and genomic surveillance, the U.K. reported a new SARS-CoV-2 variant, initially called VUI-202012/01 (first variant under investigation in December 2020).^567^ This variant, renamed B.1.1.7 according to Pango lineages and subsequently as alpha according to WHO, genetically differs from others by 23 nucleotide alterations: 14 nonsynonymous substitutions, six synonymous substitutions, and three deletions (Table S1).^568^ The alpha variant was estimated to be more transmissible than previous variants, becoming the most prevalent in the U.K. over time and associated with higher mortality.^569,570^ The reproduction number of the alpha variant is 43% to 90% higher than those of previous variants in the U.K. and other countries, depending on the model used to calculate the data.^569^ Recent studies have proposed that the enhanced transmissibility could be related to higher viral loads or a longer infectious period.^569^ In fact, patients infected with the alpha variant have presented higher viral RNA levels and a more lasting positivity for the virus.^571,572^ Some commercial RT-qPCR kits targeting the S gene could not detect infection with the alpha variant because of the presence of the 21765–21770 deletion in the viral genome, a phenomenon usually called S gene target failure (SGTF).^573^ Using the SGTF as an approach to differentiate the alpha variant from others, Funk and colleagues observed a higher risk of hospitalization of European patients in the age groups of 20–39 and 40–59 years and ICU admission in the age group of 40–59 years.^574^ Recent studies have shown that the alpha variant was associated with a high risk (55–64%) of death in infected patients with SARS-CoV-2.^570,575^ Mutations on the S protein of the alpha variant have been linked to reduced neutralization by monoclonal antibodies.^576,577^ However, their impact on viral neutralization by convalescent sera or sera obtained from vaccinated subjects seems to be small or absent.^576−579^ The efficiencies of the mRNA-based vaccines BNT162b2 (BioNTech, Pfizer) and mRNA-1273 (Moderna) against the alpha VOC were shown to be similar to those against the previous variant.^580,581^ The inactivated-virus-based vaccines BBIBP-CorV (Sinopharm) and BBV152/COVAXIN (Bharat Biotech) were shown to be effective against alpha,^582,583^ while CoronaVac (Sinovac) reduced the neutralization capacity against this variant by a factor of 0.5.^583^ The nonreplicative-viral-vector-based vaccines ChAdOx1 nCoV-19/AZD1222 (Oxford, AstraZeneca) and Ad26.COV2.S (Janssen) also showed a reduced neutralization capacity against the alpha variant^584^ (Assessment Report EMA/158424/2021), although the overall efficacy of AZD1222 against symptomatic and asymptomatic cases was preserved (61.7% against the alpha variant and 77.3% against other variants).^584^ The efficacy of Ad26.COV2.S in preventing COVID-19 caused by the alpha variant still needs to be evaluated. Sputnik V Ad26/Ad5 (Gamaleya Institute) is still effective in neutralizing the alpha variant.^585^

### Beta

In the same month that the alpha variant was first reported in the U.K., South African researchers described a new variant of SARS-CoV-2 that emerged in the country after the first epidemic wave.^586^ Initially called S501.V2, this new variant was named B.1.351 by Pango lineages and beta according to the WHO.^36^ The beta VOC was first characterized as containing 31 mutations, of which four are shared with the parent B.1 variant. Among the 27 specific variations found in this lineage, 21 are nonsynonymous mutations while 12 have been fixed in the variant population over time (Table S1).^586^ This emerging variant shares with the alpha VOC the N501Y substitution on the S protein, an important mutation for virus phenotype. It was estimated that beta VOC was 50% more transmissible than previously circulating variants.^587^ Compared with non-VOCs, the beta VOC showed a higher risk of hospitalization in European patients in the age groups of 40–59 and 60–79 years and ICU admission in the age group of 40–59 years, but this did not lead to an increase in the mortality rate.^574^ Until now, what seems to be the most important characteristic of beta VOC is its reduced sensitivity to neutralization by convalescent and vaccine-elicited sera.^577,578,583,588−591^ The BNT162b2, mRNA-1273, BBIBP-CorV, CoronaVac, ChAdOx1 nCoV-19/AZD1222, and Sputnik V Ad26/Ad5 vaccines showed reduced neutralization capacity against this variant.^577,578,583,585,588−592^ In a population-based study, an important reduction in the vaccine efficacy was also observed for ChAdOx1 nCoV-19/AZD1222.^592^ BNT162b2, for instance, seems to maintain its efficacy to prevent severe forms of the disease.^580^ BBV152/COVAXIN and Ad26.COV2.S were estimated to be effective against the beta VOC^593^ (Assessment Report EMA/158424/2021). Reduced neutralization by therapeutic monoclonal antibodies was also observed for this variant.^578,591^ Thus, the beta VOC could be implicated in the augmentation of reinfection frequency and vaccine or therapy failure and must be closely tracked by genomic surveillance.

### Gamma

In December 2020, a new SARS-CoV-2 variant called P.1 (gamma) was detected in Manaus, Brazil, and was potentially linked to an important increase in COVID-19 frequency in that city.^594,595^ The same variant was also detected in infected travelers who originated from that state and arrived in Tokyo, Japan, in January 2021.^596^ This new variant was originally characterized by 35 mutations distributed across the entire genome. Ten nonsynonymous variations are located in the S gene, of which three (K417T, E484 K and N501Y) are shared with the B.1.351 variant and one (N501Y) is shared with both the B.1.1.7 and B.1.351 variants (Table S1).^594^ The transmissibility of the gamma VOC was estimated to be 1.7 to 2.5 times higher than those of non-gamma variants circulating in Manaus, which made it the dominant variant in the city in January 2021.^594,595^ Higher viral loads in people infected with the gamma variant were also reported, which could be a contributing factor to its more infectious behavior.^595^ Infection with the gamma variant was associated with a high risk of hospitalization and ICU admission.^574^ Reinfection cases^597,598^ and resurgence of the disease in places where herd immunity was probably achieved by previous variants^354,599^ could also be explained by the rise of this new variant. Gamma is little to completely resistant to neutralization by therapeutic monoclonal antibodies and convalescent plasma.^600,601^ The BNT162b2 and mRNA-1273 vaccines were the best assessed, showing modest to moderate reductions in their neutralization capacities against this variant.^589,600−602^ In fact, a case of a fully BNT162b2-vaccinated man that developed mild symptoms after gamma infection was reported.^603^ CoronaVac was estimated to be effective against gamma,^604^ and the capacity of AZD1222 to neutralize this virus is reduced.^602^

### Delta

In December 2020 and the first months of 2021, India reported an increase in COVID-19 cases associated with the emergence of the new SARS-CoV-2 variants B.1.617.1, B.1.617.2, and B.1.617.3 which were exported to other countries by Indian travelers.^605^ B.1.617.2 (delta) rapidly became the dominant lineage in India and established a community transmission chain in other countries worldwide, rapidly increasing its proportion.^605,606^ With a reproduction number 97% higher than the observed number for non-VOCs and at least 30% higher than those for other VOCs, the delta VOC was estimated to become the dominant circulating lineage worldwide until the emergence of the omicron variant.^606^ Twelve nonsynonymous mutations characterize this variant, five of them being in the S gene (Table S1). The increased transmissibility presented by delta could be related to higher viral loads,^607−609^ possibly due to a higher replication rate compared with other variants.^610^ Also, two spike mutations presented by this variant, L452R and T478K, are predicted to improve its interaction with ACE2 and possibly increase the ability of the virus to enter human cells,^611,612^ although this hypothesis should be further evaluated. The delta VOC was linked to a higher risk of hospitalization and disease severity.^607,613^ As observed for other VOCs, the delta VOC is resistant to neutralization by some therapeutic monoclonal antibodies^578,614^ and to convalescent sera.^578,614^ BNT162b2-vaccine-elicited sera also showed a reduced neutralization capacity against delta, especially after partial vaccination.^578,614−617^ Full vaccination with BNT162b2 seems to generate immunity against delta comparable to that against other SARS-CoV-2 variants.^578,614,617^ In fact, full vaccination had a similar efficacy against the delta VOC compared to the B.1.1.7 variant in population-based studies.^618,619^ mRNA-1273 vaccination against the development of symptomatic cases derived from delta infection was less effective than that from B.1.1.7 infection.^619^ However, mRNA-based vaccines (BNT162b2 and mRNA-1273 analyzed together) were able to protect vaccinated subjects from the development of moderate to severe illness.^620^ Inactivated-virus-based vaccines, including HB02 and WIV04 from Sinopharm, CoronaVac, and Biokangtai’s inactivated COVID19 vaccine, were evaluated together regarding their efficacy against the delta VOC in a Chinese population. They achieved efficacies of 69.5% against COVID-19-associated pneumonia and 100% against severe illness.^621^ Neutralization of the delta variant by BBV152/COVAXIN-elicited sera was slightly reduced, which suggests that these sera maintained their efficacy against this strain.^622^ Sera elicited by nonreplicative-viral-vector-based vaccines had their neutralization capacity reduced against the delta VOC.^578,614,623,624^ The efficacy of AZD122 obtained from population-based studies diverges between authors and must be further investigated.^618,619^

### Omicron

The SARS-CoV-2 omicron variant (B.1.1.529) first emerged in Botswana and South Africa and has been associated with a steep increase in the incidence of COVID-19; it was classified as a VOC by the WHO on November 26, 2021. Its emergence has contributed to the fourth wave of the COVID-19 pandemic in many countries worldwide.^625^ The omicron VOC displays distinct biological characteristics, including strong binding to human ACE2 receptor and high transmissibility,^626−628^ despite being less virulent than the original strain or the previous VOCs. In addition, the omicron VOC displays high environmental stability and high resistance against clinically approved monoclonal antibodies and immunity elicited by natural infection or vaccination.^629−631^ More recently, we have revised in detail the SARS-CoV-2 omicron variant and several characteristics related to this novel variant.^632^

## Final Considerations and Public Health Perspectives

The emergence of this novel coronavirus in the human population precipitated a global threat and was designated a pandemic by the WHO on March 11, 2020. Two years later, it can certainly be considered one of the greatest public health crises. Notably, global society was unprepared for the emergence of SARS-CoV-2 and the serious and widespread consequences of COVID-19 infections. However, the rapid response to the COVID-19 crisis, including efforts made by the WHO, health authorities, industry, governments, and global researchers, have improved public health resilience and helped to mitigate societal impacts. Clinically these actions included the open sharing of information on infection rates and deaths. Open research, like the early publication of the viral genome, and industry engagement with the development and patient trial validation of vaccine candidates as well as governments’ rapid approval of novel diagnostic tests and vaccines—all within a short time—helped to blunt the severity of SARS-CoV-2. The pandemic has highlighted both our ability to respond and our shortcomings in pandemic preparedness for events of this scale.

The pandemic has taught several lessons, including the need for rapid large-scale production and distribution of vaccines, the production and availability of point-of-care diagnostic tests, and last but not least, the need to address the trade of wildlife and ecosystem loss as critical factors in the emergence of infectious diseases. The lessons learned from the COVID-19 pandemic will be critical for dealing with future public health threats, especially for the emergence of new pathogens.

In addition to seeing so much scientific knowledge generated during the last 2 years, society also many experienced lessons. During the course of the COVID-19 pandemic, the human population showed incredible resilience in the face of losses and economic uncertainty. We are still learning to live with SARS-CoV-2, but we are certainly more prepared for future biothreats, and there is reason to hope that human ingenuity will ensure that this virus will not be a threat over time.

## Supporting Information Available

Definitions of nonsynonymous mutations and deletions of each variant of concern of SARS-CoV-2 classified by the U.S. CDC and their individual impacts on the virus phenotype (Table S1) (PDF)
