# Supplementary material for: Two Years into the COVID-19 Pandemic: Lessons Learned
Source: ACS Infect Dis. 2022 Aug 8;8(9):1758–814. doi: 10.1021/acsinfecdis.2c00204 (PMC9380879; doi:10.1021/acsinfecdis.2c00204)
Supplement: Supplementary file 1 — id2c00204_si_001.pdf [file id2c00204_si_001.pdf]

Supporting Information

Two Years into the COVID-19 Pandemic: Lessons Learned

Severino Jefferson Ribeiro da Silva[1,2*], Jessica Catarine Frutuoso do Nascimento[1], Renata Pessôa Germano Mendes[1], Klarissa Miranda Guarines[1], Caroline Targino Alves da Silva[1], Poliana Gomes da Silva[1], Jurandy Júnior Ferraz de Magalhães[1,3,4,5], Justin R.J. Vigar[2], Abelardo Silva-Júnior[6], Alain Kohl[7], Keith Pardee[2,8] and Lindomar Pena[1*]

[1]Laboratory of Virology and Experimental Therapy (LAVITE), Department of Virology, Aggeu Magalhães Institute (IAM), Oswaldo Cruz Foundation (Fiocruz), 50670-420, Recife, Pernambuco, Brazil;

[2]Department of Pharmaceutical Sciences, Leslie Dan Faculty of Pharmacy, University of Toronto, Toronto, ON M5S 3M2, Canada;

[3]Department of Virology, Pernambuco State Central Laboratory (LACEN/PE), 52171-011, Recife, Pernambuco, Brazil;

[4]University of Pernambuco (UPE), Serra Talhada Campus, 56909-335, Serra Talhada, Pernambuco, Brazil;

[5]Public Health Laboratory of the XI Regional Health, 56912-160, Serra Talhada, Pernambuco, Brazil;

[6]Institute of Biological and Health Sciences, Federal University of Alagoas (UFAL), 57072-900, Maceió, Alagoas, Brazil;

[7]MRC-University of Glasgow Centre for Virus Research, Glasgow, Scotland, G61 1QH, United Kingdom;

[8]Department of Mechanical and Industrial Engineering, University of Toronto, ON M5S 3G8, Toronto, Canada;

*Corresponding authors:

Severino Jefferson Ribeiro da Silva, Ph.D. Email: jefferson.silva@utoronto.ca / jeffersonbiotecviro@gmail.com

Lindomar Pena, Ph.D. Email: lindomar.pena@fiocruz.br

Table S1: Defining non-synonymous mutations and deletions of each variant of concern of SARS-CoV-2 classified by CDC-USA, and their individual impact on virus phenotype.

PANGO Lineage/ WHO designation	Gene	Codified protein	Nucleotide alteration	Amino acid alteration	Mutation impact	Variant characteristics	Reference
B.1.1.7 / Alpha	*ORF1ab*	NSP3	C3267T	T1001I	NF	NF	NF
			C5388A	A1708D	Decrease in CD8+ T cell activation after epitope presentation by HLA-A2 (E). Molecular docking revealed a slight decrease in the interaction similarity of mutant peptides to HLA-A2 (P)	Possible immune evasion	[1]
			T6954C	I2230T	Decrease in CD8+ T cell activation after epitope presentation by HLA-A2 (E). Molecular docking revealed a slight decrease in the interaction similarity of mutant peptides to HLA-A2 (P). Disturbs the N-terminal hydrophobic amino acid sequence of the protein, preferred binding motif for DRB1*04:04 (P)	Possible immune evasion	[1, 2]
		NSP6	11288-11296 del	SGF 3675-3677 del	NF	NF	NF
	S	Spike protein	21765-21770 del	HV 69-70 del	Absent amplification of gene S in RT-qPCR using the TaqPath COVID-19 RT-qPCR kit (E)	Diagnostic failure	[3]
			21991-21993 del	Y144 del	Impairment of neutralizing antibody ligation	Possible immune evasion	[4]
			A23063T	N501Y	Favored open state of Spike protein, conformation recognized by ACE2 (P); increased binding affinity of RBD to ACE2 (P, E); increased virus reproduction number (E); augment in RBD/ACE2 binding, increased reproduction fitness in the upper airway of Syrian hamsters and higher replication in Vero and human airway epithelial cell lines (E); increased binding affinity of RBD to GRP78 (P)	Possible increase in infectivity and transmissibility	[5] [6] [7] [8] [9] [10]
					Reduced neutralization by monoclonal antibodies and polyclonal sera (E)	Possible immune evasion	[9] [11]
			C23271A	A570D	Possible alteration in the balance of closed to open conformation of Spike protein (S)	NF	[12]

			C23604A	P681H	Increased furin-mediated cleavage of the Spike protein, with no effect on viral entry in Vero cells and nor on viral replication in primary human airway epithelial (P, E)	NF	13
			C23709T	T716I	NF	NF	NF
			T24506G	S982A	NF	NF	NF
			G24914C	D1118H	NF	NF	NF
	Orf8	ORF8	C27972T	Q27stop	Premature truncation that could lead to the codification of two different chains, orf8a and orf8b. Possible impairment of the homodimerization site, reducing the immunogenic potential of the protein (P)	Possible immune evasion	14
			G28048T	R52I	Localized in the dimerization interface. Slight reducing in the dimer interaction affinity (P)	Possible immune evasion	15
			A28111G	Y73C	Disturbs the N-terminal hydrophobic amino acid sequence of the protein, preferred binding motif for DRB1*04:04 (P)	Possible immune evasion	2
	N	Nucleocapsid protein	28280 GAT->CTA	D3L	NF	NF	NF
			C28977T	S235F	Stabilizes the N protein (P)	NF	15
B.1.351 / Beta	*ORF1ab*	NSP2	C1059T	T265I	Possibly addition of a sheet structure at position 266 in the NSP2 domain (P)	NF	16
		NSP3	G5230T	K1655N	NF	NF	NF
		NSP5	A10323G	K3353R	NF	NF	NF
	S	Spike protein	A21801C	D80A	NF	NF	NF
			A22206G	D215G*	NF	NF	NF
			G22813T	K417N	Higher infectivity in cell models, alone or in combination with other B.1.351 spike mutations (E); increased binding affinity of RBD to ACE2 (P); greater spike S1-S2 cleavage (E); increased cell-to-cell fusion (E)	Possible increase in infectivity and transmissibility	17 18 19 20
					Together with E484K, lead to a moderate resistance to neutralization by convalescent sera (E); together with E484K and N501Y lead to a high resistance to neutralization by vaccine-elicited sera (E)	Possible immune evasion	21 18

					Escape from neutralization by therapeutic monoclonal antibodies (E)	Possible therapeutic failure	20, 7
			G23012A	E484K	Higher infectivity in cell models, alone or in combination with other B.1.351 spike mutations (E); increased binding affinity of RBD to ACE2, alone or in combination with other B.1.351 spike mutations (P); greater spike S1-S2 cleavage (E); increased cell-to-cell fusion (E)	Possible increase in infectivity and transmissibility	17, 19, 18
					Together with E484K, lead to a moderate resistance to neutralization by convalescent sera (E); together with K417N and N501Y lead to a high resistance to neutralization by vaccine-elicited sera (E)	Possible immune evasion	18, 21
					Escape from neutralization by therapeutic monoclonal antibodies (E)	Possible therapeutic failure	7, 20, 22
			A23063T	N501Y	Mutation shared with B.1.1.7 and P.1 variants, and previously approached in this table.		
			C23664T	A701V	NF	NF	NF
	Orf3a	ORF3A	G25563T	Q57H	This variation also introduces an early stop codon to ORF3B causing the lack of 44 amino acids. This truncated protein loses the ability to inhibit interferon induction *in vitro* (E), although retains potent capacity of innate immune evasion in organoid models (E)	NF	23, 24
			C25904T	S171L	Alters the secondary structure and enhances the protein disorder (P)	Possible immune evasion	25
	E	Envelop protein	C26456T	P71L	Slightly stabilizes the structure of E protein (P)	NF	26
	N	Nucleocapsid protein	C28887T	T205I	NF	NF	NF
P.1 / Gamma	*ORF1ab*	NSP3	C3828T	S1188L	Associated to a persistently replicative phenotype of SARS-CoV-2 without cytopathic effect in cell model (E)	NF	27
			A5648C	K1795Q	NF	NF	NF

		NSP6	11288-11296 del	SGF 3675-3677 del	Mutation shared with B.1.1.7 variant, and previously approached in this table.		
		Helicase	G17259T	E5662D**	NF	NF	NF
	S	Spike protein	C21614T	L18F	Confers replicative advantage (P)	Possible increase in infectivity and transmissibility	28
					Escape from neutralization by a B-cell derived monoclonal antibody against the N-terminal domain of S protein (E)	Possible immune evasion	29
					Escape from neutralization by therapeutic monoclonal antibodies (E)	Possible therapeutic failure	30
			C21621A	T20N	Escape from neutralization by therapeutic monoclonal antibodies (E)	Possible therapeutic failure	30
			C21638T	P26S	Partially escapes from neutralization by a therapeutic monoclonal antibody (E)	Possible therapeutic failure	30
			G21974T	D138Y	Escape from neutralization by therapeutic monoclonal antibodies (E)	Possible therapeutic failure	30
			G22132T	R190S	NF	NF	NF
			A22812C	K417T	Increased binding affinity of RBD to ACE2 (P)	Possible increase in infectivity and transmissibility	17
			G23012A	E484K	Mutation shared with the B.1.351 variant, and previously approached in this table.		
			A23063T	N501Y	Mutation shared with B.1.1.7 and B.1.351 variants, and previously approached in this table.		
			C23525T	H655Y	Escape from neutralization by therapeutic monoclonal antibodies (E)	Possible therapeutic failure	31
			C24642T	T1027I	NF	NF	NF
	Orf3a	ORF3A	NF	G174C*	NF	NF	NF
	Orf8a	ORF8A	G28167A	E92K	NF	NF	NF
			28263insAACA	-	NF	NF	NF
	N	Nucleocapsid protein	C28512G	P80R	NF	NF	NF
B.1.617.2 / Delta	*S*	Spike protein	C21618G	T19R	NF	NF	NF
			T22917G	L452R	Escape from neutralization by therapeutic monoclonal antibodies (E)	Possible therapeutic failure	29<br>7

					Increase in virus entry in ACE2- and TMPRSS-expressing cell lines (E)	Possible increase in infectivity and transmissibility	32
					Increase in ACE2 binding (P)	Possible increase in infectivity and transmissibility	33
					Increase in the positive electrostatic potential surface possibly enhancing the interaction with the negatively charged ACE2 (P)	Possible increase in infectivity and transmissibility	34
			C22995A	T478K	Increase in ACE2 binding (P)	Possible increase in infectivity and transmissibility	33
					Increase in the positive electrostatic potential surface possibly enhancing the interaction with the negatively charged ACE2 (P)	Possible increase in infectivity and transmissibility	34
			C23604G	P681R	Enhanced syncytia formation, processing of S2 subunit and SARS-CoV-2 S-mediated fusion (E); Improve the furin cleavage efficiency of the Spike protein (E);	Possible increase in infectivity and transmissibility	5<br>35
					Increased virus-induced clinical manifestations in an animal model (E)	Possible increase in viral pathogenicity	35
					Escape from neutralization by therapeutic monoclonal antibodies and vaccine-elicited sera (E)	Possible therapeutic failure	35
			G24410A	D950N	NF	NF	NF
	Orf3a	ORF3A	C25469T	S26L	NF	NF	NF
	M	Membrane protein	T26767C	I82T	Associated to younger patients (E)	Possible increase in transmissibility between younger individuals	36
	Orf7a	ORF7A	T27638C	V82A	NF	NF	NF
			C27752T	T120I	NF	NF	NF
	N	Nucleocapsid protein	A28461G	D63G	NF	NF	NF
			G28881T	R203M	NF	NF	NF
			G29402T	D377Y	NF	NF	NF
B.1.1.529 / Omicron	*ORF1ab*	NSP5	C10029T	T3255I	NF	NF	NF
			C10449A	P3395H	NF	NF	NF

	RdRp	C14408T	P314L	NF	NF	NF
	NSP14	A18163G	I1566V	NF	NF	NF
S	Spike protein	G21987A	G142D	Alters the topography of the Spike's N-terminal domain, which may diminish antibody binding to this region (P).	Possible immune evasion	37
		G22578A	G339D	Partially escapes from neutralization by Sotrovimab and other antibodies (E); Reduced T-cell response (E); Alters the charge distribution of the surface of Spike's RBD, which may diminish antibody binding to this region (P).	Possible immune evasion and therapeutic failure	38<br>39<br>40
		T22679C	S373P	Alters the conformation of Spike protein, which may diminish antibody binding to this region (P).	Possible immune evasion	40
				Increases the binding affinity with human ACE2 (P).	Possible increase in infectivity and transmissibility	41
		C22686T	S375F	Escapes from neutralization by antibodies present in convalescent and vaccine-elicited sera (E); Alters the conformation of Spike protein, which may diminish antibody binding to this region (P).	Possible immune evasion	38<br>40
				Increases the binding affinity with human ACE2 (P).	Possible increase in infectivity and transmissibility	41<br>42
		G22813T	K417N	Mutation shared with the B.1.351 variant, and previously approached in this table.		
		T22882G	N440K	Alters the charge distribution of the surface of Spike's RBD, which may diminish antibody binding to this region (P). Escapes from neutralization by antibodies present in convalescent and vaccine-elicited sera (E).	Possible immune evasion	40<br>38
				Scape from neutralization by therapeutic monoclonal antibodies (E)	Possible therapeutic failure	43<br>44
				Increases the binding affinity with human ACE2 (P).	Possible increase in infectivity and transmissibility	42
		G22992A	S477N	NF	NF	NF
		C22995A	T478K	Mutation shared with the B.1.617.2 variant, and previously approached in this table.		
		G23012A	E484A	Mutation shared with the B.1.351 variant, and previously approached in this table.		

		A23040G	Q493R	Increases the binding affinity with human ACE2 (P).	Possible increase in infectivity and transmissibility	42
				Escape from neutralization by therapeutic monoclonal antibodies (E)	Possible therapeutic failure	43
		A23055G	Q498R	Increases the binding affinity with human ACE2 (P).	Possible increase in infectivity and transmissibility	41 42
				Escape from neutralization by therapeutic monoclonal antibodies (E)	Possible therapeutic failure	44
		A23063T	N501Y	Mutation shared with B.1.1.7 and P.1 variants, and previously approached in this table.		
		T23075C	Y505H	Decreases the binding affinity to ACE2 (P)	Possible reduction in infectivity and transmissibility	40
		C23525T	H655Y	Mutation shared with P.1 variant, and previously approached in this table.		
		T23599G	N679K	Possibly increase the O-linked glycosylation at the S1/S2 cleavage site, which may prevent recognition by proteases (P)	NF	45
		C23604A	P681H	Mutation shared with the B.1.117 variant, and previously approached in this table.		
		C23854A	N764K	NF	NF	NF
		G23948T	D796Y	NF	NF	NF
		A24424T	Q954H	NF	NF	NF
		T24469A	N969K	NF	NF	NF
E	Envelop protein	C26270I	T9I	NF	NF	NF
M	Membrane protein	C26577G	Q19E	NF	NF	NF
		G26709A	A63T	NF	NF	NF
Orf8	ORF8	C28144T	S84L	NF	NF	NF
N	Nucleocapsid protein	C28311T	P13L	Reduces responsiveness of T cell	Possible immune evasion	46
		28363 - 28371 del	RTQ 31-33 del	NF	NF	NF
		G28890A	R203K	NF	NF	NF
		G28883C	G204R	NF	NF	NF

Legend: NF- Not found; del- deletion; ins- insertion; (E)- Data obtained experimentally; (P)- Data computationally predicted; (S)- Suggested; * This mutation was not considered fixed in the variant population or was not described by the original article, but is considered defining by the PANGOLIN; ** This mutation was describe in the original article, but is not considered defining by the PANGOLIN.

References

(1) Xiao, C.; Mao, L.; Wang, Z.; Gao, L.; Zhu, G.; Su, J.; Chen, X.; Yuan, J.; Hu, Y.; Yin, Z.; Xie, J.; Ji, W.; Niu, H.; Gao, F.; Luo, O. J.; Xiao, L.; Wang, P.; Chen, G. SARS-CoV-2 variant B.1.1.7 caused HLA-A2. *iScience* **2022**, *25* (3), 103934. DOI: 10.1016/j.isci.2022.103934.
(2) Obermair, F. J.; Renoux, F.; Heer, S.; Lee, C. H.; Cereghetti, N.; Loi, M.; Maestri, G.; Haldner, Y.; Wuigk, R.; Iosefson, O.; Patel, P.; Triebel, K.; Kopf, M.; Swain, J.; Kisielow, J. High-resolution profiling of MHC II peptide presentation capacity reveals SARS-CoV-2 CD4 T cell targets and mechanisms of immune escape. *Sci Adv* **2022**, *8* (17), eabl5394. DOI: 10.1126/sciadv.abl5394.
(3) Bal, A.; Destras, G.; Gaymard, A.; Stefic, K.; Marlet, J.; Eymieux, S.; Regue, H.; Semanas, Q.; d'Aubarede, C.; Billaud, G.; Laurent, F.; Gonzalez, C.; Mekki, Y.; Valette, M.; Bouscambert, M.; Gaudy-Graffin, C.; Lina, B.; Morfin, F.; Josset, L. Two-step strategy for the identification of SARS-CoV-2 variant of concern 202012/01 and other variants with spike deletion H69-V70, France, August to December 2020. *Euro Surveill* **2021**, *26* (3). DOI: 10.2807/1560-7917.ES.2021.26.3.2100008.
(4) McCarthy, K. R.; Rennick, L. J.; Nambulli, S.; Robinson-McCarthy, L. R.; Bain, W. G.; Haidar, G.; Duprex, W. P. Recurrent deletions in the SARS-CoV-2 spike glycoprotein drive antibody escape. *Science* **2021**, *371* (6534), 1139-1142. DOI: 10.1126/science.abf6950.
(5) Liu, Y.; Liu, J.; Plante, K. S.; Plante, J. A.; Xie, X.; Zhang, X.; Ku, Z.; An, Z.; Scharton, D.; Schindewolf, C.; Widen, S. G.; Menachery, V. D.; Shi, P. Y.; Weaver, S. C. The N501Y spike substitution enhances SARS-CoV-2 infection and transmission. *Nature* **2021**. DOI: 10.1038/s41586-021-04245-0.
(6) Elfiky, A. A.; Ibrahim, I. M. Host-cell recognition through GRP78 is enhanced in the new UK variant of SARS-CoV-2, in silico. *J Infect* **2021**, *82* (5), 186-230. DOI: 10.1016/j.jinf.2021.01.015.
(7) Starr, T. N.; Greaney, A. J.; Hilton, S. K.; Ellis, D.; Crawford, K. H. D.; Dingens, A. S.; Navarro, M. J.; Bowen, J. E.; Tortorici, M. A.; Walls, A. C.; King, N. P.; Veesler, D.; Bloom, J. D. Deep Mutational Scanning of SARS-CoV-2 Receptor Binding Domain Reveals Constraints on Folding and ACE2 Binding. *Cell* **2020**, *182* (5), 1295-1310.e1220. DOI: 10.1016/j.cell.2020.08.012.
(8) Teruel, N.; Mailhot, O.; Najmanovich, R. J. Modelling conformational state dynamics and its role on infection for SARS-CoV-2 Spike protein variants. *PLoS Comput Biol* **2021**, *17* (8), e1009286. DOI: 10.1371/journal.pcbi.1009286.
(9) Thomson, E. C.; Rosen, L. E.; Shepherd, J. G.; Spreafico, R.; da Silva Filipe, A.; Wojcechowskyj, J. A.; Davis, C.; Piccoli, L.; Pascall, D. J.; Dillen, J.; Lytras, S.; Czudnochowski, N.; Shah, R.; Meury, M.; Jesudason, N.; De Marco, A.; Li, K.; Bassi, J.; O'Toole, A.; Pinto, D.; Colquhoun, R. M.; Culap, K.; Jackson, B.; Zatta, F.; Rambaut, A.; Jaconi, S.; Sreenu, V. B.; Nix, J.; Zhang, I.; Jarrett, R. F.; Glass, W. G.; Beltramello, M.; Nomikou, K.; Pizzuto, M.; Tong, L.; Cameroni, E.; Croll, T. I.; Johnson, N.; Di Iulio, J.; Wickenhagen, A.; Ceschi, A.; Harbison, A. M.; Mair, D.; Ferrari, P.; Smollett, K.; Sallusto, F.; Carmichael, S.; Garzoni, C.; Nichols, J.; Galli, M.; Hughes, J.; Riva, A.; Ho, A.; Schiuma, M.; Semple, M. G.; Openshaw, P. J. M.; Fadda, E.; Baillie, J. K.; Chodera, J. D.; Rihn, S. J.; Lycett, S. J.; Virgin, H. W.; Telenti, A.; Corti, D.; Robertson, D. L.; Snell, G. Circulating SARS-CoV-2 spike N439K variants maintain fitness while evading antibody-mediated immunity. *Cell* **2021**, *184* (5), 1171-1187.e1120. DOI: 10.1016/j.cell.2021.01.037.
(10) Zhao, S.; Lou, J.; Cao, L.; Zheng, H.; Chong, M. K. C.; Chen, Z.; Chan, R. W. Y.; Zee, B. C. Y.; Chan, P. K. S.; Wang, M. H. Quantifying the transmission advantage associated with N501Y substitution of SARS-CoV-2 in the UK: an early data-driven analysis. *J Travel Med* **2021**, *28* (2). DOI: 10.1093/jtm/taab011.
(11) Wang, Z.; Schmidt, F.; Weisblum, Y.; Muecksch, F.; Barnes, C. O.; Finkin, S.; Schaefer-Babajew, D.; Cipolla, M.; Gaebler, C.; Lieberman, J. A.; Oliveira, T. Y.; Yang, Z.; Abernathy, M. E.; Huey-Tubman, K. E.; Hurley, A.; Turroja, M.; West, K. A.; Gordon, K.; Millard, K. G.; Ramos, V.; Da Silva, J.; Xu, J.; Colbert, R. A.; Patel, R.; Dizon, J.; Unson-O'Brien, C.; Shimeliovich, I.; Gazumyan, A.; Caskey, M.; Bjorkman, P. J.; Casellas, R.; Hatziioannou, T.; Bieniasz, P. D.; Nussenzweig, M. C.

mRNA vaccine-elicited antibodies to SARS-CoV-2 and circulating variants. *Nature* **2021**, *592* (7855), 616-622. DOI: 10.1038/s41586-021-03324-6.
(12) Warwicker, J. A model for pH coupling of the SARS-CoV-2 spike protein open/closed equilibrium. *Brief Bioinform* **2021**, *22* (2), 1499-1507. DOI: 10.1093/bib/bbab056.
(13) Lubinski, B.; Fernandes, M. H. V.; Frazier, L.; Tang, T.; Daniel, S.; Diel, D. G.; Jaimes, J. A.; Whittaker, G. R. Functional evaluation of the P681H mutation on the proteolytic activation of the SARS-CoV-2 variant B.1.1.7 (Alpha) spike. *iScience* **2022**, *25* (1), 103589. DOI: 10.1016/j.isci.2021.103589.
(14) Hussain, M.; Shabbir, S.; Amanullah, A.; Raza, F.; Imdad, M. J.; Zahid, S. Immunoinformatic analysis of structural and epitope variations in the spike and Orf8 proteins of SARS-CoV-2/B.1.1.7. *J Med Virol* **2021**, *93* (7), 4461-4468. DOI: 10.1002/jmv.26931.
(15) Singh, J.; Samal, J.; Kumar, V.; Sharma, J.; Agrawal, U.; Ehtesham, N. Z.; Sundar, D.; Rahman, S. A.; Hira, S.; Hasnain, S. E. Structure-Function Analyses of New SARS-CoV-2 Variants B.1.1.7, B.1.351 and B.1.1.28.1: Clinical, Diagnostic, Therapeutic and Public Health Implications. *Viruses* **2021**, *13* (3). DOI: 10.3390/v13030439.
(16) Rehman, S.; Mahmood, T.; Aziz, E.; Batool, R. Identification of novel mutations in SARS-COV-2 isolates from Turkey. *Arch Virol* **2020**, *165* (12), 2937-2944. DOI: 10.1007/s00705-020-04830-0.
(17) Khan, A.; Zia, T.; Suleman, M.; Khan, T.; Ali, S. S.; Abbasi, A. A.; Mohammad, A.; Wei, D. Q. Higher infectivity of the SARS-CoV-2 new variants is associated with K417N/T, E484K, and N501Y mutants: An insight from structural data. *J Cell Physiol* **2021**, *236* (10), 7045-7057. DOI: 10.1002/jcp.30367.
(18) Kuzmina, A.; Khalaila, Y.; Voloshin, O.; Keren-Naus, A.; Boehm-Cohen, L.; Raviv, Y.; Shemer-Avni, Y.; Rosenberg, E.; Taube, R. SARS-CoV-2 spike variants exhibit differential infectivity and neutralization resistance to convalescent or post-vaccination sera. *Cell Host Microbe* **2021**, *29* (4), 522-528.e522. DOI: 10.1016/j.chom.2021.03.008.
(19) Kim, Y. J.; Jang, U. S.; Soh, S. M.; Lee, J. Y.; Lee, H. R. The Impact on Infectivity and Neutralization Efficiency of SARS-CoV-2 Lineage B.1.351 Pseudovirus. *Viruses* **2021**, *13* (4). DOI: 10.3390/v13040633.
(20) Li, Q.; Nie, J.; Wu, J.; Zhang, L.; Ding, R.; Wang, H.; Zhang, Y.; Li, T.; Liu, S.; Zhang, M.; Zhao, C.; Liu, H.; Nie, L.; Qin, H.; Wang, M.; Lu, Q.; Li, X.; Liu, J.; Liang, H.; Shi, Y.; Shen, Y.; Xie, L.; Qu, X.; Xu, W.; Huang, W.; Wang, Y. SARS-CoV-2 501Y.V2 variants lack higher infectivity but do have immune escape. *Cell* **2021**, *184* (9), 2362-2371.e2369. DOI: 10.1016/j.cell.2021.02.042.
(21) Garcia-Beltran, W. F.; Lam, E. C.; St Denis, K.; Nitido, A. D.; Garcia, Z. H.; Hauser, B. M.; Feldman, J.; Pavlovic, M. N.; Gregory, D. J.; Poznansky, M. C.; Sigal, A.; Schmidt, A. G.; Iafrate, A. J.; Naranbhai, V.; Balazs, A. B. Multiple SARS-CoV-2 variants escape neutralization by vaccine-induced humoral immunity. *Cell* **2021**, *184* (9), 2372-2383.e2379. DOI: 10.1016/j.cell.2021.03.013.
(22) Liu, H.; Wei, P.; Zhang, Q.; Chen, Z.; Aviszus, K.; Downing, W.; Peterson, S.; Reynoso, L.; Downey, G. P.; Frankel, S. K.; Kappler, J.; Marrack, P.; Zhang, G. 501Y.V2 and 501Y.V3 variants of SARS-CoV-2 lose binding to bamlanivimab. *MAbs* **2021**, *13* (1), 1919285. DOI: 10.1080/19420862.2021.1919285.
(23) Chu, D. K. W.; Hui, K. P. Y.; Gu, H.; Ko, R. L. W.; Krishnan, P.; Ng, D. Y. M.; Liu, G. Y. Z.; Wan, C. K. C.; Cheung, M. C.; Ng, K. C.; Nicholls, J. M.; Tsang, D. N. C.; Peiris, M.; Chan, M. C. W.; Poon, L. L. M. Introduction of ORF3a-Q57H SARS-CoV-2 Variant Causing Fourth Epidemic Wave of COVID-19, Hong Kong, China. *Emerg Infect Dis* **2021**, *27* (5), 1492-1495. DOI: 10.3201/eid2705.210015.
(24) Lam, J. Y.; Yuen, C. K.; Ip, J. D.; Wong, W. M.; To, K. K.; Yuen, K. Y.; Kok, K. H. Loss of orf3b in the circulating SARS-CoV-2 strains. *Emerg Microbes Infect* **2020**, *9* (1), 2685-2696. DOI: 10.1080/22221751.2020.1852892.
(25) Azad, G. K.; Khan, P. K. Variations in Orf3a protein of SARS-CoV-2 alter its structure and function. *Biochem Biophys Rep* **2021**, *26*, 100933. DOI: 10.1016/j.bbrep.2021.100933.

(26) Rahman, M. S.; Hoque, M. N.; Islam, M. R.; Islam, I.; Mishu, I. D.; Rahaman, M. M.; Sultana, M.; Hossain, M. A. Mutational insights into the envelope protein of SARS-CoV-2. *Gene Rep* **2021**, *22*, 100997. DOI: 10.1016/j.genrep.2020.100997.
(27) Caccuri, F.; Zani, A.; Messali, S.; Giovanetti, M.; Bugatti, A.; Campisi, G.; Filippini, F.; Scaltriti, E.; Ciccozzi, M.; Fiorentini, S.; Caruso, A. A persistently replicating SARS-CoV-2 variant derived from an asymptomatic individual. *J Transl Med* **2020**, *18* (1), 362. DOI: 10.1186/s12967-020-02535-1.
(28) Grabowski, F.; Kochańczyk, M.; Lipniacki, T. L18F substrain of SARS-CoV-2 VOC-202012/01 is rapidly spreading in England. In *medRxiv*, 2021.
(29) McCallum, M.; De Marco, A.; Lempp, F. A.; Tortorici, M. A.; Pinto, D.; Walls, A. C.; Beltramello, M.; Chen, A.; Liu, Z.; Zatta, F.; Zepeda, S.; di Iulio, J.; Bowen, J. E.; Montiel-Ruiz, M. Zhou, J.; Rosen, L. E.; Bianchi, S.; Guarino, B.; Fregni, C. S.; Abdelnabi, R.; Foo, S. C.; Rothlauf, P. W.; Bloyet, L. M.; Benigni, F.; Cameroni, E.; Neyts, J.; Riva, A.; Snell, G.; Telenti, A.; Whelan, S. P. J.; Virgin, H. W.; Corti, D.; Pizzuto, M. S.; Veesler, D. N-terminal domain antigenic mapping reveals a site of vulnerability for SARS-CoV-2. *Cell* **2021**, *184* (9), 2332-2347.e2316. DOI: 10.1016/j.cell.2021.03.028.
(30) Wang, P.; Casner, R. G.; Nair, M. S.; Wang, M.; Yu, J.; Cerutti, G.; Liu, L.; Kwong, P. D.; Huang, Y.; Shapiro, L.; Ho, D. D. Increased resistance of SARS-CoV-2 variant P.1 to antibody neutralization. *Cell Host Microbe* **2021**, *29* (5), 747-751.e744. DOI: 10.1016/j.chom.2021.04.007.
(31) Baum, A.; Fulton, B. O.; Wloga, E.; Copin, R.; Pascal, K. E.; Russo, V.; Giordano, S.; Lanza, K.; Negron, N.; Ni, M.; Wei, Y.; Atwal, G. S.; Murphy, A. J.; Stahl, N.; Yancopoulos, G. D.; Kyratsous, C. A. Antibody cocktail to SARS-CoV-2 spike protein prevents rapid mutational escape seen with individual antibodies. *Science* **2020**, *369* (6506), 1014-1018. DOI: 10.1126/science.abd0831.
(32) Deng, X.; Garcia-Knight, M. A.; Khalid, M. M.; Servellita, V.; Wang, C.; Morris, M. K.; Sotomayor-González, A.; Glasner, D. R.; Reyes, K. R.; Gliwa, A. S.; Reddy, N. P.; Sanchez San; Martin, C.; Federman, S.; Cheng, J.; Balcerek, J.; Taylor, J.; Streithorst, J. A.; Miller, S.; Sreekumar, B.; Chen, P. Y.; Schulze-Gahmen, U.; Taha, T. Y.; Hayashi, J. M.; Simoneau, C. R.; Kumar, G. R. McMahon, S.; Lidsky, P. V.; Xiao, Y.; Hemarajata, P.; Green, N. M.; Espinosa, A.; Kath, C.; Haw, M.; Bell, J.; Hacker, J. K.; Hanson, C.; Wadford, D. A.; Anaya, C.; Ferguson, D.; Frankino, P. A.; Shivram, H.; Lareau, L. F.; Wyman, S. K.; Ott, M.; Andino, R.; Chiu, C. Y.Transmission, infectivity, and neutralization of a spike L452R SARS-CoV-2 variant. *Cell* **2021**, *184* (13), 3426-3437.e3428. DOI: 10.1016/j.cell.2021.04.025.
(33) Cherian, S.; Potdar, V.; Jadhav, S.; Yadav, P.; Gupta, N.; Das, M.; Rakshit, P.; Singh, S.; Abraham, P.; Panda, S. SARS-CoV-2 Spike Mutations, L452R, T478K, E484Q and P681R, in the Second Wave of COVID-19 in Maharashtra, India. *Microorganisms* **2021**, *9* (7). DOI: 10.3390/microorganisms9071542.
(34) Pascarella, S.; Ciccozzi, M.; Zella, D.; Bianchi, M.; Benedetti, F.; Benvenuto, D.; Broccolo, F.; Cauda, R.; Caruso, A.; Angeletti, S.; Giovanetti, M.; Cassone, A. SARS-CoV-2 B.1.617 Indian variants: Are electrostatic potential changes responsible for a higher transmission rate? *J Med Virol* **2021**. DOI: 10.1002/jmv.27210.
(35) Saito, A.; Irie, T.; Suzuki, R.; Maemura, T.; Nasser, H.; Uriu, K.; Kosugi, Y.; Shirakawa, K.; Sadamasu, K.; Kimura, I.; Ito, J.; Wu, J.; Iwatsuki-Horimoto, K.; Ito, M.; Yamayoshi, S.; Loeber, S. Tsuda, M.; Wang, L.; Ozono, S.; Butlertanaka, E. P.; Tanaka, Y. L.; Shimizu, R.; Shimizu, K.; Yoshimatsu, K.; Kawabata, R.; Sakaguchi, T.; Tokunaga, K.; Yoshida, I.; Asakura, H.; Nagashima, M.; Kazuma, Y.; Nomura, R.; Horisawa, Y.; Yoshimura, K.; Takaori-Kondo, A.; Imai, M.; Tanaka, S.; Nakagawa, S.; Ikeda, T.; Fukuhara, T.; Kawaoka, Y.; Sato, K. Enhanced fusogenicity and pathogenicity of SARS-CoV-2 Delta P681R mutation. *Nature* **2022**, *602* (7896), 300-306. DOI: 10.1038/s41586-021-04266-9.
(36) Shen, L.; Bard, J. D.; Triche, T. J.; Judkins, A. R.; Biegel, J. A.; Gai, X. Emerging variants of concern in SARS-CoV-2 membrane protein: a highly conserved target with potential pathological and therapeutic implications. *Emerg Microbes Infect* **2021**, *10* (1), 885-893. DOI: 10.1080/22221751.2021.1922097.

(37) Shen, L.; Triche, T.; Bard, J.; Biegel, J.; Judkins, A.; Gai, X. Spike Protein NTD mutation G142D in SARS-CoV-2 Delta VOC lineages is associated with frequent back mutations, increased viral loads, and immune evasion. In *medRxiv*, 2021.
(38) Cao, Y.; Wang, J.; Jian, F.; Xiao, T.; Song, W.; Yisimayi, A.; Huang, W.; Li, Q.; Wang, P.; An, R.; Wang, Y.; Niu, X.; Yang, S.; Liang, H.; Sun, H.; Li, T.; Yu, Y.; Cui, Q.; Liu, S.; Yang, X.; Du, S.; Zhang, Z.; Hao, X.; Shao, F.; Jin, R.; Wang, X.; Xiao, J.; Xie, X. S. Omicron escapes the majority of existing SARS-CoV-2 neutralizing antibodies. *Nature* **2021**. DOI: 10.1038/s41586-021-04385-3.
(39) Li, Y.; Wang, X.; Jin, J.; Ma, Z.; Liu, Y.; Zhang, X.; Su, B. T-cell responses to SARS-CoV-2 Omicron spike epitopes with mutations after the third booster dose of an inactivated vaccine. *J Med Virol* **2022**, *94* (8), 3998-4004. DOI: 10.1002/jmv.27814.
(40) Jawad, B.; Adhikari, P.; Podgornik, R.; Ching, W. Y. Binding Interactions between Receptor-Binding Domain of Spike Protein and Human Angiotensin Converting Enzyme-2 in Omicron Variant. *J Phys Chem Lett* **2022**, *13* (17), 3915-3921. DOI: 10.1021/acs.jpclett.2c00423.
(41) Kumar, S.; Thambiraja, T. S.; Karuppanan, K.; Subramaniam, G. Omicron and Delta variant of SARS-CoV-2: A comparative computational study of spike protein. *J Med Virol* **2022**, *94* (4), 1641-1649. DOI: 10.1002/jmv.27526.
(42) Hwang, S.; Baek, S. H.; Park, D. Interaction Analysis of the Spike Protein of Delta and Omicron Variants of SARS-CoV-2 with hACE2 and Eight Monoclonal Antibodies Using the Fragment Molecular Orbital Method. *J Chem Inf Model* **2022**, *62* (7), 1771-1782. DOI: 10.1021/acs.jcim.2c00100.
(43) Liu, L.; Iketani, S.; Guo, Y.; Chan, J. F.; Wang, M.; Luo, Y.; Chu, H.; Huang, Y.; Nair, M. S.; Yu, J.; Chik, K. K.; Yuen, T. T.; Yoon, C.; To, K. K.; Chen, H.; Yin, M. T.; Sobieszczyk, M. E.; Wang, H. H.; Sheng, Z.; Yuen, K. Y.; Ho, D. D. Striking Antibody Evasion Manifested by the Omicron Variant of SARS-CoV-2. *Nature* **2021**. DOI: 10.1038/s41586-021-04388-0.
(44) Nguyen, H.; Lan, P. D.; Nissley, D. A.; O'Brien, E. P.; Li, M. S. Cocktail of REGN Antibodies Binds More Strongly to SARS-CoV-2 Than Its Components, but the Omicron Variant Reduces Its Neutralizing Ability. *J Phys Chem B* **2022**, *126* (15), 2812-2823. DOI: 10.1021/acs.jpcb.2c00708.
(45) Beaudoin, C. A.; Pandurangan, A. P.; Kim, S. Y.; Hamaia, S. W.; Huang, C. L.; Blundell, T. L.; Vedithi, S. C.; Jackson, A. P. In silico analysis of mutations near S1/S2 cleavage site in SARS-CoV-2 spike protein reveals increased propensity of glycosylation in Omicron strain. *J Med Virol* **2022**. DOI: 10.1002/jmv.27845.
(46) de Silva, T. I.; Liu, G; Lindsey, B. B; Dong, D; Moore, S. C; Hsu, N. S; Shah, D; Wellington, D; Mentzer, A. J; Angyal, A; Brown, R; Parker, M. D; Ying, Z; Yao, X; Turtle, L; Dunachie, S; Maini, M. K.; Ogg, G; Knight, J. C; Peng, Y; Rowland-Jones, S. L; Dong, T. The impact of viral mutations on recognition by SARS-CoV-2 specific T cells. *iScience* **2021**, *24* (11), 103353. DOI: 10.1016/j.isci.2021.103353.
